# Diverse *Streptococcus pneumoniae* Strains Drive a Mucosal-Associated Invariant T-Cell Response Through Major Histocompatibility Complex class I–Related Molecule–Dependent and Cytokine-Driven Pathways

**DOI:** 10.1093/infdis/jix647

**Published:** 2017-12-15

**Authors:** Ayako Kurioka, Bonnie van Wilgenburg, Reza Rezaei Javan, Ryan Hoyle, Andries J van Tonder, Caroline L Harrold, Tianqi Leng, Lauren J Howson, Dawn Shepherd, Vincenzo Cerundolo, Angela B Brueggemann, Paul Klenerman

**Affiliations:** 1 Nuffield Department of Medicine, University of Oxford, Oxford; 2 MRC Human Immunology Unit, Weatherall Institute of Molecular Medicine, University of Oxford, Oxford; 3 Department of Medicine, Imperial College London, London, United Kingdom

**Keywords:** MAIT cells, pneumococcus, riboflavin, innate, macrophages, cytokines, MR1, T cells

## Abstract

Mucosal-associated invariant T (MAIT) cells represent an innate T-cell population that can recognize ligands generated by the microbial riboflavin synthesis pathway, presented via the major histocompatibility complex class I–related molecule (MR1). *Streptococcus pneumoniae* is a major human pathogen that is also associated with commensal carriage; thus, host control at the mucosal interface is critical. The recognition of pneumococci by MAIT cells has not been defined nor have the genomics and transcriptomics of the riboflavin operon. We observed robust recognition of pneumococci by MAIT cells, using both MR1-dependent and MR1-independent pathways. The pathway used was dependent on the antigen-presenting cell. The riboflavin operon was highly conserved across a range of 571 pneumococci from 39 countries, dating back to 1916, and different versions of the riboflavin operon were also identified in related *Streptococcus* species. These data indicate an important functional relationship between MAIT cells and pneumococci.

## Teaser

Human MAIT cells respond to diverse pneumococcal strains. These share a conserved riboflavin operon providing the MAIT-cell ligand, although MAIT cell triggering in vitro depends on the metabolic state of the bacteria and the nature of the antigen presenting cell.

The pneumococcus is the most common cause of community-acquired pneumonia and is associated with significant morbidity and mortality, especially among young children and older adults [1, 2]. Pneumococci also cause invasive diseases, such as meningitis and bacteremia, and upper respiratory tract infections, such as otitis media and sinusitis [3]. Antimicrobial-resistant strains are widespread and pose problems in the treatment of infections, leading the World Health Organization to include pneumococci on their list of priority pathogens [4]. The available pneumococcal conjugate vaccines prompt immune responses to polysaccharide capsules (differentiated as serotypes) and are highly effective at preventing invasive pneumococcal disease due to vaccine-serotype strains; however, current vaccines only protect against a small number of the possible serotypes, leading to increases in rates of disease from nonvaccine-serotype pneumococci [5, 6]. Therefore, pneumococcal disease remains a serious problem, and better understanding of the host defense against pneumococci may facilitate design of novel interventions.

There is increasing appreciation of the role of unconventional T cells in orchestrating early responses to pathogens [7]. Mucosal-associated invariant T (MAIT) cells are a recently described innate T-cell population, abundant in the lung, blood, and liver [8–10]. They express a semi-invariant T-cell receptor (TCR) chain, Vα7.2-Jα33/Jα12/Jα20, paired with a limited repertoire of Vβ chains [11]. This TCR can recognize ligands presented by the conserved major histocompatibility complex (MHC)–related protein 1 (MR1) [8]. MR1 binds vitamin B–based precursors from the riboflavin-biosynthesis pathway, conserved across various bacteria and fungi [10, 12, 13]. Human MAIT cells can also respond to innate cytokines even without TCR signaling [14, 15]. Upon activation, they produce immunomodulatory cytokines, including interferon γ (IFN-γ), tumor necrosis factor α, and interleukin 17.

MAIT cells are critical for the control of bacterial infections in mice, particularly in the lungs [16–18]. For instance, aerosol-based infection models with *Mycobacterium bovis* bacillus Calmette-Guérin and the live vaccine strain of *Francisella tularensis* demonstrated that MAIT cells were essential for the early control of the bacterial burdens [18, 19]. Indeed, early lung MAIT cell activation by *F. tularensis* was required for the differentiation of dendritic cells and subsequent recruitment of activated CD4^+^ T cells [20]. Thus, rapid activation of MAIT cells in response to pulmonary bacteria is critical for bridging innate and adaptive systems.

Despite these data, it remains unclear whether MAIT cells play a role in the defense against pneumococcal infection. Here, we show that MAIT cells responded to pneumococci in an MR1-dependent manner in the presence of macrophages but not monocytes and that this was dependent on costimulation provided by innate cytokines. Furthermore, using a population-level genomics approach, we found that the riboflavin synthesis pathway is ubiquitous and highly conserved amongst pneumococci. Riboflavin operon genes were also found among other nonpneumococcal *Streptococcus* species, including *Streptococcus agalactiae* (group B streptococci), which suggests that the observations made here are relevant to other human-associated *Streptococcus* species infections.

## METHODS

### Cells

Whole-blood specimens were obtained from leukocyte cones (NHS Blood and Transplant), and peripheral blood mononuclear cells (PBMCs) were isolated by density gradient centrifugation (Lymphoprep Axis-Shield). All samples were collected with written consent and local research ethics committee approval (COREC 04.OXA.010). Monocyte-derived macrophages were generated by enriching for monocytes using CD14 microbeads (Miltenyi Biotech) before culturing with 50 ng/mL granulocyte-macrophage colony-stimulating factor (Miltenyi Biotech) in Roswell Park Memorial Institute 1640 medium, penicillin/streptomycin, L-glutamine, and 10% human serum (all from Sigma Aldrich) for 6–8 days. For details of the Jurkat-MAIT cell line, see the Supplementary Methods.

### Bacteria

Pneumococcal Molecular Epidemiological Network (PMEN) strains (Supplementary Methods) were cultured from freezer stocks to Columbia blood agar plates (Oxoid), incubated overnight, and then transferred to Todd Hewitt broth (THB; Sigma Aldrich) with 0.5% yeast extract (THB-Y; Sigma Aldrich) and incubated overnight, unless indicated otherwise. Where indicated, bacteria were grown in riboflavin-free medium (ie, riboflavin assay medium [BD Difco] or THB alone) [21]. *Escherichia coli* (DH5a; Invitrogen) was cultured in LB medium overnight in a shaking incubator.

Pneumococci or *E. coli* were fixed in 2% paraformaldehyde for 15 minutes and washed extensively (except in a single set of experiments in which live bacteria were used for comparison). A negative control was prepared identically.

### In Vitro Stimulation of MAIT Cells

THP1 cells (ATCC, Middlesex, United Kingdom) were incubated overnight with paraformaldehyde-fixed pneumococci or *E. coli* at a ratio of 30 bacteria/cell or with sterile control. For stimulation experiments, in which activation of MAIT cells was examined (eg, IFN-γ production), THP1 cells were washed, and PBMCs or enriched CD8^+^ T cells were added to THP1 cells overnight. Brefeldin A (eBioscience) was added for the final 4 hours of the stimulation before intracellular cytokine staining. For internal staining, cells were fixed with 1% formaldehyde (Sigma Aldrich) and permeabilized with permeabilization buffer (eBioscience). Alternatively, for the assessment of degranulation, anti-CD107a PE-Cy7 (BioLegend) was added from the start of the stimulation. For blocking experiments, anti-MR1, anti–interleukin 12p40/70 (IL-12p40/70), and anti–interleukin 18 (IL-18) antibodies (all BioLegend) or the appropriate isotype controls were added for the duration of the experiment. Cells were acquired on the MACSQuant Analyser (Miltenyi Biotech) and analyzed using FlowJo v9.8 (TreeStar). Graphs and statistical analyses were completed using GraphPad Prism 6. All data are presented as mean values with standard errors of the mean (SEMs). For further details and antibodies used, see the Supplementary Methods.

### RNA Sequencing

Pneumococcal strain 2/2 was cultured in brain-heart infusion broth and incubated at 40°C for 6 hours to mimic heat shock. Identical experimental controls were incubated at 37°C. Broth cultures at 2, 3, 4, 5, and 6 hours were removed from the incubator, and RNAprotect Bacteria Reagent (Qiagen) was added to stabilize the RNA. RNA was extracted from the samples, using the Promega Maxwell 16 Instrument and LEV simplyRNA Cells purification kit, following the manufacturer’s protocol. Extracted RNA samples were sent to the Oxford Genomics Centre for processing. Library preps were made using RNA-Seq Ribozero kits (Illumina), and sequencing was performed on the MiSeq (Illumina). The Gene Expression Omnibus accession number is pending.

The sequenced forward and reverse reads were paired and mapped to pneumococcal strain 2/2 genome, using Bowtie2 with the highest-sensitivity option [22]. Differential gene expression was analyzed in Geneious, version 9.1 (Biomatters), using the DESeq method [23]. Genes with an adjusted *P* value of <.05 were considered to be differentially expressed.

### Compilation of the Genome Data Sets

Two large genome data sets were compiled for this study, and data were stored in a BIGSdb database [24]. The pneumococcal data set consisted of 571 historical and modern genomes isolated during 1916–2009 from people of all ages residing in 39 different countries. The pneumococci were recovered from individuals with carriage and those with disease, and 89 serotypes and 296 multilocus sequence types were represented in this data set (Supplementary Table 1). A total of 486 pneumococcal genome sequences were compiled from previously published studies or were downloaded from GenBank [25]. The remaining 85 pneumococcal genomes were recently sequenced. Pneumococcal cultures were prepared as described above, before DNA was extracted using the Promega Maxwell 16 Instrument and Buccal Swab LEV DNA purification kits according to the manufacturers’ protocols. DNA extracts were sent to the Oxford Genomics Centre, where libraries were made and DNA was sequenced on the Ilumina platform. Velvet was used to make de novo genome assemblies, which were further improved using SSPACE and GapFiller [26–28].

The nonpneumococcal *Streptococcus* species data set contained 834 genomes of 69 different streptococcal species (Supplementary Table 2). Thirty-four genomes were newly sequenced as described above, and the rest were downloaded from the ribosomal multilocus sequence typing database [29]. Further details are provided in the Supplementary Methods.

## RESULTS

### Pneumococci Possess a Highly Conserved Riboflavin Synthesis Operon That Is Upregulated With Heat Stress

**Figure 1.**
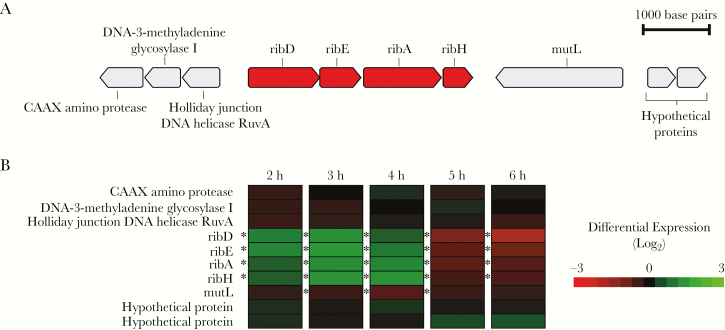
Genetic and transcriptomic data related to the riboflavin operon in pneumococci. *A*, The riboflavin operon is depicted with riboflavin genes *ribD*, *ribE*, *ribA*, and *ribH* (red) and flanking genes (gray). *B*, RNA expression data at 5 time points (2–6 hours after initial incubation) are illustrated for each riboflavin gene and the flanking genes. Genes marked with differential expression levels in green were upregulated, and those in red were downregulated during incubation at 40°C as compared to normal incubation at 37°C. *P < .05.

The genes encoding the riboflavin biosynthetic enzymes of pneumococci (*ribD*, *ribE*, *ribA*, and *ribH*) were found to be clustered together in the same orientation in a predicted 3.4-Kb operon structure (Figure 1A). The prevalence and sequence diversity of the coding regions of the riboflavin genes were investigated in a large, global, and historical genome data set of pneumococci isolated between 1916 and 2008 from people of all ages residing in 36 different countries. A total of 561 pneumococcal genomes (98.2%) contained the riboflavin operon. Nine of 10 genomes that lacked the operon were of a single multilocus sequence type (ST^serotype^), ST13^14/nontypable^, and the other belonged to ST695^4^ (Supplementary Table 1). All genes in the riboflavin operon were found to be highly conserved: nucleotide and amino acid sequence identity were >99% (Table 1). The dN/dS analysis revealed a higher prevalence of synonymous versus nonsynonymous mutations, supporting the importance of maintaining the riboflavin operon (Table 1).

**Table 1.** Description of the 4 Riboflavin Operon Gene Sequences Within 571 Pneumococcal Genomes

Gene	Present, No. (%)^a^	Nucleotide Pair-Wise Identity, %	Amino Acid Pair-Wise Identity, %	Mean dN/dS	Gene Annotation
*ribD*	559 (98.2)	99.6	99.8	0.36	Diaminohydroxyphosphoribosylamino-pyrimidine deaminase
*ribE*	561 (98.2)	99.6	99.9	0.28	Riboflavin synthase
*ribA*	561 (98.2)	99.7	99.9	0.42	3,4-dihydroxy-2-butanone-4-phosphate synthase
*ribH*	559 (98.2)	99.5	99.1	0.12	6,7-dimethyl-8-ribityllumazine synthase

^a^Two genomes possessed sequence assembly gaps in *ribD* and *ribH,* and 10 genomes were missing all 4 riboflavin genes (see Results).

Total bacterial RNA sequencing was performed on RNA extracted from a pneumococcus that was subjected to metabolic stress by incubation at a higher temperature than normal (40°C vs 37°C). Differential expression analysis revealed that all of the riboflavin operon genes were significantly upregulated after 2–4 hours of incubation under heat stress as compared to the control (Figure 1B). Subsequently, the riboflavin operon was found to be significantly downregulated after 5–6 hours of incubation. The concurrent increase and decrease in the expression of the 4 riboflavin genes suggested that these genes are transcriptionally coupled.

### MAIT Cells Are Activated by Pneumococci

**Figure 2.**
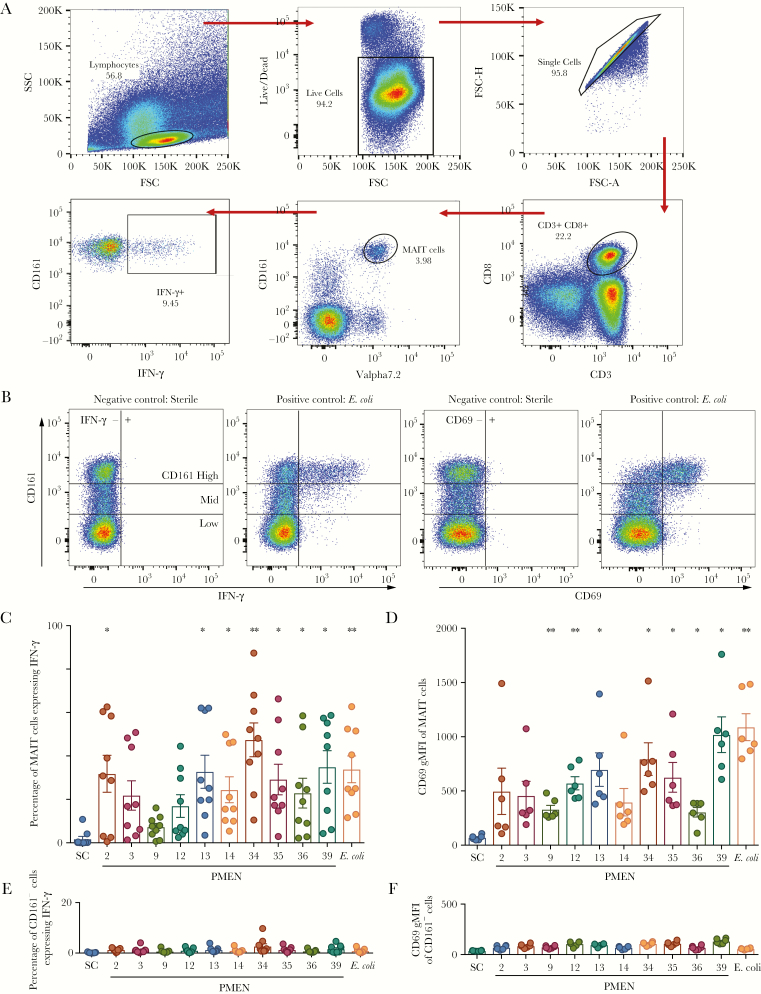
Mucosal-associated invariant T (MAIT) cells are activated by pneumococci. *A*, Gating strategy for analysis of MAIT cell–derived interferon γ (IFN-γ) following activation of peripheral blood mononuclear cells. *B*, Example fluorescence-activated cell-sorting plots showing upregulation of CD69 and IFN-γ in unstimulated and stimulated MAIT cells (gating on CD3^+^CD8^+^ live T cells). *C* and *D*, Ten Pneumococcal Molecular Epidemiological Network (PMEN) reference strains were used to probe the activation of MAIT cells following coculture of peripheral blood mononuclear cells in the presence of the monocytic cell line, THP1. *Escherichia coli* was added as a positive control. Frequency of cells expressing IFN-γ among MAIT cells (*C*) or CD161^−^CD8^+^ T cells (*D*) are shown (n = 9). *E* and *F*, CD69 expression measured by geometric mean fluorescence intensity (gMFI) in MAIT cells (*E*) or CD161^−^CD8^+^ T cells (*F*) are shown (n = 6). **P < .01 and *P < .05 by repeated measures 1-way analysis of variance with the Dunnett multiple comparisons test, compared with the sterile control (SC). Numbers indicate the PMEN reference strains. FSC, forward scatter; SSC, side scatter.

Human MAIT cell responses to bacteria can be readily analyzed in vitro, using fluorescence-activated cell-sorting analysis of PBMCs. Following incubation with bacterially loaded antigen-presenting cells, activation of MAIT cells and control cells can be tracked in parallel by analysis of surface markers of activation (eg, CD69) and functional responses (ie, IFN-γ release and degranulation). To determine whether MAIT cells were able to respond to pneumococci, 10 PMEN reference strains were used to probe the activation of MAIT cells in the presence of the cell line THP1. PBMCs were cultured with paraformaldehyde-fixed pneumococci and THP1 cells overnight, and interferon production by MAIT cells was examined using intracellular cytokine staining and flow cytometry (Figure 2A, B). There was clear production of IFN-γ by MAIT cells across all strains, although there was variability in the responses: production by 7 of 10 strains reached statistical significance. Similarly, CD69 expression was induced by all 10 strains, as measured by geometric mean fluorescence intensity, and reached significance for 7 strains. In comparison, there was negligible activation, as measured by IFN-γ or CD69 expression, of non-MAIT cells (ie, CD161^−^CD8^+^ T cells, which act as a negative control because they do not respond to the bacterial ligand and/or accompanying cytokine signals; Figure 2B), suggesting that pneumococci specifically activated MAIT cells.

### MAIT Cell Activation by Pneumococci in the Presence of Monocytes Is Not MR1 Dependent

**Figure 3.**
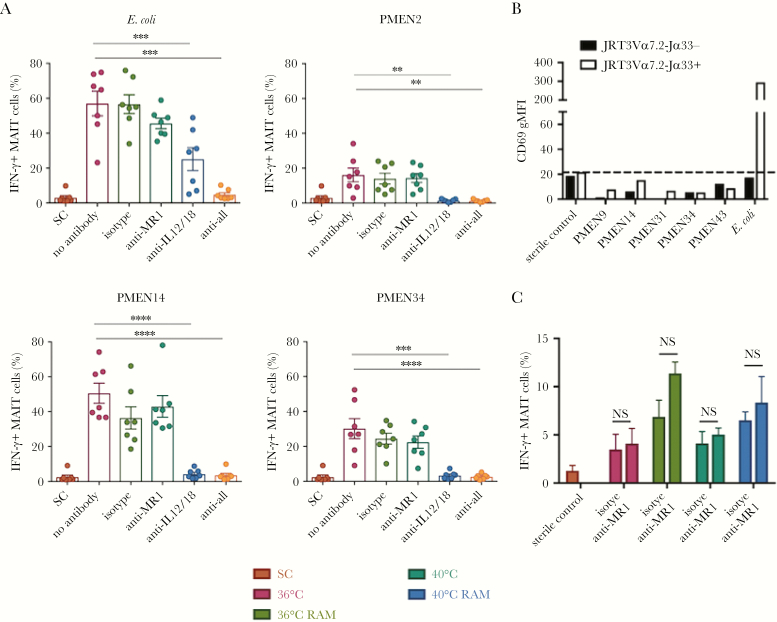
Mucosal-associated invariant T (MAIT) cell activation by pneumococci in presence of monocytes is not MR1-dependent. *A*, Paraformaldehyde-fixed Pneumococcal Molecular Epidemiological Network (PMEN) reference strains or *Escherichia coli* were cultured with peripheral blood mononuclear cells and THP1 cells in the presence of anti-MR1 blocking, anti–interleukin 12 (IL-12), and anti–interleukin 18 (IL-18) blocking antibodies. Interferon γ (IFN-γ) expression from MAIT cells is shown. ****P < .0001, ***P < .001, and **P < .01, by repeated measures 1-way analysis of variance (ANOVA) with the Dunnett multiple comparisons test, compared with the no antibody control (n = 7). *B*, Jurkat cells expressing the MAIT cell T-cell receptor (TCR; white bars) or control cells not expressing the MAIT cell TCR (black bars) were cultured with THP1 cells overnight with the indicated PMEN strains or *E. coli* as a positive control. Activation was measured as the geometric mean fluorescence intensity (gMFI) of CD69 expressed by Jurkat cells. The dotted line indicates value for the sterile control (SC). Data are representative of 3 independent experiments. *C*, The PMEN34 strain was grown overnight at 36°C or 40°C in Todd Hewitt broth with 0.5% yeast extract (THB-Y) and either cultured in THB-Y for the last 4 hours or transferred to riboflavin-free medium (RAM). The bacteria were then fixed and cultured with PBMCs and THP1 cells overnight. The frequency of IFN-γ–expressing MAIT cells is shown in the presence or absence of anti-MR1 blocking antibody. NS, nonsignificant by 2-way ANOVA with the Sidak multiple comparisons test (n = 3).

We previously found that the response of MAIT cells to *E. coli* is codependent on both MR1 and the innate cytokines IL-12 and IL-18 [14]. In these experiments, blockade of either MR1 or cytokines alone only yielded partial inhibition, while combined blockade abrogated responsiveness. To investigate the mechanism of the MAIT cell response to pneumococci, we cultured paraformaldehyde-fixed pneumococci with PBMCs and THP1 cells in the presence of anti-MR1, anti–IL-12, and anti–IL-18 blocking antibodies (Figure 3A). As expected, IFN-γ expression in response to *E. coli* was blocked significantly by anti-IL-12 and anti-IL-18 blocking antibodies, with full blocking only seen with the addition of an anti-MR1 blocking antibody (in these experiments, MR1 blockade alone had a limited effect). We found in parallel that blockade of MR1 alone had no effect on pneumococcal MAIT cell activation. Instead, in contrast to *E. coli*, blocking IL-12 and IL-18 completely abrogated MAIT cell activation across all strains tested. This suggested that, although pneumococci possess the riboflavin synthesis pathway, activation of MAIT cells by pneumococci in the presence of THP1 cells was cytokine dependent.

To confirm these findings, Jurkat cells engineered to express the MAIT cell TCR Vα7.2-Jα33 (Jurkat-MAIT cells) were cultured with fixed pneumococcal strains overnight in the presence of THP1 cells (Figure 3B). There was no significant change in the expression of CD69 by Jurkat-MAIT cells in the presence of any of the pneumococcal strains (CD69 was chosen as a robust marker for activation since the cells do not produce IFN-γ). Thus, in the presence of primary monocytes and THP1 cells, there was very little activation of MAIT cells by pneumococci through the MR1 pathway.

Given that we observed the upregulation of the riboflavin synthesis pathway in pneumococci upon heat stress (Figure 1B), we tested whether changing environmental factors such as temperature and modulating the availability of riboflavin would increase riboflavin synthesis, increase the availability of the MAIT cell ligand, and trigger activation of MAIT cells through the MR1 pathway. Pneumococcal strain PMEN34 was grown for 16 hours in THB-Y at 36°C and then transferred either to a riboflavin-containing medium and incubated at 40°C or to riboflavin-free medium and incubated at 36°C or 40°C for 4 hours, before the bacteria were fixed (Figure 3C). Although there was a slight increase in the fraction of MAIT cells expressing IFN-γ when bacteria were cultured in riboflavin-free assay medium regardless of temperature, this increase was not dependent on MR1.

We also tested whether using the live strain PMEN34 or the supernatant of pneumococcal growth culture, instead of fixed bacteria, would stimulate MAIT cells through the MR1 pathway (Supplementary Figure 1). These responses were small and could not be significantly blocked by an anti-MR1 blocking antibody; responses were similarly small when using enriched CD8^+^ T cells. Thus, in the presence of monocytes or THP1 cells, MAIT cells are activated mainly through innate cytokines rather than through MR1, regardless of temperature or riboflavin availability.

### MR1-Dependent Activation of MAIT Cells by Pneumococci in the Presence of Macrophages

**Figure 4.**
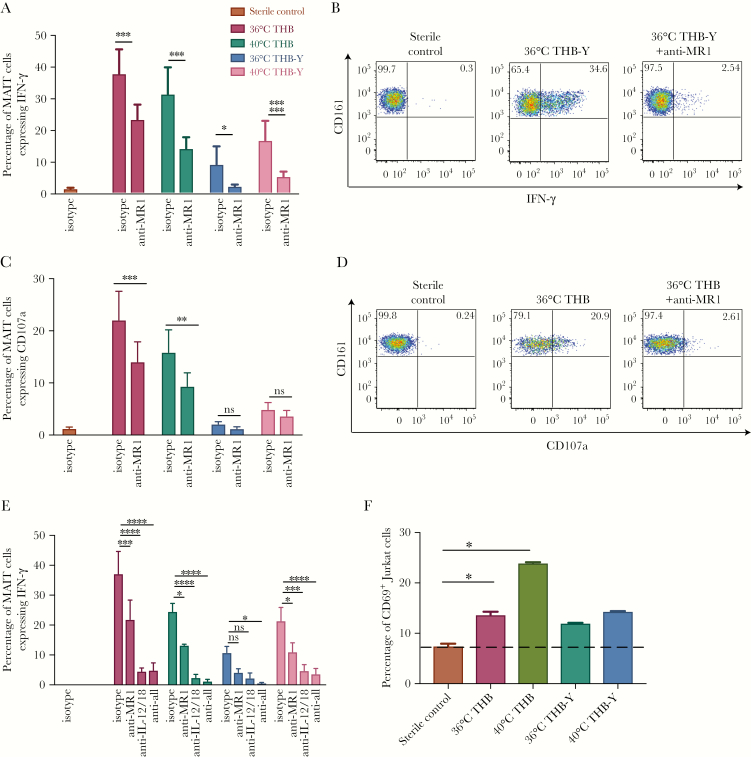
MR1-dependent activation of mucosal-associated invariant T (MAIT) cells by pneumococci in the presence of macrophages. *A*–*D*, Pneumococcal Molecular Epidemiological Network (PMEN) strain 34 was grown for 16 hours in Todd Hewitt broth with (THB) or without (THB-Y) yeast extract at either 36°C or 40°C (overnight). The bacteria were fixed and added to peripheral blood mononuclear cells (PBMCs) and monocyte-derived macrophages overnight in the presence or absence of anti-MR1 blocking antibody. The frequency of MAIT cells expressing interferon γ (IFN-γ; *A*) and representative example of IFN-γ expression from MAIT cells by fluorescence-activated cell-sorting analysis (*B*) are shown with isotype control or anti-MR1 blocking antibody. ****P < .0001, ***P < .001, and *P < .05 by 2-way analysis of variance (ANOVA) with the Sidak multiple comparisons test (n = 6). The frequency of MAIT cells expressing CD107a (*C*) and a representative example of CD107a expression from MAIT cells by fluorescence-activated cell-sorting analysis (*D*) are shown with isotype control or anti-MR1 blocking antibody. ***P < .001, **P < .01, or nonsignificant (NS) by 2-way ANOVA with the Sidak multiple comparisons test (n = 4). *E*, The PMEN34 strain was grown for 16 hours in THB-Y or THB at 36°C or 40°C. The bacteria were fixed immediately and added to PBMCs and monocyte-derived macrophages overnight in the presence or absence of indicated combinations of anti-MR1, anti–interleukin 12 (IL-12), and anti–interleukin 18 (IL-18) blocking antibodies. Frequencies of MAIT cells expressing IFN-γ are shown. ****P < .0001, ***P < .001, **P < .01, *P < .05, or NS by 2-way ANOVA with the Dunnett multiple comparisons test (n = 3). *F*, Jurkat cells expressing the MAIT cell T-cell receptor were cultured with monocyte-derived macrophages overnight with the PMEN34 strain. Activation was measured as the frequency of Jurkat cells expressing CD69. The dotted line indicates CD69 expression by Jurkat cells in the presence of sterile control. *P < .05 by repeated measures 1-way ANOVA with the Dunnett multiple comparisons test. All experiments were performed in duplicate, and data are representative of 2 independent experiments.

We next tested whether monocyte-derived macrophages can present the MR1 ligand to activate MAIT cells more effectively through MR1, because alveolar macrophages play an important role in the immune response to pneumococci [30]. Furthermore, we investigated whether temperature or the abundance of riboflavin in the medium influenced the availability of the ligand (through riboswitch-mediated modulation of the operon [31]) and therefore affected MR1-dependent activation. For this evaluation, strain PMEN34 was grown for 16 hours in THB-Y or THB at 36°C or 40°C (Figure 4A–B).

We found that when using monocyte-derived macrophages, pneumococci induced IFN-γ expression from MAIT cells that was significantly reduced by MR1 blockade. This MR1-dependent activation was seen regardless of the temperature and medium in which the pneumococci were grown. Interestingly, there was a clear increase in activation induced by bacteria grown in the basic medium, THB, as compared to bacteria grown in THB-Y (which contains additional riboflavin). This is consistent with an increase in riboflavin production in the absence of riboflavin or with induction of the operon through heat stress (or both) and, thus, is consistent with increased ligand availability.

To confirm these results, we also measured degranulation by investigating upregulation of CD107a, which is a further specific marker associated with MAIT activation. Degranulation was also induced by pneumococci grown in THB and was blocked by the anti–MR1-blocking antibody to a varying degree (Figure 4C–D).

Next, bacteria were cultured as above and added to PBMCs and macrophages overnight in the presence of anti-MR1, anti–IL-12, and anti–IL-18 antibodies (Figure 4E). There was a significant effect of blocking MR1 on IFN-γ production from MAIT cells in the presence of pneumococci cultured in THB, regardless of temperature, and full blockade in the presence of anti–IL-12 and anti–IL-18 blocking antibodies.

Finally, to confirm these results, we cultured Jurkat-MAIT cells with fixed pneumococci grown in THB or THB-Y at different temperatures in the presence of monocyte-derived macrophages (Figure 4F). Pneumococci grown in THB significantly increased the expression of CD69 in Jurkat-MAIT cells. Thus, in the presence of monocyte-derived macrophages, pneumococci were able to activate MAIT cells in an MR1-dependent manner.

### Riboflavin Operons Are Also Present in Nonpneumococcal *Streptococcus* species

**Figure 5.**
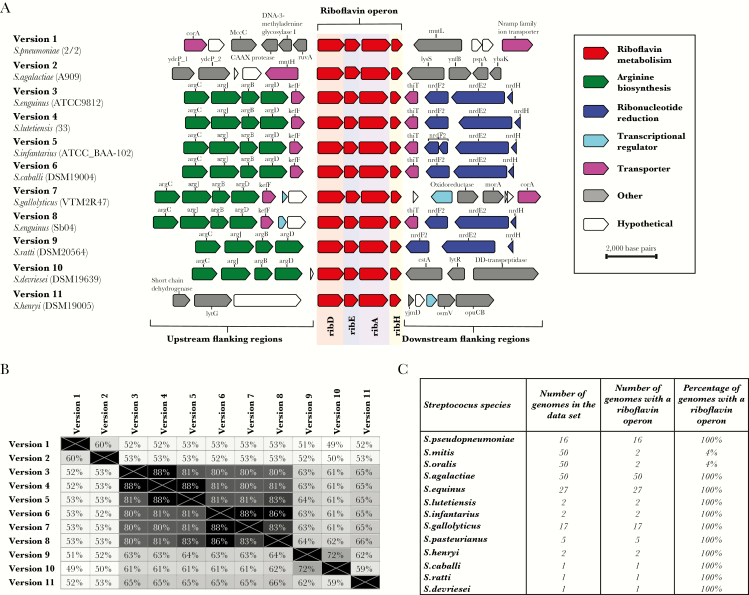

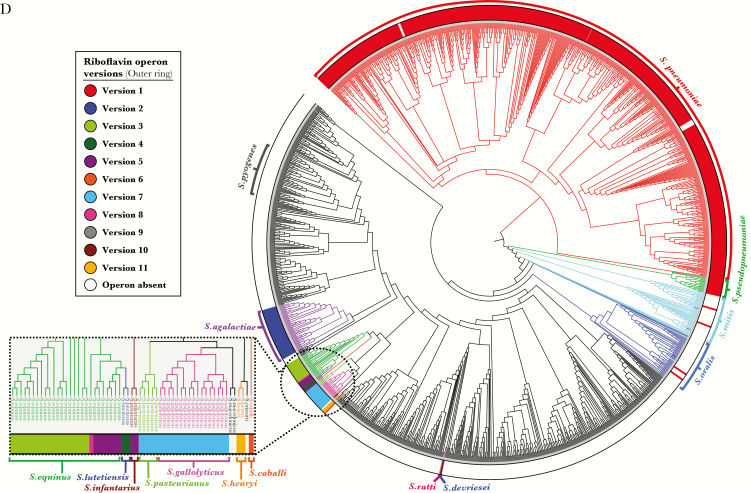
Evidence for different versions of riboflavin operons in other *Streptococcus* species. *A*, The riboflavin operon found in pneumococci (version 1) and its flanking genes are depicted and compared to 10 additional representative versions of the riboflavin operon found among other *Streptococcus* species. *B*, Matrix of pair-wise comparisons of nucleotide similarity among the 11 different versions of the riboflavin operon. *C*, Summary of the riboflavin operons found in 13 nonpneumococcal *Streptococcus* species. *D*, Phylogenetic tree constructed on the basis of the concatenated sequences of 53 ribosomal multilocus sequence type loci among 571 pneumococci and 824 *Streptococcus* species genomes. Branches of the tree were colored gray if no riboflavin operon was identified within the genome (eg, as seen for *Streptococcus pyogenes*), whereas other colors represent genomes in bacterial species that did possess a version of a riboflavin operon. The colored outer ring indicates the version of riboflavin operon that was identified in each genome or set of genomes. The rectangular box contains an expanded view of the circled area of the phylogenetic tree.

A bioinformatic investigation of 824 genomes of 69 different *Streptococcus* species revealed that the riboflavin operon was also present in other streptococci. Eleven different versions of the riboflavin operon were identified among 13 nonpneumococcal *Streptococcus* species (Supplementary Table 2). The majority of these riboflavin operons were located between genes involved in arginine biosynthesis (*argC*, *argJ*, *argB*, and *argD*) and those involved in ribonucleotide reduction (*nrdF2*, *nrdE2*, and *nrdH*; Figure 5A). Despite identical gene synteny between different versions of the riboflavin operon (Figure 5A), they differed greatly in nucleotide sequence identity (Figure 5B).

The pneumococcal version of the riboflavin operon was found among all 16 genomes of *Streptococcus pseudopneumoniae* and 2 genomes each of *Streptococcus mitis* and *Streptococcus oralis* (Figure 5C–D). No riboflavin operon genes were identified among the remaining 48 *S. mitis* and 48 *S. oralis* genomes. Pneumococci, *S. pseudopneumoniae*, *S. mitis*, and *S. oralis* are all closely related commensal streptococcal species that can exchange DNA with one another; therefore, the limited numbers of riboflavin operons present in *S. mitis* and *S. oralis* suggest that the examples identified here were the result of horizontal genetic exchange [32]. Some versions of riboflavin operons identified among other *Streptococcus* species were exclusively present in only one species. For example, version 2 was identified in all 50 genomes of *S. agalactiae* but no other species, whereas *Streptococcus equinus* contained 3 different versions of the riboflavin operon, one of which (version 5) was also found in *Streptococcus infantarius* (Figure 5D and Supplementary Table 2). Furthermore, >2400 *Streptococcus pyogenes* genomes were investigated for the presence of *Rib* genes, but the riboflavin operon could not be found (Figure 5).

## DISCUSSION

Our study is the first to demonstrate that MAIT cells can respond to and recognize pneumococci. Given the urgent global need to tackle antimicrobial-resistant strains and pneumococci not covered by the currently available vaccines, understanding the mechanism by which MAIT cells are activated by pneumococci provides a method of targeting a metabolic pathway that is highly conserved among pneumococci.

We found that MAIT cell activation by pneumococci through the MR1-restricted pathway was dependent on the type of antigen-presenting cell. There was a significant effect of blocking MR1 recognition by MAIT cells when the pneumococci were presented by macrophages but not when presented by monocytes. It has been suggested that monocytes are poor antigen presenters [33, 34]. Alveolar macrophages have been shown to be crucial for bacterial clearance in vivo [30, 31] and may be able to efficiently phagocytose the bacteria or provide more costimulatory signals. Even in the presence of macrophages, the MAIT cell response to pneumococci was highly codependent on cytokines. This is most likely due to the weak TCR signal induced by pneumococci, as seen by the significant but weak activation of Jurkat-MAIT cells even when using macrophages. The pneumococcus is a bacterium highly specialized to evade the host immune system by circumventing phagocytosis and antigen presentation, most notably by possessing a thick polysaccharide capsule, as well as through autolysis, which reduces the production of phagocyte-activating cytokines, such as tumor necrosis factor α and IL-12 [35]. The uniquely high sensitivity of MAIT cells to cytokines such as IL-12 [14] will allow these cells to boost the immune response and provide early IFN-γ production.

The pneumococcal disease with the biggest burden is pneumonia, which is the leading infectious cause of mortality in young children [1], as well as community-acquired pneumonia in elderly individuals [2]. Given that we show the mechanism by which MAIT cells respond to pneumococci and the critical role that MAIT cells play in vivo against lung infections [18, 20], it would be reasonable to suggest that MAIT cells may play a role in pneumococcal pneumonia. These cells may also be a critical factor in secondary pneumococcal pneumonia that follows influenza virus infection [36]. MAIT cells are reduced in frequency in the blood of patients with acute influenza virus infection, particularly in those who died of this disease [15, 37]. Whether the low numbers of MAIT cells in neonates [38, 39] or the decline in MAIT cell numbers in elderly individuals [40] and during influenza [15] affects the susceptibility of these patients to pneumococcal pneumonia will be important to investigate in in vivo models [41].

We used a population genomics approach to assess the prevalence and diversity of the riboflavin operon among a large and diverse collection of pneumococci. This revealed that riboflavin genes are nearly ubiquitous and highly conserved at a nucleotide level among pneumococci recovered over the past century. We also found that a number of nonpneumococcal *Streptococcus* species possess these genes, including other commensal streptococci, such as *S. agalactiae*, presenting opportunities for future studies. For example, given that MAIT cells reside in the female genital mucosa [42], it would be important to explore whether there is a MAIT cell response in the context of vaginal colonization of *S. agalactiae* among pregnant women and invasive neonatal infections [43]. Of note, not all streptococcal genomes investigated possessed a riboflavin operon (notably *S. pyogenes*, which is a major invasive pathogen), while there was also evidence of *S. mitis* and *S. oralis* acquiring the riboflavin operon through horizontal genetic exchange. Hence, caution must be exercised when extrapolating findings based on a small number of bacterial strains to the population as a whole, since they may not be representative.

Overall, these data show a robust response of MAIT cells to pneumococci and conservation of the relevant biosynthetic pathway in this organism and other closely related *Streptococcus* species. Given the low levels of MAIT cells among individuals in early life and their decline in older individuals—the highest-risk populations for invasive pneumococcal disease—further understanding of the functional role of MAIT cells in vivo in host defense against this major pathogen is of interest.

## Supplementary Data

Supplementary materials are available at *The Journal of Infectious Diseases* online. Consisting of data provided by the authors to benefit the reader, the posted materials are not copyedited and are the sole responsibility of the authors, so questions or comments should be addressed to the corresponding author.

Supplementary Table 1

Supplementary Table 2

Supplementary Figure 1

Supplementary Material

## Notes

***Acknowledgments.*** We thank Prof Regine Hakenbeck at the University of Kaiserslautern, for the stock cultures of streptococci that were newly sequenced in this study; and the High-Throughput Genomics Group at the Wellcome Trust Centre for Human Genetics (funded by the Wellcome Trust; grant 090532/Z/09/Z), for the generation of the RNA sequencing data.

***Financial support.*** This work was supported by the Wellcome Trust (grants WT109965MA [to P. K.] and 083511/Z/07/Z [to A. B. B.] and Biomedical Research Fund award 04992/Z/14/Z [to Martin J. C. Maiden, Keith A. Jolley, and A. B. B., for BIGSdb genome database support]); Cancer Research United Kingdom (grant C399/A2291 to V. C.); the National Institutes of Health (grant NIHU19AI082630 to P. K.); the National Institute for Health Research (NIHR) Senior Fellowship (to P. K.); the NIHR Biomedical Research Centre, Oxford (to P. K.); and the University of Oxford John Fell Fund (grant 123/734 to A. B. B.).

***Potential conflicts of interest.*** All authors: No reported conflicts of interest. All authors have submitted the ICMJE Form for Disclosure of Potential Conflicts of Interest. Conflicts that the editors consider relevant to the content of the manuscript have been disclosed.
