# Supplementary material for: Diverse Streptococcus pneumoniae Strains Drive a Mucosal-Associated Invariant T-Cell Response Through Major Histocompatibility Complex class I–Related Molecule–Dependent and Cytokine-Driven Pathways
Source: J Infect Dis. 2017 Dec 15;217(6):988–99. doi: 10.1093/infdis/jix647 (PMC5854017; doi:10.1093/infdis/jix647)
Supplement: Supplementary Table 1 [file jix647_suppl_supplementary_table_1.docx]

Supplementary Table 1. Descriptive data for the 571 Streptococcus pneumoniae genomes included in this study. Abbreviations: AB, Absent; AG, Assembly Gap; C, Carriage; D, Disease.

| **BIGSdb**  **id** | **Riboflavin operon version** | **Strain** | **Serotype** | **Country** | **Year** | **Carriage /Disease** | **aroE** | **gdh** | **gki** | **recP** | **spi** | **xpt** | **ddl** | **ST (MLST)** |
| --- | --- | --- | --- | --- | --- | --- | --- | --- | --- | --- | --- | --- | --- | --- |
| 1 | 1 | 1/2 | 1 | USA | 1965 | ? | 10 | 31 | 4 | 1 | 6 | 4 | 94 | 615 |
| 4 | 1 | 1/5 | 1 | Denmark | 1943 | ? | 12 | 8 | 13 | 5 | 17 | 4 | 8 | 5316 |
| 5 | 1 | 1/6 | 1 | Denmark | 1944 | ? | 12 | 8 | 13 | 5 | 17 | 4 | 8 | 5316 |
| 6 | 1 | 1/7 | 1 | Denmark | 1991 | ? | 17 | 8 | 13 | 5 | 17 | 4 | 28 | 250 |
| 10 | 1 | 3/2 | 3 | USA | 1973 | ? | 13 | 9 | 15 | 14 | 10 | 16 | 19 | 378 |
| 11 | 1 | 3/3 | 3 | USA | 1971 | ? | 13 | 9 | 15 | 14 | 311 | 16 | 19 | 7244 |
| 12 | 1 | 3/4 | 3 | Denmark | 1961 | ? | 13 | 9 | 15 | 14 | 10 | 16 | 19 | 378 |
| 15 | 1 | 3/7 | 3 | Denmark | 1961 | ? | 13 | 84 | 15 | 14 | 10 | 16 | 19 | 1377 |
| 16 | 1 | 3/8 | 3 | Denmark | 1961 | ? | 13 | 84 | 15 | 14 | 10 | 16 | 19 | 1377 |
| 18 | 1 | 4/3 | 4 | Denmark | 1962 | ? | 16 | 13 | 4 | 5 | 6 | 10 | 14 | 247 |
| 23 | 1 | 5/3 | 5 | Denmark | 1987 | ? | 16 | 12 | 9 | 1 | 41 | 33 | 33 | 289 |
| 24 | 1 | 5/4 | 5 | Singapore | 1987 | ? | 16 | 12 | 9 | 1 | 295 | 33 | 33 | 7222 |
| 28 | 1 | 7A/3 | 7A | USA | 1968 | ? | 5 | 5 | 27 | 1 | 10 | 1 | 70 | 5994 |
| 30 | 1 | 7B/3 | 7B | France | 1996 | ? | 12 | 19 | 2 | 17 | 6 | 22 | 14 | 230 |
| 31 | 1 | 7C/2 | 7C | Denmark | 1939 | ? | 1 | 8 | 4 | 1 | 9 | 3 | 18 | 7175 |
| 34 | 1 | 7F/3 | 7F | Denmark | 1962 | ? | 8 | 9 | 2 | 1 | 6 | 1 | 17 | 191 |
| 37 | 1 | 7F/6 | 7F | Sweden | 1996 | ? | 10 | 20 | 14 | 1 | 6 | 274 | 29 | 7210 |
| 38 | 1 | 8/2 | 8 | USA | 1952 | ? | 2 | 5 | 1 | 11 | 16 | 3 | 14 | 53 |
| 39 | 1 | 8/3 | 8 | Denmark | 1962 | ? | 2 | 5 | 1 | 11 | 16 | 3 | 14 | 53 |
| 44 | 1 | 9L/3 | 9L | Denmark | 1941 | ? | 7 | 334 | 8 | 8 | 6 | 28 | 14 | 7204 |
| 47 | 1 | 9N/3 | 9N | Denmark | 1938 | ? | 8 | 5 | 7 | 12 | 15 | 16 | 6 | 3983 |
| 49 | 1 | 9N/5 | 9N | Denmark | 1962 | ? | 2 | 13 | 2 | 4 | 6 | 1 | 1 | 71 |
| 64 | 1 | 12A/2 | 12A | Denmark | 1966 | ? | 1 | 2 | 7 | 18 | 17 | 14 | 17 | 5977 |
| 65 | 1 | 12A/3 | 12A | Denmark | 1966 | ? | 1 | 2 | 7 | 18 | 17 | 14 | 17 | 5977 |
| 67 | 1 | 12A/6 | 12A | Denmark | 1966 | ? | 1 | 2 | 7 | 18 | 17 | 14 | 17 | 5977 |
| 68 | 1 | 12A/7 | 12A | Denmark | 1966 | ? | 1 | 2 | 7 | 18 | 17 | 14 | 17 | 5977 |
| 69 | 1 | 12A/8 | 12A | Denmark | 1965 | ? | 1 | 2 | 7 | 18 | 17 | 14 | 17 | 5977 |
| 70 | 1 | 12A/9 | 12A | Denmark | 1966 | ? | 1 | 2 | 7 | 18 | 17 | 14 | 17 | 5977 |
| 71 | 1 | 12A/10 | 12A | Denmark | 1966 | ? | 1 | 2 | 7 | 18 | 17 | 14 | 17 | 5977 |
| 72 | 1 | 12A/11 | 12A | Denmark | 1966 | ? | 1 | 2 | 7 | 18 | 17 | 14 | 17 | 5977 |
| 73 | 1 | 12A/12 | 12A | Denmark | 1966 | ? | 1 | 2 | 7 | 18 | 17 | 14 | 17 | 5977 |
| 74 | 1 | 12A/13 | 12A | Denmark | 1966 | ? | 1 | 2 | 7 | 18 | 17 | 14 | 17 | 5977 |
| 75 | 1 | 12A/14 | 12A | Denmark | 1966 | ? | 1 | 2 | 7 | 18 | 17 | 14 | 17 | 5977 |
| 87 | 1 | 14/6 | 14 | Denmark | 1968 | ? | 2 | 13 | 2 | 8 | 17 | 79 | 288 | 7183 |
| 93 | 1 | 15B/3 | 15B/C | Denmark | 1996 | ? | 1 | 1 | 1 | 1 | 9 | 1 | 1 | 2008 |
| 100 | 1 | 17F/1 | 17F | USA | 1952 | ? | 2 | 1 | 1 | 1 | 6 | 31 | 14 | 574 |
| 106 | 1 | 18B/2 | 18B | Denmark | 1941 | ? | 7 | 2 | 1 | 1 | 10 | 1 | 6 | 4706 |
| 111 | 1 | 18F/2 | 18F | Denmark | 1945 | ? | 1 | 5 | 9 | 1 | 6 | 11 | 14 | 4863 |
| 112 | 1 | 19A/2 | 19A | Denmark | 1939 | ? | 18 | 5 | 9 | 1 | 9 | 1 | 14 | 7226 |
| 113 | 1 | 19A/3 | 19A | Denmark | 1952 | ? | 18 | 5 | 9 | 1 | 9 | 1 | 14 | 7226 |
| 117 | 1 | 19F/2 | 19F | USA | 1952 | ? | 1 | 1 | 1 | 1 | 1 | 1 | 1 | 1 |
| 119 | 1 | 19F/4 | 19F | Denmark | 1962 | ? | 18 | 5 | 4 | 1 | 15 | 1 | 6 | 7229 |
| 122 | 1 | 19F/7 | 19F | Denmark | 1952 | ? | 18 | 5 | 13 | 1 | 27 | 1 | 6 | 7230 |
| 125 | 1 | 19F/11 | 19F | Denmark | 1972 | ? | 2 | 13 | 2 | 4 | 6 | 1 | 1 | 71 |
| 126 | 1 | 19F/12 | 19F | Denmark | 1995 | ? | 5 | 5 | 7 | 7 | 8 | 5 | 4 | 87 |
| 127 | 1 | 19F/14 | 19F | Spain | 1996 | ? | 4 | 4 | 2 | 4 | 4 | 1 | 1 | 81 |
| 128 | 1 | 20/2 | 20B | USA | 1949 | ? | 15 | 8 | 8 | 1 | 15 | 1 | 31 | 7221 |
| 130 | 1 | 20/4 | 20B | Denmark | 1962 | ? | 1 | 2 | 7 | 18 | 17 | 14 | 17 | 5977 |
| 131 | 1 | 21/2 | 21 | USA | 1952 | ? | 8 | 10 | 9 | 8 | 1 | 10 | 14 | 7207 |
| 134 | 1 | 22A/3 | 22A | Denmark | 1939 | ? | 2 | 13 | 4 | 1 | 6 | 31 | 14 | 7181 |
| 138 | 1 | 23B/2 | 23B | Denmark | 1996 | ? | 18 | 13 | 8 | 6 | 3 | 6 | 8 | 1349 |
| 139 | 1 | 23F/2 | 23F | Denmark | 1940 | ? | 1 | 5 | 69 | 1 | 6 | 1 | 14 | 7170 |
| 140 | 1 | 23F/3 | 23F | Denmark | 1944 | ? | 1 | 5 | 69 | 1 | 6 | 1 | 14 | 7170 |
| 143 | 1 | 23F/6 | 23F | Denmark | 1987 | ? | 4 | 4 | 2 | 4 | 4 | 1 | 1 | 81 |
| 148 | 1 | 24F/3 | 24F | Denmark | 1943 | ? | 2 | 8 | 2 | 1 | 17 | 1 | 1 | 7179 |
| 150 | 1 | 25A/2 | 25A | Netherlands | 1986 | ? | 142 | 10 | 4 | 5 | 17 | 33 | 277 | 5407 |
| 151 | 1 | 25F/2 | 25F | USA | 1951 | ? | 1 | 15 | 87 | 134 | 10 | 1 | 6 | 5981 |
| 152 | 1 | 25F/3 | 25F | Denmark | 1962 | ? | 5 | 15 | 4 | 1 | 6 | 1 | 6 | 105 |
| 164 | 1 | 33A/3 | 33A | Denmark | 1939 | ? | 2 | 5 | 29 | 18 | 42 | 3 | 18 | 1012 |
| 168 | 1 | 33D/3 | 33D | India | 1979 | ? | 2 | 8 | 4 | 10 | 17 | 1 | 9 | 2863 |
| 170 | 1 | 34/2 | 34 | USA | 1959 | ? | 13 | 8 | 1 | 1 | 13 | 1 | 18 | 7218 |
| 171 | 1 | 34/3 | 34 | Malaysia | 1968 | ? | 7 | 16 | 19 | 16 | 6 | 19 | 14 | 7202 |
| 172 | 1 | 34/4 | 34 | Denmark | 1972 | ? | 45 | 15 | 6 | 12 | 9 | 14 | 14 | 7235 |
| 177 | 1 | 35B/4 | 35B | Denmark | 1971 | ? | 8 | 13 | 4 | 8 | 6 | 22 | 34 | 198 |
| 183 | 1 | 36/4 | 36 | Denmark | 1992 | ? | 18 | 5 | 2 | 18 | 6 | 20 | 9 | 7231 |
| 184 | 1 | 36/5 | 36 | France | 1993 | ? | 7 | 12 | 53 | 4 | 6 | 1 | 18 | 4031 |
| 186 | 1 | 37/3 | 37 | Denmark | 1943 | ? | 29 | 33 | 19 | 1 | 36 | 22 | 31 | 447 |
| 187 | 1 | 37/5 | 37 | Denmark | 1972 | ? | 29 | 33 | 19 | 1 | 36 | 22 | 31 | 447 |
| 190 | 1 | 37/8 | 37 | Denmark | 1973 | ? | 29 | 33 | 19 | 1 | 36 | 22 | 31 | 447 |
| 191 | 1 | 37/9 | 37 | Denmark | 1996 | ? | 29 | 33 | 19 | 1 | 36 | 22 | 31 | 447 |
| 196 | 1 | 41A/3 | 41A | Germany | 1972 | ? | 226 | 5 | 1 | 1 | 10 | 28 | 120 | 7238 |
| 200 | 1 | 43/4 | 43 | USA | 1954 | ? | 5 | 61 | 95 | 16 | 218 | 443 | 14 | 7193 |
| 202 | 1 | 45/2 | 45 | Denmark | 1950 | ? | 5 | 10 | 7 | 190 | 6 | 19 | 1 | 7191 |
| 203 | 1 | 45/3 | 45 | Denmark | 1949 | ? | 227 | 8 | 4 | 5 | 306 | 19 | 5 | 7239 |
| 204 | 1 | 45/4 | 45 | Denmark | 1949 | ? | 5 | 10 | 7 | 190 | 6 | 19 | 1 | 7191 |
| 209 | 1 | 48/2 | 48 | Denmark | 1962 | ? | 18 | 9 | 1 | 32 | 6 | 1 | 413 | 7233 |
| 211 | 1 | 48/4 | 48 | Denmark | 1958 | ? | 18 | 9 | 4 | 32 | 6 | 1 | 413 | 7232 |
| 212 | 1 | 48/5 | 48 | Denmark | 1957 | ? | 18 | 9 | 4 | 32 | 6 | 1 | 413 | 7232 |
| 213 | 1 | 48/6 | 48 | Denmark | 1957 | ? | 18 | 9 | 4 | 32 | 6 | 1 | 413 | 7232 |
| 216 | 1 | ICE11 | 6B | Iceland | 1998 | D | 2 | 13 | 9 | 1 | 6 | 19 | 14 | 490 |
| 217 | 1 | ICE22 | 7F | Iceland | 1993 | D | 8 | 9 | 2 | 1 | 6 | 1 | 17 | 191 |
| 218 | 1 | ICE23 | 7F | Iceland | 1993 | D | 10 | 20 | 14 | 1 | 6 | 1 | 29 | 218 |
| 219 | 1 | ICE46 | 14 | Iceland | 2003 | D | 7 | 5 | 1 | 8 | 14 | 11 | 14 | 124 |
| 225 | 1 | ICE594 | 14 | Iceland | 2005 | D | 7 | 5 | 1 | 8 | 14 | 11 | 14 | 124 |
| 226 | 1 | ICE13 | 14 | Iceland | 1998 | D | 1 | 5 | 4 | 5 | 5 | 1 | 8 | 9 |
| 228 | AB | ICE50 | 14 | Iceland | 2003 | D | 1 | 5 | 4 | 5 | 5 | 27 | 8 | 13 |
| 229 | AB | Ala243 | 14 | USA | 1998 | C | 1 | 5 | 4 | 5 | 5 | 27 | 8 | 13 |
| 232 | AB | Ala317 | 14 | USA | 2001 | D | 1 | 5 | 4 | 5 | 5 | 27 | 8 | 13 |
| 234 | 1 | Ala263 | 14 | USA | 2002 | C | 7 | 5 | 1 | 8 | 14 | 11 | 14 | 124 |
| 236 | 1 | Ala289 | 14 | USA | 2002 | D | 7 | 5 | 1 | 8 | 14 | 11 | 14 | 124 |
| 237 | 1 | Ala292 | 14 | USA | 1998 | D | 7 | 5 | 1 | 8 | 14 | 11 | 14 | 124 |
| 240 | 1 | USA2 | 18C | USA | 1999 | D | 7 | 2 | 1 | 1 | 10 | 1 | 21 | 113 |
| 244 | 1 | USA6 | 14 | USA | 1999 | D | 7 | 5 | 1 | 8 | 14 | 11 | 14 | 124 |
| 246 | 1 | USA8 | 9N | USA | 2001 | D | 2 | 8 | 2 | 4 | 6 | 492 | 1 | 8119 |
| 247 | 1 | USA9 | 14 | Brazil | 1999 | D | 2 | 8 | 2 | 4 | 6 | 1 | 1 | 66 |
| 249 | 1 | USA11 | 14 | Brazil | 2007 | D | 2 | 8 | 2 | 4 | 6 | 1 | 1 | 66 |
| 250 | 1 | USA12 | 23F | Poland | 2001 | C | 2 | 8 | 2 | 4 | 6 | 1 | 1 | 66 |
| 251 | 1 | USA13 | 19F | Germany | 2005 | ? | 2 | 8 | 2 | 4 | 6 | 1 | 1 | 66 |
| 254 | 1 | USA16 | 7F | Brazil | 2003 | D | 8 | 9 | 2 | 1 | 6 | 1 | 17 | 191 |
| 256 | 1 | USA18 | 12F | USA | 1999 | D | 10 | 20 | 14 | 1 | 6 | 1 | 29 | 218 |
| 258 | 1 | USA20 | 7F | South Africa | 1999 | D | 10 | 20 | 14 | 1 | 6 | 1 | 29 | 218 |
| 260 | 1 | USA22 | 6C | USA | 2005 | D | 2 | 13 | 9 | 1 | 6 | 19 | 14 | 490 |
| 261 | 1 | Spain23F-1 | 23F | Spain | 1984 | D | 4 | 4 | 2 | 4 | 4 | 1 | 1 | 81 |
| 262 | 1 | Spain6B-2 | 6Bii | Spain | 1988 | D | 5 | 6 | 1 | 2 | 6 | 3 | 4 | 90 |
| 263 | 1 | Spain9V-3 | 9V | France | 1993 | D | 7 | 11 | 10 | 1 | 6 | 8 | 1 | 156 |
| 264 | 1 | Tennessee23F-4 | 23F | USA | 1991 | D | 1 | 8 | 6 | 2 | 6 | 4 | 6 | 37 |
| 265 | 1 | Spain14-5 | 14 | Spain | 1990 | D | 1 | 5 | 4 | 11 | 9 | 3 | 16 | 18 |
| 266 | 1 | Hungary19A-6 | 19A | Hungary | 1989 | ? | 7 | 13 | 42 | 6 | 10 | 6 | 56 | 268 |
| 268 | 1 | S.Africa6B-8 | 6B | South Africa | 1990 | D | 7 | 22 | 1 | 2 | 5 | 1 | 14 | 185 |
| 269 | 1 | England14-9 | 14 | United Kingdom | 1994 | D | 1 | 5 | 4 | 5 | 5 | 1 | 8 | 9 |
| 270 | 1 | CSR14-10 | 14 | Czech Republic | 1987 | D | 1 | 5 | 4 | 1 | 5 | 3 | 3 | 20 |
| 272 | 1 | Finland6B-12 | 6Bii | Finland | 1987 | D | 5 | 6 | 1 | 2 | 6 | 1 | 28 | 238 |
| 273 | 1 | S.Africa19A-13 | 19A | South Africa | 1988 | D | 1 | 8 | 9 | 5 | 11 | 1 | 12 | 41 |
| 274 | 1 | Taiwan19F-14 | 19F | Taiwan | 1997 | D | 15 | 16 | 19 | 15 | 6 | 20 | 26 | 236 |
| 275 | 1 | Taiwan23F-15 | 23F | Taiwan | 1997 | D | 15 | 29 | 4 | 21 | 30 | 1 | 14 | 242 |
| 277 | 1 | Maryland6B-17 | 6B | USA | 1997 | D | 7 | 6 | 9 | 2 | 6 | 1 | 67 | 384 |
| 278 | 1 | Tennessee14-18 | 14 | USA | 1997 | D | 2 | 8 | 7 | 4 | 6 | 1 | 1 | 67 |
| 282 | 1 | Greece6B-22 | 6Bii | Greece | 1996 | C | 5 | 6 | 1 | 2 | 6 | 1 | 14 | 273 |
| 294 | 1 | Denmark12F-34 | 12F | Canada | 1995 | D | 10 | 20 | 14 | 1 | 6 | 1 | 29 | 218 |
| 295 | 1 | Netherlands14-35 | 14 | Netherlands | 1980 | D | 7 | 5 | 1 | 8 | 14 | 11 | 14 | 124 |
| 296 | 1 | Netherlands18C-36 | 18C | Netherlands | 1980 | D | 7 | 2 | 1 | 1 | 10 | 1 | 21 | 113 |
| 299 | 1 | Netherlands7F-39 | 7F | Netherlands | 1984 | D | 8 | 9 | 2 | 1 | 6 | 1 | 17 | 191 |
| 304 | 1 | CDC0288-04 | 12F | USA | 2004 | D | 10 | 20 | 14 | 1 | 9 | 1 | 29 | 220 |
| 305 | 1 | CDC1087-00 | 7F | USA | 1999 | D | 8 | 9 | 2 | 1 | 6 | 1 | 17 | 191 |
| 306 | 1 | CDC1873-00 | 6A | USA | 1999 | D | 6 | 11 | 1 | 1 | 15 | 72 | 77 | 376 |
| 307 | 1 | CDC3059-06 | 19A | USA | 2005 | D | 8 | 13 | 14 | 4 | 17 | 4 | 14 | 199 |
| 308 | 1 | CGSP14 | 14 | Taiwan | 2005 | D | 1 | 5 | 4 | 5 | 5 | 3 | 8 | 15 |
| 309 | 1 | D39 | 2 | USA | 1916 | D | 7 | 5 | 1 | 1 | 10 | 7 | 15 | 595 |
| 310 | 1 | SA84 | 14 | South Africa | ? | ? | 13 | 5 | 4 | 5 | 5 | 3 | 8 | 5174 |
| 311 | 1 | MLV-016 | 11A | ? | ? | C | 2 | 5 | 29 | 12 | 16 | 3 | 14 | 62 |
| 312 | 1 | R6 | NT | ? | 1964 | ? | 7 | 5 | 1 | 1 | 10 | 7 | 15 | 595 |
| 313 | 1 | 670 | 6Bii | Spain | 1988 | D | 5 | 6 | 1 | 2 | 6 | 3 | 4 | 90 |
| 314 | 1 | SA27 | 14 | South Africa | ? | ? | 13 | 5 | 4 | 5 | 5 | 3 | 8 | 5174 |
| 315 | 1 | SPnINV104B | 1 | United Kingdom | 1998 | D | 12 | 5 | 13 | 5 | 17 | 4 | 20 | 227 |
| 316 | 1 | SPnINV200 | 14 | United Kingdom | 1995 | D | 1 | 5 | 4 | 5 | 5 | 1 | 8 | 9 |
| 317 | 1 | SPnOXC141 | 3 | United Kingdom | 2001 | C | 7 | 15 | 2 | 10 | 6 | 1 | 22 | 180 |
| 318 | 1 | TIGR4 | 4 | Norway | 1991 | D | 10 | 5 | 4 | 5 | 13 | 10 | 18 | 205 |
| 319 | 1 | P1031 | 1 | Ghana | 2002 | D | 10 | 5 | 4 | 1 | 7 | 19 | 9 | 303 |
| 320 | 1 | 70585 | 5 | Bangladesh | ? | D | 16 | 12 | 9 | 1 | 41 | 33 | 33 | 289 |
| 321 | 1 | JJA | 14 | Brazil | 1995 | D | 2 | 8 | 2 | 4 | 6 | 1 | 1 | 66 |
| 399 | 1 | SMRU1390 | NT | Thailand | 2009 | ? | 7 | 8 | 453 | 16 | 16 | 14 | 14 | 10402 |
| 401 | 1 | SMRU2128 | 15A | Thailand | 2009 | ? | 7 | 443 | 2 | 16 | 15 | 1 | 17 | 10423 |
| 430 | 1 | UoS3138 | 33B | United Kingdom | ? | ? | 7 | 5 | 15 | 10 | 17 | 19 | 255 | 4697 |
| 966 | 1 | USA23 | 23F | USA | 1989 | D | 1 | 8 | 1 | 2 | 6 | 4 | 6 | 33 |
| 967 | 1 | USA24 | 23F | South Africa | 1989 | ? | 4 | 4 | 2 | 4 | 4 | 1 | 1 | 81 |
| 969 | 1 | USA26 | 23F | USA | 1985 | D | 4 | 4 | 2 | 4 | 4 | 1 | 1 | 81 |
| 970 | 1 | USA27 | 23F | USA | 1985 | ? | 1 | 5 | 9 | 2 | 6 | 4 | 14 | 1906 |
| 971 | 1 | USA28 | 23F | USA | 1988 | ? | 4 | 4 | 2 | 4 | 4 | 1 | 1 | 81 |
| 974 | 1 | USA31 | 23F | USA | 1983 | ? | 61 | 53 | 122 | 16 | 6 | 38 | 14 | 7256 |
| 976 | 1 | USA33 | 6Bii | USA | 1981 | D | 5 | 6 | 1 | 2 | 6 | 1 | 28 | 238 |
| 977 | 1 | USA34 | 19A | USA | 1985 | ? | 1 | 8 | 9 | 5 | 11 | 1 | 12 | 41 |
| 978 | 1 | USA35 | 23F | USA | 1989 | D | 4 | 4 | 2 | 4 | 4 | 1 | 1 | 81 |
| 982 | 1 | USA39 | 6Bii | USA | 1990 | D | 5 | 6 | 1 | 2 | 6 | 3 | 4 | 90 |
| 985 | 1 | USA42 | 23F | USA | 1984 | ? | 4 | 4 | 2 | 4 | 4 | 1 | 1 | 81 |
| 990 | 1 | USA47 | 23F | USA | 1985 | D | 4 | 4 | 2 | 4 | 4 | 1 | 1 | 81 |
| 991 | 1 | USA48 | 23F | USA | 1991 | D | 1 | 8 | 6 | 2 | 6 | 4 | 6 | 37 |
| 993 | 1 | SA1 | 19A | South Africa | 1978 | C | 1 | 8 | 9 | 5 | 11 | 1 | 12 | 41 |
| 994 | 1 | SA2 | 19A | South Africa | 1978 | C | 1 | 8 | 9 | 5 | 11 | 1 | 12 | 41 |
| 995 | 1 | SA3 | 19A | South Africa | 1978 | C | 1 | 8 | 9 | 5 | 11 | 1 | 12 | 41 |
| 996 | 1 | SA4 | 19A | South Africa | 1978 | C | 1 | 8 | 9 | 5 | 11 | 1 | 12 | 41 |
| 997 | 1 | SA5 | 19A | South Africa | 1978 | C | 1 | 8 | 9 | 5 | 11 | 1 | 12 | 41 |
| 998 | 1 | SA6 | 19A | South Africa | 1978 | C | 1 | 8 | 9 | 5 | 11 | 1 | 12 | 41 |
| 999 | 1 | SA7 | 19A | South Africa | 1978 | C | 1 | 8 | 9 | 5 | 11 | 1 | 165 | 1605 |
| 1000 | 1 | SA8 | 19A | South Africa | 1978 | C | 1 | 8 | 9 | 5 | 11 | 1 | 165 | 1605 |
| 1001 | 1 | SA9 | 19A | South Africa | 1978 | C | 1 | 8 | 9 | 5 | 11 | 1 | 12 | 41 |
| 1002 | 1 | SA10 | 19A | South Africa | 1977 | D | 2 | 8 | 9 | 5 | 11 | 1 | 13 | 1656 |
| 1003 | 1 | SA11 | 6A | South Africa | 1978 | C | 6 | 62 | 5 | 30 | 88 | 8 | 6 | 1094 |
| 1004 | 1 | SA12 | 6A | South Africa | 1978 | C | 6 | 62 | 5 | 30 | 88 | 8 | 6 | 1094 |
| 1005 | 1 | SA13 | 6A | South Africa | 1978 | C | 6 | 62 | 5 | 30 | 88 | 8 | 6 | 1094 |
| 1006 | 1 | SA14 | 6A | South Africa | 1978 | C | 6 | 62 | 5 | 30 | 88 | 8 | 6 | 1094 |
| 1007 | 1 | SA15 | 6A | South Africa | 1978 | C | 6 | 62 | 5 | 30 | 88 | 8 | 6 | 1094 |
| 1008 | 1 | SA16 | 6A | South Africa | 1978 | C | 6 | 62 | 5 | 30 | 88 | 8 | 6 | 1094 |
| 1009 | 1 | SA17 | 6A | South Africa | 1978 | C | 6 | 62 | 5 | 30 | 88 | 8 | 6 | 1094 |
| 1010 | 1 | SA18 | 6A | South Africa | 1978 | C | 6 | 62 | 5 | 30 | 88 | 8 | 6 | 1094 |
| 1011 | 1 | SA19 | 6A | South Africa | 1978 | C | 6 | 62 | 5 | 30 | 88 | 8 | 6 | 1094 |
| 1012 | 1 | SA20 | 6A | South Africa | 1978 | C | 6 | 62 | 5 | 30 | 88 | 8 | 6 | 1094 |
| 1013 | 1 | SA21 | 6A | South Africa | 1978 | C | 6 | 62 | 5 | 30 | 88 | 149 | 6 | 1607 |
| 1014 | 1 | SA22 | 14 | South Africa | 1978 | C | 7 | 5 | 1 | 8 | 14 | 11 | 14 | 124 |
| 1015 | 1 | SA23 | 19A | South Africa | 1978 | C | 7 | 13 | 8 | 6 | 25 | 6 | 8 | 172 |
| 1016 | 1 | SA24 | 19A | South Africa | 1978 | C | 10 | 11 | 4 | 1 | 6 | 149 | 5 | 1608 |
| 1017 | 1 | SA25 | 19A | South Africa | 1978 | C | 10 | 11 | 4 | 1 | 6 | 149 | 5 | 1608 |
| 1018 | 1 | SA26 | 19A | South Africa | 1978 | C | 10 | 99 | 4 | 1 | 6 | 149 | 5 | 1610 |
| 1028 | 1 | SA28 | 14 | South Africa | 1978 | D | 13 | 5 | 4 | 5 | 5 | 3 | 8 | 5174 |
| 1031 | 1 | SA31 | 6A | South Africa | 1978 | D | 6 | 62 | 5 | 30 | 88 | 8 | 6 | 1094 |
| 1032 | 1 | SA32 | 6A | South Africa | 1978 | D | 6 | 62 | 5 | 30 | 88 | 8 | 6 | 1094 |
| 1034 | 1 | SA34 | 29 | South Africa | 1988 | D | 18 | 5 | 2 | 5 | 27 | 19 | 9 | 5178 |
| 1035 | 1 | SA35 | 29 | South Africa | 1987 | D | 18 | 5 | 2 | 5 | 27 | 19 | 9 | 5178 |
| 1036 | 1 | SA36 | 29 | South Africa | 1987 | D | 18 | 5 | 2 | 5 | 27 | 19 | 9 | 5178 |
| 1037 | 1 | SA37 | 19F | South Africa | 1987 | D | 12 | 62 | 9 | 10 | 3 | 20 | 6 | 5179 |
| 1038 | 1 | SA38 | 6A | South Africa | 1987 | D | 6 | 62 | 5 | 30 | 88 | 8 | 6 | 1094 |
| 1039 | 1 | SA39 | 19A | South Africa | 1987 | D | 1 | 8 | 9 | 5 | 11 | 1 | 12 | 41 |
| 1041 | 1 | SA41 | 6Bii | South Africa | ? | D | 6 | 62 | 5 | 30 | 88 | 8 | 6 | 1094 |
| 1042 | 1 | SA42 | 19F | South Africa | ? | D | 15 | 16 | 19 | 5 | 6 | 20 | 26 | 763 |
| 1043 | 1 | SA43 | 19A | South Africa | ? | D | 1 | 8 | 9 | 5 | 11 | 1 | 12 | 41 |
| 1044 | 1 | SA44 | 6A | South Africa | ? | D | 6 | 62 | 5 | 30 | 88 | 8 | 6 | 1094 |
| 1045 | 1 | SA45 | 6A | South Africa | ? | D | 6 | 62 | 5 | 30 | 88 | 8 | 6 | 1094 |
| 1056 | 1 | PN1 | 6A | PNG | 1972 | D | 7 | 13 | 8 | 6 | 6 | 6 | 494 | 7151 |
| 1057 | 1 | PN2 | 4 | PNG | 1969 | D | 1 | 13 | 4 | 5 | 6 | 214 | 495 | 7152 |
| 1058 | 1 | SA47 | 19A | South Africa | 1979 | D | 1 | 8 | 9 | 5 | 11 | 1 | 165 | 1605 |
| 1059 | 1 | SA48 | 19A | South Africa | 1979 | D | 2 | 8 | 9 | 5 | 11 | 1 | 13 | 1656 |
| 1060 | 1 | SA49 | 19A | South Africa | 1982 | ? | 58 | 13 | 54 | 1 | 241 | 1 | 18 | 7255 |
| 1061 | 1 | SA51 | 6B | South Africa | 1989 | D | 7 | 22 | 1 | 2 | 5 | 1 | 14 | 185 |
| 1062 | 1 | SA52 | 6B | South Africa | 1984 | D | 2 | 62 | 1 | 25 | 6 | 20 | 5 | 2421 |
| 1063 | 1 | SA53 | 11A | South Africa | 1983 | D | 1 | 6 | 29 | 10 | 6 | 79 | 18 | 7247 |
| 1064 | 1 | SA54 | 6B | South Africa | 1983 | ? | 2 | 5 | 1 | 2 | 292 | 220 | 27 | 7084 |
| 1065 | 1 | SA55 | 19A | South Africa | 1983 | ? | 2 | 13 | 8 | 25 | 25 | 6 | 8 | 75 |
| 1066 | 1 | SA56 | 6Bii | South Africa | 1984 | D | 6 | 62 | 5 | 30 | 88 | 8 | 6 | 1094 |
| 1067 | 1 | SA57 | 6B | South Africa | 1984 | ? | 7 | 22 | 1 | 2 | 5 | 1 | 14 | 185 |
| 1068 | 1 | SA58 | 19F | South Africa | 1986 | ? | 2 | 8 | 111 | 5 | 17 | 1 | 6 | 7249 |
| 1069 | 1 | SA59 | 6A | South Africa | 1985 | D | 6 | 62 | 1 | 30 | 88 | 8 | 6 | 7251 |
| 1070 | 1 | SA60 | 19A | South Africa | 1985 | D | 7 | 13 | 8 | 6 | 25 | 6 | 8 | 172 |
| 1071 | 1 | SA61 | 19A | South Africa | 1985 | D | 7 | 5 | 8 | 6 | 25 | 6 | 8 | 5183 |
| 1072 | 1 | SA62 | 6Bii | South Africa | 1985 | ? | 6 | 62 | 5 | 30 | 88 | 8 | 6 | 1094 |
| 1073 | 1 | SA63 | 14 | South Africa | 1985 | ? | 7 | 5 | 1 | 8 | 14 | 11 | 14 | 124 |
| 1074 | 1 | SA64 | 19F | South Africa | 1985 | D | 54 | 8 | 4 | 5 | 9 | 1 | 18 | 7254 |
| 1076 | 1 | SA66 | 6B | South Africa | 1985 | D | 7 | 22 | 1 | 2 | 5 | 1 | 14 | 185 |
| 1077 | 1 | SA67 | 6B | South Africa | 1985 | ? | 2 | 62 | 1 | 2 | 6 | 14 | 5 | 7250 |
| 1078 | 1 | SA68 | 6A | South Africa | 1985 | ? | 228 | 62 | 5 | 30 | 88 | 8 | 6 | 7257 |
| 1079 | 1 | SA69 | 19A | South Africa | 1985 | ? | 1 | 8 | 9 | 5 | 11 | 149 | 6 | 7248 |
| 1080 | 1 | SA70 | 19A | South Africa | 1985 | D | 7 | 5 | 8 | 6 | 25 | 6 | 8 | 5183 |
| 1081 | 1 | SA71 | 23F | South Africa | 1989 | ? | 4 | 4 | 2 | 4 | 4 | 8 | 1 | 6215 |
| 1082 | 1 | SA72 | 6A | South Africa | 1988 | ? | 6 | 62 | 5 | 30 | 88 | 8 | 6 | 1094 |
| 1083 | 1 | SA73 | 19A | South Africa | 1988 | ? | 1 | 8 | 9 | 5 | 11 | 1 | 12 | 41 |
| 1084 | 1 | SA74 | 23F | South Africa | 1988 | ? | 4 | 4 | 2 | 4 | 4 | 8 | 1 | 6215 |
| 1085 | 1 | SA75 | 23F | South Africa | 1987 | ? | 4 | 4 | 2 | 4 | 4 | 8 | 1 | 6215 |
| 1086 | 1 | SA76 | 14 | South Africa | 1988 | ? | 1 | 5 | 4 | 30 | 27 | 20 | 364 | 9682 |
| 1087 | 1 | SA77 | 23F | South Africa | 1987 | ? | 4 | 4 | 2 | 4 | 4 | 8 | 1 | 6215 |
| 1088 | 1 | SA78 | 14 | South Africa | 1989 | D | 11 | 5 | 4 | 8 | 14 | 11 | 499 | 7253 |
| 1089 | 1 | SA79 | 19A | South Africa | 1989 | D | 7 | 13 | 8 | 6 | 25 | 6 | 8 | 172 |
| 1090 | 1 | SA80 | 23F | South Africa | 1989 | ? | 4 | 4 | 2 | 4 | 4 | 1 | 1 | 81 |
| 1091 | 1 | SA81 | 23F | South Africa | 1989 | ? | 4 | 4 | 2 | 4 | 4 | 8 | 1 | 6215 |
| 1092 | 1 | SA82 | 14 | South Africa | 1989 | D | 7 | 5 | 4 | 8 | 14 | 11 | 611 | 9683 |
| 1093 | 1 | SA83 | 6B | South Africa | 1989 | D | 2 | 5 | 1 | 11 | 16 | 3 | 14 | 53 |
| 1094 | 1 | TCH8431 | 19A | ? | ? | ? | 4 | 16 | 19 | 15 | 6 | 20 | 1 | 320 |
| 1095 | AB | BS397 | NT | USA | 2000 | D | 1 | 5 | 4 | 5 | 5 | 27 | 8 | 13 |
| 1096 | 1 | BS455 | NT | USA | 1999 | D | 1 | 5 | 4 | 5 | 5 | 3 | 101 | 2011 |
| 1097 | AB | BS457 | NT | USA | 2000 | D | 1 | 5 | 4 | 5 | 5 | 27 | 8 | 13 |
| 1098 | AB | BS458 | 14 | USA | 2000 | D | 1 | 5 | 4 | 5 | 5 | 27 | 8 | 13 |
| 1099 | 1 | CCRI1974 | 14 | Canada | ? | D | 7 | 5 | 1 | 8 | 14 | 11 | 14 | 124 |
| 1101 | 1 | SP11-BS70 | 11A | USA | ? | D | 2 | 5 | 29 | 12 | 16 | 3 | 14 | 62 |
| 1102 | AB | SP14-BS292 | 14 | USA | 1999 | ? | 1 | 5 | 4 | 5 | 5 | 27 | 8 | 13 |
| 1103 | 1 | SP14-BS69 | 14 | USA | ? | D | 7 | 5 | 1 | 8 | 14 | 11 | 14 | 124 |
| 1104 | 1 | SP18-BS74 | 6Bii | USA | ? | D | 7 | 6 | 1 | 2 | 6 | 15 | 436 | 6214 |
| 1105 | 1 | SP19-BS75 | 19F | USA | ? | D | 1 | 5 | 1 | 1 | 1 | 1 | 8 | 485 |
| 1106 | 1 | SP23-BS72 | 23F | USA | ? | D | 1 | 8 | 6 | 2 | 6 | 4 | 6 | 37 |
| 1107 | 1 | SP3-BS71 | 3 | USA | ? | D | 7 | 15 | 2 | 10 | 6 | 1 | 22 | 180 |
| 1108 | 1 | SP6-BS73 | 6A | USA | ? | D | 5 | 7 | 4 | 10 | 10 | 1 | 27 | 460 |
| 1109 | 1 | SP9-BS68 | 9V | USA | ? | D | 7 | 11 | 10 | 1 | 6 | 76 | 14 | 1269 |
| 1110 | 1 | Sp03-4156 | 3 | Netherlands | 2003 | C | 7 | 15 | 2 | 10 | 6 | 1 | 22 | 180 |
| 1111 | 1 | Sp03-4183 | 3 | Netherlands | 2003 | C | 7 | 15 | 2 | 10 | 6 | 1 | 22 | 180 |
| 1112 | 1 | Sp99_4038 | 3 | United Kingdom | 1999 | D | 7 | 15 | 2 | 10 | 6 | 1 | 22 | 180 |
| 1113 | 1 | Sp99_4039 | 3 | United Kingdom | 1999 | D | 7 | 15 | 2 | 10 | 6 | 1 | 22 | 180 |
| 1114 | 1 | Sp03_2672 | 1 | United Kingdom | ? | D | 12 | 8 | 13 | 5 | 16 | 4 | 20 | 306 |
| 1115 | 1 | Sp03-3038 | 1 | United Kingdom | ? | D | 12 | 8 | 13 | 5 | 16 | 4 | 20 | 306 |
| 1116 | 1 | AP200 | 11A | Italy | 2003 | D | 2 | 5 | 29 | 12 | 16 | 3 | 14 | 62 |
| 1117 | 1 | G54 | 19F | Italy | ? | D | ? | 5 | 36 | 12 | 17 | 21 | 14 | ? |
| 1118 | 1 | Canada MDR_19A | 19A | Canada | 2007 | D | 4 | 16 | 19 | 15 | 6 | 20 | 1 | 320 |
| 1119 | 1 | Canada MDR_19F | 19F | Canada | 2001 | D | 4 | 16 | 19 | 15 | 6 | 20 | 1 | 320 |
| 1120 | 1 | GA04375 | 19F | USA | 1995 | D | 15 | 16 | 19 | 15 | 6 | 20 | 26 | 236 |
| 1121 | 1 | GA07228 | 3 | USA | 1997 | D | 7 | 15 | 2 | 10 | 6 | 1 | 22 | 180 |
| 1122 | AB | GA07643 | 4 | USA | 1998 | D | 16 | 13 | 4 | 4 | 6 | 113 | 18 | 695 |
| 1123 | 1 | GA08780 | 9V | USA | 1997 | D | 7 | 11 | 10 | 1 | 6 | 8 | 1 | 156 |
| 1124 | 1 | GA11184 | 19F | USA | 1999 | D | 8 | 13 | 1 | 4 | 6 | 4 | 14 | 649 |
| 1125 | 1 | GA11304 | 6Bii | USA | 1999 | D | 5 | 6 | 1 | 2 | 6 | 3 | 4 | 90 |
| 1126 | AG | GA11426 | 19A | USA | 1999 | D | 8 | 13 | 14 | 4 | 17 | 4 | 207 | 1936 |
| 1127 | 1 | GA11663 | 19F | USA | 1999 | D | 4 | 4 | 2 | 4 | 4 | 1 | 1 | 81 |
| 1128 | AB | GA13338 | 14 | USA | 1999 | D | 1 | 5 | 4 | 5 | 5 | 27 | 8 | 13 |
| 1129 | 1 | GA13455 | 19F | USA | 1999 | D | 15 | 5 | 19 | 15 | 6 | 20 | 26 | 651 |
| 1130 | 1 | GA13494 | 14 | USA | 1999 | D | 41 | 5 | 1 | 8 | 14 | 11 | 14 | 656 |
| 1131 | 1 | GA13637 | 18C | USA | 1999 | D | 7 | 46 | 1 | 1 | 10 | 4 | 21 | 3060 |
| 1132 | 1 | GA13856 | 33F | USA | 1999 | D | 5 | 35 | 40 | 12 | 9 | 39 | 18 | 636 |
| 1133 | 1 | GA14798 | 19F | USA | 2000 | D | 4 | 16 | 19 | 15 | 6 | 20 | 189 | 3039 |
| 1134 | 1 | GA16121 | 19F | USA | 2000 | D | 15 | 5 | 19 | 15 | 6 | 20 | 4 | 1461 |
| 1135 | 1 | GA16242 | 6Bii | USA | 2001 | D | 7 | 6 | 1 | 17 | 6 | 1 | 104 | 1536 |
| 1136 | 1 | GA16531 | 6Bii | USA | 2001 | D | 7 | 6 | 1 | 2 | 6 | 15 | 14 | 146 |
| 1137 | 1 | GA16833 | 19F | USA | 2002 | D | ? | 16 | 19 | 15 | ? | 20 | 104 | ? |
| 1138 | 1 | GA17227 | 23F | USA | 2000 | D | 15 | 29 | 4 | 21 | 30 | 1 | 14 | 242 |
| 1139 | 1 | GA17371 | 19F | USA | 2000 | D | 15 | 5 | 19 | 15 | 6 | 20 | 1 | 8014 |
| 1140 | 1 | GA17328 | 6A | USA | 2000 | D | 6 | 11 | 1 | 1 | 15 | 72 | 77 | 376 |
| 1141 | 1 | GA17570 | 9V | USA | 2001 | D | 7 | 11 | 10 | 1 | 6 | 8 | 1 | 156 |
| 1142 | 1 | GA17971 | 6A | USA | 2001 | D | 2 | 5 | 90 | 61 | 17 | 130 | 6 | 1175 |
| 1143 | 1 | GA18523 | 19A | USA | 2001 | D | 4 | 16 | 19 | 68 | 6 | 20 | 26 | 2476 |
| 1144 | 1 | GA19077 | 6A | USA | 2002 | D | 6 | 11 | 1 | 67 | 15 | 72 | 152 | 1296 |
| 1145 | 1 | GA19451 | 19F | USA | 2003 | D | 4 | 16 | 19 | 15 | 6 | 20 | 26 | 271 |
| 1146 | 1 | GA19690 | 3 | USA | 2004 | D | 7 | 15 | 2 | 10 | 6 | 1 | 22 | 180 |
| 1147 | 1 | GA41277 | 19A | USA | 2004 | D | 8 | 13 | 14 | 4 | 17 | 4 | 14 | 199 |
| 1148 | 1 | GA41301 | 23F | USA | 2004 | D | 15 | 29 | 4 | 21 | 30 | 1 | 14 | 242 |
| 1149 | 1 | GA41317 | 33F | USA | 2004 | D | 5 | 12 | 29 | 16 | 9 | 39 | 1 | 2705 |
| 1150 | 1 | GA41410 | 19A | USA | 2004 | D | 6 | 11 | 1 | 67 | ? | ? | 14 | ? |
| 1151 | 1 | GA41437 | 6A | USA | 2004 | D | 6 | 11 | 1 | 1 | 15 | 72 | 77 | 376 |
| 1152 | 1 | GA41538 | 6Bii | USA | 2004 | D | 7 | 6 | 9 | 2 | 6 | 1 | 67 | 384 |
| 1153 | 1 | GA41565 | 19A | USA | 2004 | D | 4 | 4 | 2 | 4 | 4 | 1 | 1 | 81 |
| 1154 | AB | GA41688 | 14 | USA | 2004 | D | 1 | 5 | 4 | 5 | 5 | 27 | 8 | 13 |
| 1155 | 1 | GA43265 | 19A | USA | 2005 | D | 8 | 13 | 14 | 4 | 17 | 4 | 244 | 2584 |
| 1156 | 1 | GA43380 | 19A | USA | 2005 | D | 2 | 13 | 4 | 11 | ? | 16 | 1 | ? |
| 1157 | 1 | GA44194 | 19A | USA | 2005 | D | 2 | 5 | 36 | 12 | 15 | 21 | 14 | 2543 |
| 1158 | 1 | GA44288 | 19A | USA | 2005 | D | 4 | 16 | 19 | 15 | 6 | 20 | 1 | 320 |
| 1159 | 1 | GA44378 | 23F | USA | 2005 | D | 4 | 4 | 2 | 4 | 4 | 1 | 1 | 81 |
| 1160 | 1 | GA44452 | 19A | USA | 2005 | D | 4 | 11 | 1 | 67 | 15 | 72 | 26 | 2541 |
| 1161 | 1 | GA44500 | 19A | USA | 2005 | D | 8 | 13 | 14 | 4 | 4 | 4 | 14 | 2269 |
| 1162 | 1 | GA44511 | 19A | USA | 2005 | D | 7 | 11 | 10 | 1 | 6 | 198 | 1 | 4464 |
| 1163 | 1 | GA47033 | 6C | USA | 2005 | D | 1 | 13 | 9 | 12 | 94 | 4 | 20 | 4150 |
| 1164 | 1 | GA47281 | 19F | USA | 2006 | D | 4 | 16 | 19 | 15 | 6 | 20 | 189 | 3039 |
| 1165 | 1 | GA47283 | 7F | USA | 2006 | D | 8 | 9 | 2 | 1 | 6 | 1 | 17 | 191 |
| 1166 | 1 | GA47360 | 19A | USA | 2006 | D | 16 | 13 | 4 | 4 | 6 | 113 | 18 | 695 |
| 1167 | 1 | GA47368 | 19A | USA | 2006 | D | 6 | 11 | 1 | 67 | 15 | 72 | 14 | 1339 |
| 1168 | 1 | GA47373 | 19F | USA | 2006 | D | 18 | 2 | 22 | 16 | 9 | 23 | 14 | 654 |
| 1169 | 1 | GA47388 | 19A | USA | 2006 | D | 7 | 11 | 10 | 1 | 6 | 8 | 316 | 4026 |
| 1170 | 1 | GA47502 | 19A | USA | 2006 | D | 61 | 60 | 67 | 5 | 6 | 12 | 14 | 1374 |
| 1171 | 1 | GA47688 | 19A | USA | 2006 | D | 4 | 16 | 19 | 15 | 6 | 20 | 1 | 320 |
| 1172 | 1 | GA47751 | 19A | USA | 2006 | D | 6 | 11 | 1 | 67 | 15 | 72 | 8 | 2268 |
| 1173 | 1 | GA47778 | 19A | USA | 2006 | D | 4 | 16 | 19 | 15 | 6 | 20 | 1 | 320 |
| 1174 | 1 | GA47976 | 19F | USA | 2006 | D | 1 | 10 | 4 | 1 | 9 | 3 | 8 | 43 |
| 1175 | 1 | GA49138 | 19F | USA | 2006 | D | 15 | 5 | 19 | 15 | 6 | 20 | 26 | 651 |
| 1176 | 1 | GA49447 | 19A | USA | 2006 | D | 6 | 11 | 1 | 67 | 5 | 293 | 260 | 4176 |
| 1177 | 1 | GA52306 | 6C | USA | 2007 | D | 5 | 13 | 9 | 12 | 94 | 28 | 20 | 3676 |
| 1178 | 1 | GA54644 | 19A | USA | 2008 | D | 7 | 11 | 10 | 1 | 6 | 8 | 1 | 156 |
| 1179 | 1 | GA47439 | 7F | USA | 2006 | D | 10 | 20 | 8 | 10 | 6 | 1 | 29 | 1176 |
| 1180 | 1 | GA47901 | 1 | USA | 2006 | D | 13 | 8 | 13 | 5 | 17 | 4 | 8 | 304 |
| 1181 | 1 | 6735-05 | 19A | USA | 2005 | D | 8 | 13 | 14 | 4 | 17 | 4 | 1 | 2344 |
| 1182 | 1 | 6901-05 | 19A | USA | 2005 | D | 8 | 13 | 152 | 4 | 17 | 4 | 14 | 2381 |
| 1183 | 1 | 4027-06 | 19A | USA | 2005 | D | 8 | 13 | 14 | 4 | 17 | 4 | 1 | 2344 |
| 1184 | 1 | 6963-05 | 19A | USA | 2005 | D | 8 | 13 | 14 | 4 | 17 | 4 | 232 | 2392 |
| 1185 | 1 | 5787-06 | 19A | USA | 2005 | D | 8 | 13 | 14 | 4 | 17 | 77 | 97 | 2542 |
| 1186 | 1 | 5185-06 | 19A | USA | 2005 | D | 4 | 144 | 19 | 15 | 6 | 20 | 1 | 2393 |
| 1187 | 1 | 7286-06 | 19A | USA | 2005 | D | 4 | 16 | 19 | 67 | 6 | 202 | 1 | 2514 |
| 1188 | 1 | 3063-00 | 19F | USA | 1999 | D | 15 | 16 | 19 | 15 | 6 | 86 | 26 | 652 |
| 1189 | 1 | EU-NP01 | 19A | USA | ? | C | 16 | 13 | 4 | 4 | 6 | 113 | 18 | 695 |
| 1190 | 1 | NP112 | 6Bii | USA | ? | C | 7 | 6 | 1 | 17 | 6 | 1 | 104 | 1536 |
| 1191 | 1 | NP070 | 23F | USA | ? | C | 1 | 8 | 6 | 2 | 6 | 4 | 6 | 37 |
| 1192 | 1 | NP170 | 23F | USA | ? | C | 4 | 4 | 2 | 4 | 4 | 1 | 1 | 81 |
| 1193 | 1 | NP127 | 6A | USA | ? | C | 6 | 11 | 1 | 1 | 15 | 72 | 77 | 376 |
| 1194 | 1 | Sp07-2838 | 3 | Bolivia | 2007 | C | ? | 15 | 2 | 10 | 6 | 1 | 22 | ? |
| 1195 | 1 | Sp02-1198 | 3 | United Kingdom | 2002 | D | 7 | 15 | 2 | 10 | 6 | 1 | 22 | 180 |
| 1196 | AG | A45 | 3 | United Kingdom | ? | D | 10 | 9 | 4 | 12 | 287 | 426 | 470 | 6934 |
| 1197 | 1 | P1041 | 1 | Ghana | 2002 | D | 10 | 5 | 4 | 1 | 7 | 19 | 9 | 303 |
| 1198 | 1 | NCTC7465 | 1 | USA | 1948 | D | 10 | 31 | 4 | 1 | 6 | 4 | 94 | 615 |
| 1199 | 1 | Sp06-1370 | 1 | United Kingdom | ? | D | 12 | 8 | 13 | 5 | 16 | 4 | 20 | 306 |
| 1201 | 1 | GA17545 | 6Bii | USA | 2000 | D | 7 | 388 | 1 | 17 | 6 | 1 | 104 | 9057 |
| 1203 | 1 | PT8114 | 6C | USA | 2006 | ? | 10 | 13 | 1 | 43 | 98 | 1 | 20 | 1390 |
| 1204 | 1 | 07AR0125 | 6C | USA | 2006 | D | 1 | 13 | 9 | 12 | 94 | 28 | 20 | 1292 |
| 1205 | 1 | BR1064 | 6C | USA | 2006 | ? | 1 | 5 | 7 | 12 | 17 | 158 | 14 | 1692 |
| 1206 | 1 | ND6012 | 6C | USA | 2006 | ? | 1 | 5 | 7 | 12 | 17 | 158 | 14 | 1692 |
| 1210 | 1 | SB33 | 23F | Vietnam | 1997 | D | 4 | 4 | 2 | 4 | 4 | 1 | 1 | 81 |
| 1211 | 1 | HK P1 | 23F | China | 2000 | C | 4 | 4 | 2 | 4 | 4 | 1 | 1 | 81 |
| 1212 | 1 | SI M1 | 23F | Singapore | 2000 | C | 4 | 4 | 2 | 4 | 4 | 1 | 1 | 81 |
| 1213 | 1 | Kor 136 | 23F | Korea | 1998 | C | 4 | 4 | 2 | 4 | 4 | 1 | 1 | 81 |
| 1214 | AG | 412 | 23F | Croatia | 2001 | C | 4 | 4 | 2 | 4 | 4 | 1 | 1 | 81 |
| 1215 | 1 | 1788 | 19A | USA | 2005 | D | 4 | 4 | 2 | 5 | 4 | 1 | 1 | 2346 |
| 1216 | 1 | 2231 | 23F | USA | 1998 | D | 4 | 4 | 2 | 4 | 4 | 1 | 1 | 81 |
| 1217 | 1 | 2682 | 19A | USA | 2006 | D | 4 | 4 | 293 | 5 | 4 | 1 | 1 | 6220 |
| 1218 | 1 | BS1 | 23F | Turkey | 2005 | ? | 4 | 4 | 2 | 4 | 4 | 1 | 1 | 81 |
| 1219 | 1 | G104 | 23F | Turkey | 2005 | ? | 4 | 4 | 2 | 175 | 4 | 1 | 1 | 6219 |
| 1220 | 1 | S-030 | 6Bii | China | 2006 | ? | 4 | 4 | 2 | 4 | 6 | 1 | 1 | 83 |
| 1221 | 1 | SA4(Croucher) | 23F | South Africa | 2001 | C | 4 | 4 | 2 | 4 | 4 | 1 | 437 | 6216 |
| 1222 | 1 | SA7(Croucher) | 23F | South Africa | 2002 | C | 4 | 4 | 2 | 4 | 4 | 1 | 437 | 6216 |
| 1223 | 1 | SA17(Croucher) | 23F | South Africa | 2003 | D | 4 | 4 | 2 | 4 | 4 | 1 | 437 | 6216 |
| 1224 | 1 | SA27(Croucher) | 23F | South Africa | 2003 | D | 4 | 4 | 2 | 4 | 4 | 1 | 437 | 6216 |
| 1225 | 1 | SA61(Croucher) | 23F | South Africa | 2005 | D | 4 | 4 | 2 | 15 | 4 | 1 | 1 | 2395 |
| 1226 | 1 | SA78(Croucher) | 23F | South Africa | 2006 | D | 4 | 4 | 2 | 174 | 4 | 1 | 1 | 6218 |
| 1227 | 1 | SA142(Croucher) | 23F | South Africa | 1990 | C | 4 | 4 | 2 | 4 | 4 | 1 | 1 | 81 |
| 1228 | 1 | SA151(Croucher) | 23F | South Africa | 1989 | C | 4 | 4 | 2 | 4 | 4 | 8 | 1 | 6215 |
| 1229 | AG | 2859 | 23F | Germany | 1992 | D | 4 | 4 | 2 | 4 | 4 | 1 | 1 | 81 |
| 1230 | 1 | 8454 | 23F | Portugal | 2002 | D | 4 | 4 | 2 | 4 | 4 | 1 | 1 | 81 |
| 1231 | 1 | 23782 | 23F | Russia | 2003 | ? | 4 | 4 | 2 | 4 | 4 | 1 | 1 | 81 |
| 1232 | 1 | 23809 | 23F | Russia | 2005 | D | 4 | 4 | 2 | 4 | 4 | 1 | 1 | 81 |
| 1233 | 1 | BM4200 | 23F | France | 1978 | ? | 1 | 5 | 29 | 10 | 6 | 79 | 18 | 1010 |
| 1234 | 1 | cdc01 | 4 | USA | 1999 | D | 16 | 13 | 4 | 4 | 6 | 113 | 18 | 695 |
| 1235 | 1 | SA5(Croucher) | 23F | South Africa | 2001 | ? | 4 | 4 | 2 | 4 | 4 | 398 | 437 | 6217 |
| 1236 | 1 | cdc02 | 19A | USA | 1999 | D | 8 | 13 | 14 | 4 | 17 | 4 | 14 | 199 |
| 1237 | 1 | cdc13 | 19A | USA | 2003 | D | 16 | 13 | 4 | 4 | 6 | 113 | 18 | 695 |
| 1464 | 1 | VICE0913 | 15B/C | Iceland | 2009 | C | 8 | 13 | 14 | 4 | 17 | 4 | 14 | 199 |
| 2885 | 1 | 14/5 | 14 | Denmark | 1967 | ? | 1 | 5 | 4 | 5 | 5 | 3 | 8 | 15 |
| 2886 | 1 | 1/4 | 1 | Denmark | 1943 | ? | 12 | 8 | 13 | 5 | 17 | 4 | 8 | 5316 |
| 2887 | 1 | 2/2 | 2 | USA | 1956 | ? | 2 | 1 | 1 | 1 | 6 | 31 | 14 | 574 |
| 2888 | 1 | 2/3 | 2 | Denmark | 1943 | ? | 2 | 13 | 4 | 1 | 6 | 6 | 145 | 3744 |
| 2889 | 1 | 3/6 | 3 | Denmark | 1962 | ? | 10 | 336 | 28 | 11 | 17 | 111 | 17 | 7211 |
| 2890 | 1 | 4/4 | 4 | Denmark | 1962 | ? | 16 | 13 | 4 | 5 | 309 | 10 | 14 | 7223 |
| 2891 | 1 | 5/1 | 5 | USA | 1976 | ? | 1 | 103 | 14 | 8 | 6 | 105 | 29 | 4840 |
| 2892 | 1 | 6A/2 | 6A | USA | 1952 | ? | 5 | 7 | 4 | 5 | 10 | 1 | 27 | 7188 |
| 2893 | 1 | 6B/3 | 6B | Denmark | 1939 | ? | 7 | 8 | 9 | 1 | 17 | 3 | 20 | 7199 |
| 2894 | 1 | 7A/2 | 7A | Denmark | 1937 | ? | 8 | 9 | 2 | 1 | 6 | 1 | 17 | 191 |
| 2895 | 1 | 7B/2 | 7B | USA | 1952 | ? | 2 | 13 | 2 | 4 | 17 | 1 | 1 | 7180 |
| 2896 | 1 | 7C/3 | 7C | USA | 1971 | ? | 1 | 8 | 4 | 1 | 9 | 3 | 8 | 7171 |
| 2897 | 1 | 7F/2 | 7F | USA | 1952 | ? | 10 | 20 | 14 | 1 | 6 | 274 | 29 | 7210 |
| 2898 | 1 | 7F/4 | 7F | United Kingdom | 1986 | ? | 8 | 335 | 2 | 1 | 6 | 1 | 17 | 7208 |
| 2899 | 1 | 7F/5 | 7F | Denmark | 1962 | ? | 8 | 9 | 2 | 1 | 6 | 1 | 17 | 191 |
| 2900 | 1 | 8/4 | 8 | Denmark | 1962 | ? | 7 | 157 | 15 | 11 | 93 | 1 | 70 | 7203 |
| 2901 | 1 | 9A/1 | 9A | USA | 1962 | ? | 7 | 11 | 10 | 1 | 6 | 1 | 14 | 312 |
| 2902 | 1 | 9L/2 | 9L | USA | 1952 | ? | 7 | 16 | 1 | 8 | 14 | 11 | 14 | 5979 |
| 2903 | 1 | 9L/4 | 9L | Denmark | 1968 | ? | 7 | 16 | 8 | 8 | 6 | 49 | 14 | 7240 |
| 2904 | 1 | 9N/2 | 9N | USA | 1952 | ? | 8 | 5 | 7 | 12 | 6 | 16 | 6 | 7205 |
| 2905 | 1 | 9N/4 | 9N | Denmark | 1962 | ? | 2 | 13 | 2 | 4 | 6 | 1 | 1 | 71 |
| 2906 | 1 | 9N/6 | 9N | Denmark | 1960 | ? | 2 | 13 | 2 | 4 | 6 | 1 | 1 | 71 |
| 2907 | 1 | 9V/4 | 9V | Denmark | 1968 | ? | 7 | 2 | 40 | 1 | 10 | 1 | 45 | 123 |
| 2908 | 1 | 9V/5 | 9V | Denmark | 1991 | ? | 7 | 11 | 10 | 1 | 6 | 8 | 14 | 162 |
| 2909 | 1 | 9V/6 | 9V | Denmark | 1994 | ? | 7 | 11 | 10 | 1 | 6 | 8 | 14 | 162 |
| 2910 | 1 | 10A/1 | 10A | Denmark | 1938 | ? | 5 | 7 | 4 | 2 | 10 | 1 | 27 | 97 |
| 2911 | 1 | 10B/2 | 10B | Denmark | 1982 | ? | 1 | 5 | 9 | 16 | 6 | 4 | 197 | 5505 |
| 2912 | 1 | 10C/2 | 10C | Norway | 1983 | ? | 7 | 5 | 4 | 1 | 6 | 28 | 18 | 2862 |
| 2913 | 1 | 10F/2 | 10F | USA | 1956 | ? | 2 | 53 | 2 | 1 | 6 | 19 | 14 | 7186 |
| 2914 | 1 | 11A/2 | 11A | Denmark | 1939 | ? | 13 | 5 | 53 | 1 | 15 | 445 | 14 | 7214 |
| 2915 | 1 | 11B/2 | 11B | Denmark | 1940 | ? | 1 | 5 | 53 | 1 | 17 | 17 | 45 | 7165 |
| 2916 | 1 | 11C/1 | 11C | USA | 1957 | ? | 7 | 16 | 1 | 16 | 14 | 11 | 14 | 7201 |
| 2917 | 1 | 11D/1 | 11D | Denmark | 1986 | ? | 2 | 5 | 29 | 12 | 16 | 3 | 14 | 62 |
| 2918 | 1 | 11F/2 | 11F | Denmark | 1952 | ? | 12 | 281 | 4 | 1 | 15 | 1 | 1 | 7212 |
| 2919 | 1 | 12A/5 | 12A | Denmark | 1966 | ? | 1 | 2 | 7 | 18 | 17 | 14 | 17 | 5977 |
| 2920 | 1 | 12B/1 | 12B | Gambia | 1981 | ? | 2 | 153 | 111 | 1 | 15 | 20 | 15 | 7187 |
| 2921 | 1 | 12F/2 | 12F | USA | 1962 | ? | 7 | 2 | 4 | 10 | 197 | 444 | 15 | 7194 |
| 2922 | 1 | 12F/3 | 12F | Denmark | 1961 | ? | 18 | 5 | 4 | 1 | 310 | 1 | 6 | 7228 |
| 2923 | 1 | 12F/4 | 12F | USA | 1968 | ? | 7 | 2 | 4 | 10 | 197 | 444 | 15 | 7194 |
| 2924 | 1 | 12F/5 | 12F | Denmark | 1988 | ? | 10 | 20 | 14 | 1 | 6 | 1 | 29 | 218 |
| 2925 | 1 | 12F/6 | 12F | Denmark | 1996 | ? | 10 | 20 | 14 | 1 | 6 | 1 | 29 | 218 |
| 2926 | 1 | 13/2 | 13 | USA | 1952 | ? | 1 | 8 | 1 | 15 | 31 | 441 | 8 | 7173 |
| 2927 | 1 | 14/2 | 14 | USA | 1952 | ? | 7 | 5 | 1 | 8 | 14 | 11 | 14 | 124 |
| 2928 | 1 | 14/3 | 14 | Denmark | 1939 | ? | 8 | 8 | 4 | 15 | 17 | 12 | 31 | 875 |
| 2929 | 1 | 14/4 | 14 | Denmark | 1961 | ? | 7 | 5 | 1 | 8 | 14 | 1 | 14 | 134 |
| 2930 | 1 | 14/7 | 14 | Denmark | 1992 | ? | 7 | 5 | 1 | 8 | 14 | 11 | 14 | 124 |
| 2931 | 1 | 14/8 | 14 | Denmark | 1976 | ? | 8 | 8 | 4 | 15 | 39 | 12 | 498 | 7206 |
| 2932 | 1 | 14/9 | 14 | Denmark | 1982 | ? | 7 | 5 | 1 | 8 | 14 | 11 | 14 | 124 |
| 2933 | 1 | 15A/2 | 15A | Denmark | 1939 | ? | 1 | 5 | 12 | 5 | 9 | 15 | 5 | 7166 |
| 2934 | 1 | 15B/2 | 15B/C | Denmark | 1939 | ? | 1 | 1 | 1 | 1 | 1 | 1 | 1 | 1 |
| 2935 | 1 | 15C/2 | 15B/C | Denmark | 1962 | ? | 1 | 8 | 1 | 1 | 1 | 1 | 1 | 7172 |
| 2936 | 1 | 15F/3 | 15F | Denmark | 1963 | ? | 2 | 5 | 4 | 66 | 10 | 1 | 11 | 7177 |
| 2937 | 1 | 16A/1 | 16A | USA | 1985 | ? | 5 | 300 | 1 | 16 | 17 | 79 | 14 | 6543 |
| 2938 | 1 | 16F/2 | 16F | USA | 1952 | ? | 1 | 5 | 4 | 4 | 6 | 58 | 14 | 7167 |
| 2939 | 1 | 17A/2 | 17A | Germany | 1971 | ? | 18 | 12 | 2 | 16 | 6 | 19 | 245 | 2599 |
| 2940 | 1 | 17F/2 | 17F | Denmark | 1939 | ? | 7 | 2 | 40 | 1 | 10 | 1 | 45 | 123 |
| 2941 | 1 | 17F/3 | 17F | Denmark | 1962 | ? | 7 | 5 | 1 | 1 | 6 | 31 | 14 | 392 |
| 2942 | 1 | 17F/4 | 17F | Denmark | 1962 | ? | 7 | 5 | 1 | 1 | 6 | 31 | 14 | 392 |
| 2943 | 1 | 18A/2 | 18A | Denmark | 1952 | ? | 25 | 31 | 4 | 16 | 32 | 28 | 44 | 241 |
| 2944 | 1 | 18C/1 | 18C | Denmark | 1940 | ? | 7 | 2 | 1 | 1 | 10 | 1 | 6 | 4706 |
| 2945 | 1 | 18C/2 | 18C | Denmark | 1939 | ? | 7 | 2 | 1 | 1 | 10 | 1 | 6 | 4706 |
| 2946 | 1 | 18C/3 | 18C | Denmark | 1968 | ? | 7 | 2 | 1 | 2 | 10 | 1 | 6 | 7195 |
| 2947 | 1 | 18F/1 | 18F | USA | 1961 | ? | 2 | 13 | 1 | 1 | 6 | 31 | 14 | 7182 |
| 2948 | 1 | 19A/5 | 19A | Denmark | 1968 | ? | 18 | 5 | 9 | 1 | 9 | 1 | 14 | 7226 |
| 2949 | 1 | 19B/2 | 19B | Germany | 1971 | ? | 8 | 13 | 14 | 4 | 17 | 4 | 14 | 199 |
| 2950 | 1 | 19C/2 | 19C | Denmark | 1939 | ? | 2 | 13 | 2 | 12 | 17 | 1 | 14 | 3785 |
| 2951 | 1 | 19F/3 | 19F | Denmark | 1961 | ? | 1 | 5 | 1 | 5 | 1 | 1 | 8 | 425 |
| 2952 | 1 | 19F/5 | 19F | Denmark | 1962 | ? | 18 | 5 | 4 | 1 | 15 | 1 | 6 | 7229 |
| 2953 | 1 | 19F/8 | 19F | Denmark | 1952 | ? | 18 | 5 | 4 | 1 | 15 | 1 | 6 | 7229 |
| 2954 | 1 | 19F/10 | 19F | Denmark | 1963 | ? | 18 | 5 | 13 | 1 | 27 | 1 | 6 | 7230 |
| 2955 | 1 | 20/3 | 20B | Denmark | 1937 | ? | 15 | 8 | 8 | 18 | 15 | 1 | 31 | 235 |
| 2956 | 1 | 21/3 | 21 | Denmark | 1962 | ? | 8 | 10 | 2 | 16 | 1 | 26 | 1 | 193 |
| 2957 | 1 | 22A/2 | 22A | Denmark | 1939 | ? | 2 | 13 | 4 | 1 | 6 | 31 | 14 | 7181 |
| 2958 | 1 | 22F/2 | 22F | Denmark | 1940 | ? | 13 | 161 | 34 | 1 | 6 | 1 | 6 | 7219 |
| 2959 | 1 | 23A/2 | 23A | Denmark | 1945 | ? | 1 | 8 | 9 | 2 | 6 | 4 | 6 | 439 |
| 2960 | 1 | 23B/1 | 23B | Denmark | 1941 | ? | 1 | 5 | 4 | 4 | 15 | 1 | 8 | 7168 |
| 2961 | 1 | 23F/4 | 23F | Australia | 1967 | ? | 2 | 22 | 2 | 4 | 308 | 1 | 1 | 7184 |
| 2962 | 1 | 23F/5 | 23F | Germany | 1979 | ? | 1 | 8 | 9 | 2 | 6 | 4 | 6 | 439 |
| 2963 | 1 | 23F/10 | 23F | Denmark | 1996 | ? | 16 | 8 | 9 | 1 | 6 | 4 | 72 | 515 |
| 2964 | 1 | 24A/2 | 24A | Denmark | 1940 | ? | 2 | 9 | 9 | 1 | 6 | 17 | 9 | 5980 |
| 2965 | 1 | 24B/2 | 24B | Denmark | 1942 | ? | 13 | 5 | 40 | 134 | 1 | 1 | 18 | 7215 |
| 2966 | 1 | 24F/2 | 24F | USA | 1952 | ? | 1 | 8 | 4 | 16 | 307 | 20 | 9 | 7174 |
| 2967 | 1 | 25A/1 | 25A | Singapore | 1981 | ? | 10 | 5 | 87 | 18 | 10 | 49 | 6 | 7209 |
| 2968 | 1 | 25F/4 | 25F | Denmark | 1962 | ? | 5 | 15 | 4 | 1 | 6 | 1 | 6 | 105 |
| 2969 | 1 | 27/2 | 27 | USA | 1956 | ? | 2 | 17 | 107 | 24 | 9 | 105 | 14 | 1475 |
| 2970 | 1 | 28F/2 | 28A | USA | 1957 | ? | 18 | 5 | 4 | 1 | 6 | 77 | 14 | 7227 |
| 2971 | 1 | 29/2 | 29 | USA | 1952 | ? | 2 | 12 | 2 | 8 | 6 | 1 | 14 | 5982 |
| 2972 | 1 | 32F/2 | 32F | USA | 1952 | ? | 1 | 2 | 4 | 62 | 6 | 1 | 18 | 7164 |
| 2973 | 1 | 33A/2 | 33A | USA | 1946 | ? | 2 | 5 | 29 | 18 | 42 | 3 | 18 | 1012 |
| 2974 | 1 | 33B/2 | 33B | Denmark | 1962 | ? | 16 | 5 | 166 | 1 | 13 | 14 | 18 | 2864 |
| 2975 | 1 | 33D/2 | 33D | India | 1979 | ? | 2 | 8 | 4 | 10 | 17 | 1 | 9 | 2863 |
| 2976 | 1 | 33F/2 | 33F | Denmark | 1937 | ? | 5 | 35 | 29 | 12 | 9 | 45 | 18 | 7192 |
| 2977 | 1 | 34/5 | 34 | Denmark | 1974 | ? | 13 | 5 | 1 | 12 | 9 | 1 | 18 | 7216 |
| 2978 | 1 | 35A/3 | 35A | Denmark | 1939 | ? | 326 | 13 | 2 | 16 | 43 | 1 | 79 | 7242 |
| 2979 | 1 | 35B/2 | 35B | Denmark | 1939 | ? | 18 | 12 | 1 | 1 | 14 | 77 | 14 | 7234 |
| 2980 | 1 | 35C/2 | 35C | Denmark | 1941 | ? | 7 | 2 | 9 | 1 | 10 | 1 | 45 | 7196 |
| 2981 | 1 | 35C/3 | 35C | Denmark | 1943 | ? | 7 | 2 | 1 | 1 | 10 | 1 | 45 | 5989 |
| 2982 | 1 | 35F/2 | 35F | Denmark | 1939 | ? | 1 | 2 | 4 | 62 | 6 | 1 | 18 | 7164 |
| 2983 | 1 | 36/2 | 36 | Denmark | 1939 | ? | 109 | 12 | 1 | 1 | 6 | 1 | 18 | 7237 |
| 2984 | 1 | 37/6 | 37 | Denmark | 1973 | ? | 192 | 34 | 19 | 1 | 36 | 22 | 445 | 7243 |
| 2985 | 1 | 38/2 | 38 | Denmark | 1939 | ? | 10 | 43 | 41 | 18 | 13 | 49 | 6 | 393 |
| 2986 | 1 | 39/2 | 39 | Denmark | 1940 | ? | 5 | 7 | 329 | 1 | 13 | 1 | 27 | 7189 |
| 2987 | 1 | 40/2 | 40 | USA | 1950 | ? | 2 | 31 | 1 | 1 | 236 | 335 | 6 | 7185 |
| 2988 | 1 | 41A/2 | 41A | Germany | 1972 | ? | 226 | 5 | 1 | 1 | 10 | 28 | 120 | 7238 |
| 2989 | 1 | 41F/2 | 41F | Denmark | 1940 | ? | 13 | 2 | 69 | 1 | 9 | 1 | 14 | 7213 |
| 2990 | 1 | 42/2 | 42 | Denmark | 1971 | ? | 1 | 1 | 4 | 1 | 18 | 58 | 17 | 433 |
| 2991 | 1 | 43/3 | 43 | Denmark | 1948 | ? | 7 | 5 | 1 | 10 | 10 | 16 | 6 | 7197 |
| 2992 | 1 | 44/3 | 44 | Germany | 1956 | ? | 1 | 194 | 274 | 1 | 14 | 77 | 1 | 7176 |
| 2993 | 1 | 45/5 | 45 | USA | 1954 | ? | 5 | 8 | 8 | 1 | 6 | 19 | 497 | 7190 |
| 2994 | 1 | 46/2 | 46 | USA | 1954 | ? | 7 | 5 | 4 | 10 | 9 | 3 | 8 | 7162 |
| 2995 | 1 | 47A/1 | 47A | ? | 1977 | ? | 15 | 8 | 8 | 5 | 15 | 1 | 31 | 7220 |
| 2996 | 1 | 47F/1 | 47F | ? | 1977 | ? | 13 | 280 | 53 | 17 | 14 | 1 | 31 | 5991 |
| 2997 | 1 | 48/3 | 48 | Denmark | 1963 | ? | 18 | 9 | 4 | 32 | 6 | 1 | 413 | 7232 |
| 3080 | 1 | D107 | 23F | Germany | 1986 | ? | 7 | 13 | 8 | 6 | 6 | 12 | 8 | 277 |
| 3081 | 1 | D110 | NT | Germany | 1992 | ? | 8 | 29 | 4 | 15 | 17 | 12 | 31 | 1106 |
| 3082 | 1 | D111 | 6Bii | Germany | 1992 | D | 5 | 6 | 1 | 2 | 6 | 3 | 4 | 90 |
| 3083 | 1 | D206 | 9V | Germany | 1985 | ? | 1 | 17 | 4 | 16 | 10 | 1 | 17 | 13139 |
| 3084 | 1 | D211 | 19F | Germany | 1985 | D | 8 | 10 | 2 | 5 | 9 | 48 | 6 | 309 |
| 3085 | 1 | D214 | 19F | Germany | 1988 | D | 15 | 16 | 19 | 15 | 6 | 20 | 26 | 236 |
| 3086 | 1 | D219 | 23F | Germany | 1989 | D | 1 | 5 | 9 | 5 | 6 | 3 | 65 | 353 |
| 3087 | 1 | 2301 | 23A | Finland | 1985 | D | 1 | 8 | 9 | 2 | 6 | 35 | 6 | 524 |
| 3088 | 1 | 2302 | 23F | Finland | 1985 | D | 1 | 8 | 6 | 2 | 6 | 4 | 6 | 37 |
| 3089 | 1 | 2303 | 23F | Finland | 1985 | D | 7 | 8 | 6 | 2 | 6 | 4 | 6 | 13140 |
| 3090 | 1 | 2305 | 23F | Finland | 1986 | D | 1 | 8 | 6 | 2 | 6 | 4 | 6 | 37 |
| 3091 | 1 | 2306 | 23F | Finland | 1986 | D | 16 | 8 | 9 | 2 | 6 | 4 | 14 | 13141 |
| 3092 | 1 | 2307 | 23F | Finland | 1986 | D | 1 | 8 | 1 | 2 | 6 | 4 | 6 | 33 |
| 3093 | 1 | 2309 | 23F | Finland | 1987 | D | 7 | 5 | 1 | 1 | 13 | 31 | 14 | 440 |
| 3094 | 1 | 2310 | 23F | Finland | 1987 | D | 7 | 5 | 1 | 1 | 13 | 31 | 14 | 440 |
| 3095 | 1 | Fi11 | 6Bii | Finland | 1988 | D | 5 | 6 | 1 | 2 | 6 | 1 | 28 | 238 |
| 3096 | 1 | Fi12 | 6Bii | Finland | 1987 | D | 5 | 6 | 1 | 2 | 6 | 1 | 28 | 238 |
| 3097 | 1 | Fi14 | 23F | Finland | 1986 | C | 1 | 5 | 29 | 10 | 6 | 79 | 18 | 1010 |
| 3098 | 1 | Fi15 | 23F | Finland | 1986 | D | 1 | 5 | 29 | 10 | 6 | 79 | 18 | 1010 |
| 3099 | 1 | Fi16 | 23F | Finland | 1986 | C | 1 | 5 | 29 | 10 | 6 | 79 | 18 | 1010 |
| 3100 | 1 | Fi17 | 19A | Finland | 1987 | D | 1 | 13 | 2 | 5 | 19 | 26 | 8 | 13142 |
| 3102 | 1 | U1 | 6B | Hungary | 1990 | ? | 8 | 13 | 9 | 1 | 6 | 12 | 14 | 13143 |
| 3103 | 1 | U2 | 19A | Hungary | 1990 | ? | 7 | 13 | 42 | 6 | 10 | 6 | 114 | 13144 |
| 3104 | 1 | U3 | 23F | Hungary | 1990 | ? | 7 | 13 | 2 | 6 | 6 | 6 | 14 | 7799 |
| 3105 | 1 | U6 | 19A | Hungary | 1990 | ? | 7 | 13 | 42 | 6 | 10 | 6 | 14 | 226 |
| 3106 | 1 | U9 | 19A | Hungary | 1990 | ? | 7 | 13 | 42 | 6 | 10 | 6 | 14 | 226 |
| 3107 | 1 | U11 | 19A | Hungary | 1990 | ? | 7 | 13 | 42 | 6 | 10 | 6 | 14 | 226 |
| 3108 | 1 | U12 | 19A | Hungary | 1989 | ? | 7 | 13 | 42 | 6 | 10 | 6 | 14 | 226 |
| 3109 | 1 | U13 | 19A | Hungary | 1991 | ? | 7 | 13 | 42 | 6 | 10 | 6 | 56 | 268 |
| 3110 | 1 | U15 | 19A | Hungary | 1991 | ? | 7 | 13 | 42 | 6 | 10 | 6 | 14 | 226 |
| 3111 | 1 | U16 | 19A | Hungary | 1991 | ? | 7 | 13 | 42 | 6 | 10 | 6 | 14 | 226 |
| 3112 | 1 | U18 | 6B | Hungary | 1991 | ? | 2 | 13 | 9 | 1 | 6 | 19 | 848 | 13145 |
| 3113 | 1 | U20 | 19A | Hungary | 1990 | ? | 7 | 13 | 42 | 6 | 10 | 6 | 115 | 13146 |
| 3114 | 1 | U22 | 23F | Hungary | 1991 | ? | 4 | 4 | 2 | 4 | 4 | 1 | 1 | 81 |
| 3115 | 1 | U23 | 19A | Hungary | 1992 | ? | 7 | 13 | 42 | 6 | 10 | 6 | 14 | 226 |
| 3116 | 1 | U26 | 6B | Hungary | 1990 | ? | 7 | 25 | 4 | 4 | 15 | 20 | 28 | 473 |
| 3117 | 1 | 29044 | 14 | Czechoslovakia | 1987 | ? | 1 | 5 | 4 | 1 | 5 | 3 | 3 | 20 |
| 3118 | 1 | Pn16 | 42 | PNG | 1970 | ? | 4 | 4 | 2 | 4 | 4 | 1 | 1 | 81 |
| 3119 | 1 | IS1 | 6B | Israel | 1987 | D | 7 | 8 | 1 | 1 | 15 | 14 | 14 | 1093 |
| 3120 | 1 | IS5 | 14 | Israel | 1987 | D | 1 | 5 | 181 | 405 | 5 | 3 | 50 | 13136 |
| 3121 | 1 | IS3 | 19A | Israel | 1989 | D | 13 | 5 | 62 | 8 | 9 | 1 | 133 | 2929 |
| 3122 | 1 | IS6 | 14 | Israel | 1989 | D | 2 | 5 | 36 | 12 | 17 | 21 | 14 | 63 |
| 3123 | 1 | IS7 | 14 | Israel | 1990 | D | 12 | 19 | 2 | 17 | 6 | 22 | 14 | 230 |
| 3124 | 1 | SA6_RH | 14 | South Africa | 1978 | D | 1 | 5 | 5 | 30 | 88 | 88 | 6 | 13147 |
| 3125 | 1 | SA17_RH | 23F | South Africa | 1987 | D | 4 | 4 | 2 | 4 | 4 | 1 | 1 | 81 |
| 3126 | 1 | 8249 | 19A | South Africa | 1977 | ? | 2 | 8 | 9 | 5 | 11 | 1 | 13 | 1656 |
| 3127 | 1 | s 1° | 13 | South Africa | 1989 | ? | 15 | 5 | 54 | 1 | 197 | 1 | 168 | 5647 |
| 3128 | 1 | s 2° | 4 | South Africa | 1989 | ? | 16 | 2 | 40 | 5 | 241 | 20 | 1 | 10838 |
| 3129 | 1 | s 5° | 11B | South Africa | 1989 | ? | 18 | 9 | 2 | 16 | 6 | 20 | 245 | 5969 |
| 3130 | 1 | s 6° | 12B | South Africa | 1989 | ? | 8 | 4 | 53 | 10 | 59 | 1 | 14 | 13148 |
| 3131 | 1 | s 7° | 39 | South Africa | 1989 | ? | 2 | 13 | 54 | 16 | 6 | 1 | 5 | 13149 |
| 3132 | 1 | s 8° | 12F | South Africa | 1989 | ? | 8 | 4 | 53 | 10 | 59 | 1 | 14 | 13148 |
| 3133 | 1 | s 9° | 6A | South Africa | 1989 | ? | 8 | 9 | 4 | 13 | 15 | 20 | 28 | 8696 |
| 3134 | 1 | s 10° | 39 | South Africa | 1989 | ? | 2 | 13 | 54 | 16 | 6 | 1 | 5 | 13149 |
| 3135 | 1 | A | 22F | South Africa | 1978 | D | 1 | 5 | 4 | 20 | 15 | 16 | 20 | 13152 |
| 3136 | 1 | B | 6B | South Africa | 1984 | ? | 2 | 25 | 1 | 2 | 6 | 220 | 14 | 13153 |
| 3137 | 1 | E | 1 | South Africa | 1984 | ? | 10 | 9 | 4 | 1 | 6 | 4 | 94 | 611 |
| 3138 | 1 | F | 6B | South Africa | 1986 | ? | 7 | 62 | 1 | 2 | 6 | 20 | 423 | 7585 |
| 3139 | 1 | 456 | 23F | Spain | 1984 | D | 4 | 4 | 2 | 4 | 4 | 1 | 1 | 81 |
| 3140 | 1 | 496 | 19F | Spain | 1985 | D | 4 | 4 | 2 | 4 | 4 | 1 | 1 | 81 |
| 3141 | 1 | 628 | 9V | Spain | 1987 | D | 7 | 11 | 10 | 1 | 6 | 8 | 1 | 156 |
| 3142 | 1 | 632 | 23F | Spain | 1987 | D | 4 | 4 | 2 | 4 | 4 | 1 | 1 | 81 |
| 3143 | 1 | 637 | 23F | Spain | 1987 | D | 4 | 4 | 2 | 4 | 4 | 1 | 1 | 81 |
| 3144 | 1 | 638 | 23F | Spain | 1987 | D | 4 | 4 | 2 | 4 | 4 | 1 | 1 | 81 |
| 3145 | 1 | 639 | 23F | Spain | 1987 | D | 4 | 4 | 2 | 4 | 4 | 1 | 1 | 81 |
| 3146 | 1 | 640 | 19F | Spain | 1987 | D | 5 | 5 | 7 | 10 | 567 | 40 | 7 | 13137 |
| 3147 | 1 | 644 | 14 | Spain | 1988 | D | 1 | 5 | 4 | 11 | 9 | 3 | 47 | 17 |
| 3148 | 1 | 652 | 6B | Spain | 1987 | D | 5 | 6 | 1 | 2 | 6 | 3 | 4 | 90 |
| 3149 | 1 | 653 | 23F | Spain | 1987 | D | 4 | 4 | 2 | 4 | 4 | 1 | 1 | 81 |
| 3150 | 1 | 655 | 6Bii | Spain | 1986 | D | 5 | 6 | 1 | 2 | 6 | 3 | 4 | 90 |
| 3151 | 1 | 662 | 6Bii | Spain | 1986 | D | 5 | 6 | 1 | 2 | 6 | 3 | 4 | 90 |
| 3152 | 1 | 663 | 23F | Spain | 1988 | D | 4 | 4 | 2 | 4 | 4 | 1 | 1 | 81 |
| 3153 | 1 | 664 | 6Bii | Spain | 1986 | D | 5 | 6 | 1 | 2 | 6 | 3 | 4 | 90 |
| 3154 | 1 | 665 | 9V | Spain | 1988 | D | 7 | 11 | 10 | 1 | 6 | 8 | 1 | 156 |
| 3155 | 1 | 667 | 23F | Spain | 1988 | D | 4 | 4 | 2 | 4 | 4 | 1 | 1 | 81 |
| 3156 | 1 | 668 | 6Bii | Spain | 1986 | D | 5 | 6 | 1 | 2 | 6 | 3 | 847 | 13138 |
| 3157 | 1 | 670_RH | 6Bii | Spain | 1988 | D | 5 | 6 | 1 | 2 | 6 | 3 | 4 | 90 |
| 3158 | 1 | 672 | 6A | Spain | 1988 | D | 5 | 6 | 1 | 2 | 6 | 3 | 4 | 90 |
| 3159 | 1 | 673 | 23F | Spain | 1988 | D | 4 | 4 | 2 | 4 | 4 | 1 | 1 | 81 |
| 3160 | 1 | 674 | 23F | Spain | 1988 | D | 4 | 4 | 2 | 4 | 4 | 1 | 1 | 81 |
| 3161 | 1 | 676 | 9V | Spain | 1988 | D | 7 | 11 | 10 | 1 | 6 | 8 | 1 | 156 |
| 3162 | 1 | 677 | 23F | Spain | 1988 | D | 4 | 4 | 2 | 4 | 4 | 1 | 1 | 81 |
| 3163 | 1 | 679 | 9V | Spain | 1988 | C | 7 | 11 | 10 | 1 | 6 | 8 | 1 | 156 |
| 3164 | 1 | 680 | 9V | Spain | 1988 | D | 7 | 11 | 10 | 1 | 6 | 8 | 1 | 156 |
| 3165 | 1 | 681 | 6Bii | Spain | 1986 | D | 5 | 6 | 1 | 2 | 6 | 3 | 4 | 90 |
| 8291 | 1 | ST556 | 19F | USA | ? | ? | 15 | 16 | 19 | 15 | 6 | 20 | 156 | 1392 |
