# Supplementary material for: Diverse Streptococcus pneumoniae Strains Drive a Mucosal-Associated Invariant T-Cell Response Through Major Histocompatibility Complex class I–Related Molecule–Dependent and Cytokine-Driven Pathways
Source: J Infect Dis. 2017 Dec 15;217(6):988–99. doi: 10.1093/infdis/jix647 (PMC5854017; doi:10.1093/infdis/jix647)
Supplement: Supplementary Table 2 [file jix647_suppl_supplementary_table_2.docx]

Supplementary Table 2. Descriptive data for the 824 non-pneumococcal Streptococcus species genomes included in this study.

| **BIGSdb**  **id** | **rMLST**  **id** | **Riboflavin operon version** | **Specie** | **Strain** |
| --- | --- | --- | --- | --- |
| 438 | 167 | 2 | *Streptococcus agalactiae* | 2603V/R |
| 503 | 168 | 2 | *Streptococcus agalactiae* | NEM316 |
| 568 | 169 | 2 | *Streptococcus agalactiae* | A909 |
| 933 | 170 | Not found | *Streptococcus gordonii* | Challis |
| 1259 | 172 | Not found | *Streptococcus mutans* | NN2025 |
| 1271 | 173 | Not found | *Streptococcus mutans* | UA159 |
| 1289 | 174 | Not found | *Streptococcus pyogenes* | M1 GAS |
| 1294 | 175 | Not found | *Streptococcus pyogenes* | MGAS315 |
| 1337 | 176 | Not found | *Streptococcus pyogenes* | MGAS2096 |
| 1343 | 177 | Not found | *Streptococcus pyogenes* | MGAS5005 |
| 1345 | 178 | Not found | *Streptococcus pyogenes* | MGAS6180 |
| 1346 | 179 | Not found | *Streptococcus pyogenes* | MGAS8232 |
| 1347 | 180 | Not found | *Streptococcus pyogenes* | MGAS9429 |
| 1381 | 182 | Not found | *Streptococcus pyogenes* | MGAS10394 |
| 1395 | 183 | Not found | *Streptococcus pyogenes* | MGAS10750 |
| 1403 | 184 | Not found | *Streptococcus pyogenes* | NZ131 |
| 1413 | 185 | Not found | *Streptococcus pyogenes* | SSI-1 |
| 1418 | 186 | Not found | *Streptococcus pyogenes* | Manfredo |
| 1436 | 187 | Not found | *Streptococcus sanguinis* | SK36 |
| 1437 | 285 | Not found | *Streptococcus suis* | 98HAH33 |
| 1652 | 286 | Not found | *Streptococcus suis* | BM407 |
| 2059 | 287 | Not found | *Streptococcus suis* | P1/7 |
| 2075 | 289 | Not found | *Streptococcus thermophilus* | CNRZ1066 |
| 2097 | 290 | Not found | *Streptococcus thermophilus* | LMD-9 |
| 2104 | 291 | Not found | *Streptococcus thermophilus* | LMG 18311 |
| 2106 | 292 | Not found | *Streptococcus uberis* | 0140J |
| 2469 | 411 | Not found | *Streptococcus suis* | 05ZYH33 |
| 2487 | 872 | 7 | *Streptococcus gallolyticus* | UCN34 |
| 2493 | 1252 | 5 | *Streptococcus infantarius* | ATCC BAA-102 |
| 2494 | 1271 | Not found | *Streptococcus salivarius* | SK126 |
| 2495 | 1648 | Not found | *Streptococcus equi* | 4047 |
| 2496 | 1655 | Not found | *Streptococcus equi* | H70 |
| 2497 | 1656 | Not found | *Streptococcus equi* | MGCS10565 |
| 2499 | 9646 | Not found | *Streptococcus pyogenes* | ERR172100 |
| 2500 | 9680 | Not found | *Streptococcus pyogenes* | ERR111857 |
| 2504 | 9684 | Not found | *Streptococcus pyogenes* | ERR111974 |
| 2505 | 9725 | Not found | *Streptococcus pyogenes* | ERR111918 |
| 2509 | 9728 | Not found | *Streptococcus pyogenes* | ERR111888 |
| 2514 | 9729 | Not found | *Streptococcus pyogenes* | ERR111948 |
| 2521 | 9801 | Not found | *Streptococcus pyogenes* | ERR111845 |
| 2528 | 9806 | Not found | *Streptococcus pyogenes* | ERR111864 |
| 2536 | 9807 | Not found | *Streptococcus pyogenes* | SRR125399 |
| 2537 | 9813 | Not found | *Streptococcus pyogenes* | ERR111909 |
| 2538 | 9827 | Not found | *Streptococcus pyogenes* | ERR111856 |
| 2548 | 9829 | Not found | *Streptococcus pyogenes* | ERR040144 |
| 2550 | 9858 | Not found | *Streptococcus pyogenes* | ERR111889 |
| 2553 | 9933 | Not found | *Streptococcus pyogenes* | SRR016703 |
| 2662 | 10055 | Not found | *Streptococcus pyogenes* | ERR172141 |
| 2663 | 54967 | Not found | *Streptococcus pyogenes* | ERR369064 |
| 2680 | 55104 | Not found | *Streptococcus pyogenes* | ERR369104 |
| 2783 | 55717 | Not found | *Streptococcus anginosus* | C1051 |
| 2784 | 55718 | Not found | *Streptococcus anginosus* | C238 |
| 2785 | 55719 | Not found | *Streptococcus constellatus* | C1050 |
| 2847 | 55720 | Not found | *Streptococcus constellatus* | C232 |
| 2880 | 55721 | Not found | *Streptococcus constellatus* | C818 |
| 2882 | 55722 | Not found | *Streptococcus iniae* | SF1 |
| 2883 | 55723 | Not found | *Streptococcus intermedius* | B196 |
| 2884 | 55724 | Not found | *Streptococcus intermedius* | C270 |
| 2999 | 55725 | Not found | *Streptococcus intermedius* | JTH08 |
| 3003 | 55726 | 4 | *Streptococcus lutetiensis* | 33 |
| 3006 | 55727 | Not found | *Streptococcus oligofermentans* | AS 1.3089 |
| 3007 | 55729 | Not found | *Streptococcus parasanguinis* | ATCC 15912 |
| 3033 | 55730 | Not found | *Streptococcus parasanguinis* | FW213 |
| 3053 | 55731 | Not found | *Streptococcus parauberis* | KCTC 11537 |
| 3054 | 55732 | 7 | *Streptococcus pasteurianus* | ATCC 43144 |
| 3060 | 57285 | Not found | *Streptococcus pyogenes* | ERR374658 |
| 3077 | 58717 | Not found | *Streptococcus macedonicus* | ACA-DC 198 |
| 3171 | 58901 | Not found | *Streptococcus australis* | ATCC 700641 |
| 3180 | 58915 | Not found | *Streptococcus infantis* | ATCC 700779 |
| 3224 | 60014 | 6 | *Streptococcus caballi* | DSM 19004 |
| 3237 | 60015 | Not found | *Streptococcus canis* | FSL Z3-227 |
| 3248 | 60016 | Not found | *Streptococcus criceti* | HS-6 |
| 3329 | 60017 | Not found | *Streptococcus didelphis* | DSM 15616 |
| 3400 | 60018 | Not found | *Streptococcus downei* | F0415 |
| 3402 | 60019 | Not found | *Streptococcus entericus* | DSM 14446 |
| 3415 | 60021 | 3 | *Streptococcus equinus* | ATCC 9812 |
| 3424 | 60022 | Not found | *Streptococcus ferus* | DSM 20646 |
| 3425 | 60023 | 11 | *Streptococcus henryi* | DSM 19005 |
| 3429 | 60024 | Not found | *Streptococcus ictaluri* | 707-05 |
| 3462 | 60025 | Not found | *Streptococcus infantis* | SK1076 |
| 3509 | 60027 | Not found | *Streptococcus infantis* | SK970 |
| 3510 | 60028 | Not found | *Streptococcus infantis* | PRJNA179863 |
| 3566 | 60029 | Not found | *Streptococcus macacae* | NCTC 11558 |
| 3590 | 60030 | Not found | *Streptococcus marimammalium* | DSM 18627 |
| 3596 | 60031 | Not found | *Streptococcus massiliensis* | DSM 18628 |
| 3637 | 60032 | Not found | *Streptococcus merionis* | DSM 19192 |
| 3646 | 60033 | Not found | *Streptococcus minor* | DSM 17118 |
| 3709 | 60034 | Not found | *Streptococcus orisratti* | DSM 15617 |
| 3733 | 60035 | Not found | *Streptococcus ovis* | DSM 16829 |
| 3738 | 60036 | Not found | *Streptococcus peroris* | ATCC 700780 |
| 3741 | 60038 | Not found | *Streptococcus porcinus* | Jelinkova 176 |
| 3745 | 60039 | Not found | *Streptococcus pseudoporcinus* | LQ 940-04 |
| 3751 | 60040 | Not found | *Streptococcus pseudoporcinus* | SPIN 20026 |
| 3752 | 60041 | Not found | *Streptococcus sobrinus* | TCI-107 |
| 3756 | 60042 | Not found | *Streptococcus sobrinus* | TCI-118 |
| 3762 | 60043 | Not found | *Streptococcus sobrinus* | TCI-119 |
| 3765 | 60044 | Not found | *Streptococcus sobrinus* | TCI-124 |
| 3766 | 60045 | Not found | *Streptococcus sobrinus* | TCI-13 |
| 3767 | 60046 | Not found | *Streptococcus sobrinus* | TCI-16 |
| 3768 | 60047 | Not found | *Streptococcus sobrinus* | TCI-160 |
| 3769 | 60048 | Not found | *Streptococcus sobrinus* | TCI-172 |
| 3770 | 60049 | Not found | *Streptococcus sobrinus* | TCI-194 |
| 3771 | 60050 | Not found | *Streptococcus sobrinus* | TCI-2 |
| 3772 | 60051 | Not found | *Streptococcus sobrinus* | TCI-200 |
| 3773 | 60052 | Not found | *Streptococcus sobrinus* | TCI-215 |
| 3774 | 60053 | Not found | *Streptococcus sobrinus* | TCI-28 |
| 3775 | 60054 | Not found | *Streptococcus sobrinus* | TCI-342 |
| 3776 | 60055 | Not found | *Streptococcus sobrinus* | TCI-345 |
| 3777 | 60058 | Not found | *Streptococcus sobrinus* | TCI-352 |
| 3778 | 60059 | Not found | *Streptococcus sobrinus* | TCI-355 |
| 3779 | 60060 | Not found | *Streptococcus sobrinus* | TCI-357 |
| 3780 | 60061 | Not found | *Streptococcus sobrinus* | TCI-363 |
| 3781 | 60062 | Not found | *Streptococcus sobrinus* | TCI-366 |
| 3782 | 60063 | Not found | *Streptococcus sobrinus* | TCI-367 |
| 3783 | 60064 | Not found | *Streptococcus sobrinus* | TCI-373 |
| 3784 | 60067 | Not found | *Streptococcus sobrinus* | TCI-377 |
| 3785 | 60068 | Not found | *Streptococcus sobrinus* | TCI-381 |
| 3786 | 60069 | Not found | *Streptococcus sobrinus* | TCI-384 |
| 3787 | 60070 | Not found | *Streptococcus sobrinus* | TCI-392 |
| 3788 | 60071 | Not found | *Streptococcus sobrinus* | TCI-395 |
| 3789 | 60072 | Not found | *Streptococcus sobrinus* | TCI-396 |
| 3790 | 60073 | Not found | *Streptococcus sobrinus* | TCI-50 |
| 3791 | 60074 | Not found | *Streptococcus sobrinus* | TCI-53 |
| 3792 | 60075 | Not found | *Streptococcus sobrinus* | TCI-54 |
| 3793 | 60076 | Not found | *Streptococcus sobrinus* | TCI-56 |
| 3794 | 60077 | Not found | *Streptococcus sobrinus* | TCI-61 |
| 3795 | 60078 | Not found | *Streptococcus sobrinus* | TCI-77 |
| 3796 | 60079 | Not found | *Streptococcus sobrinus* | TCI-79 |
| 3797 | 60080 | Not found | *Streptococcus sobrinus* | TCI-80 |
| 3798 | 60081 | Not found | *Streptococcus sobrinus* | TCI-89 |
| 3799 | 60082 | Not found | *Streptococcus sobrinus* | TCI-9 |
| 3800 | 60083 | Not found | *Streptococcus sobrinus* | TCI-98 |
| 3801 | 60084 | Not found | *Streptococcus sobrinus* | W1703 |
| 3802 | 60085 | Not found | *Streptococcus thoraltensis* | DSM 12221 |
| 3803 | 60086 | Not found | *Streptococcus oralis* | 1366 |
| 3804 | 60088 | Not found | *Streptococcus oralis* | 2426 |
| 3805 | 60089 | Not found | *Streptococcus oralis* | AZ_3a |
| 3806 | 60090 | Not found | *Streptococcus urinalis* | 2285-97 |
| 3807 | 60091 | Not found | *Streptococcus urinalis* | FB127-CNA-2 |
| 3808 | 60092 | Not found | *Streptococcus vestibularis* | ATCC 49124 |
| 3809 | 60093 | Not found | *Streptococcus vestibularis* | F0396 |
| 3810 | 63661 | Not found | *Streptococcus criceti* | SRR067590 |
| 3811 | 63662 | Not found | *Streptococcus ictaluri* | SRR067674 |
| 3812 | 63663 | Not found | *Streptococcus macacae* | SRR067596 |
| 3813 | 63664 | 1 | *Streptococcus pseudopneumoniae* | SRR387783 |
| 3814 | 63665 | 1 | *Streptococcus pseudopneumoniae* | SRR387784 |
| 3815 | 63666 | 1 | *Streptococcus pseudopneumoniae* | SRR387648 |
| 3816 | 63667 | Not found | *Streptococcus sanguinis* | SRR959141 |
| 3817 | 63668 | Not found | *Streptococcus sanguinis* | SRR835814 |
| 3818 | 63669 | Not found | *Streptococcus urinalis* | SRR536860 |
| 3819 | 63670 | Not found | *Streptococcus urinalis* | SRR067581 |
| 3820 | 63987 | Not found | *Streptococcus anginosus* | SRR088899 |
| 3821 | 63988 | Not found | *Streptococcus anginosus* | SRR387639 |
| 3822 | 63989 | Not found | *Streptococcus anginosus* | SRR387641 |
| 3823 | 63990 | Not found | *Streptococcus anginosus* | SRR446556 |
| 3824 | 63991 | Not found | *Streptococcus constellatus* | SRR387643 |
| 3825 | 63992 | Not found | *Streptococcus constellatus* | SRR387644 |
| 3826 | 63993 | Not found | *Streptococcus intermedius* | SRR387763 |
| 3827 | 63994 | Not found | *Streptococcus intermedius* | SRR387765 |
| 3828 | 63995 | Not found | *Streptococcus oralis* | ERR097399 |
| 3829 | 63996 | Not found | *Streptococcus oralis* | ERR097400 |
| 3830 | 63997 | Not found | *Streptococcus oralis* | ERR097401 |
| 3831 | 63998 | Not found | *Streptococcus oralis* | SRR387785 |
| 3832 | 63999 | Not found | *Streptococcus oralis* | SRR387786 |
| 3833 | 64001 | Not found | *Streptococcus oralis* | SRR387788 |
| 3834 | 64002 | Not found | *Streptococcus parasanguinis* | SRR387738 |
| 3835 | 64003 | Not found | *Streptococcus parasanguinis* | SRR387741 |
| 3836 | 64004 | Not found | *Streptococcus parasanguinis* | SRR835813 |
| 3837 | 64005 | Not found | *Streptococcus parasanguinis* | SRR959135 |
| 3838 | 64006 | Not found | *Streptococcus parauberis* | SRR067675 |
| 3839 | 65006 | Not found | *Streptococcus porcinus* | SRR067676 |
| 3840 | 65007 | Not found | *Streptococcus pseudoporcinus* | SRR067677 |
| 3841 | 65008 | Not found | *Streptococcus sanguinis* | ERR245866 |
| 3842 | 65182 | 2 | *Streptococcus agalactiae* | ERR054983 |
| 3843 | 65183 | 2 | *Streptococcus agalactiae* | ERR054985 |
| 3844 | 65208 | 2 | *Streptococcus agalactiae* | ERR054988 |
| 3845 | 65223 | 2 | *Streptococcus agalactiae* | ERR054992 |
| 3846 | 65249 | 2 | *Streptococcus agalactiae* | ERR126775 |
| 3847 | 65401 | 2 | *Streptococcus agalactiae* | ERR126788 |
| 3848 | 65434 | 2 | *Streptococcus agalactiae* | ERR126795 |
| 3849 | 65463 | 2 | *Streptococcus agalactiae* | ERR126810 |
| 3850 | 65500 | 2 | *Streptococcus agalactiae* | ERR126821 |
| 3851 | 65522 | 2 | *Streptococcus agalactiae* | ERR126832 |
| 3852 | 65666 | 2 | *Streptococcus agalactiae* | ERR129758 |
| 3853 | 65804 | 2 | *Streptococcus agalactiae* | ERR129800 |
| 3854 | 65843 | 2 | *Streptococcus agalactiae* | SRR628717 |
| 3855 | 65850 | Not found | *Streptococcus dysgalactiae* | ERR084975 |
| 3856 | 65852 | Not found | *Streptococcus dysgalactiae* | ERR084977 |
| 3857 | 65856 | Not found | *Streptococcus dysgalactiae* | ERR084979 |
| 3858 | 65859 | Not found | *Streptococcus dysgalactiae* | ERR084982 |
| 3859 | 65890 | Not found | *Streptococcus dysgalactiae* | ERR084988 |
| 3860 | 65905 | Not found | *Streptococcus dysgalactiae* | ERR084990 |
| 3861 | 65935 | Not found | *Streptococcus dysgalactiae* | ERR084991 |
| 3862 | 65937 | Not found | *Streptococcus dysgalactiae* | ERR084992 |
| 3863 | 66021 | Not found | *Streptococcus dysgalactiae* | ERR085000 |
| 3864 | 66037 | Not found | *Streptococcus dysgalactiae* | ERR085005 |
| 3865 | 66038 | Not found | *Streptococcus dysgalactiae* | ERR085006 |
| 3866 | 66039 | Not found | *Streptococcus dysgalactiae* | ERR085007 |
| 3867 | 66040 | Not found | *Streptococcus dysgalactiae* | ERR085008 |
| 3868 | 66045 | Not found | *Streptococcus dysgalactiae* | ERR085015 |
| 3869 | 66050 | Not found | *Streptococcus dysgalactiae* | ERR085021 |
| 3870 | 66052 | Not found | *Streptococcus dysgalactiae* | ERR085025 |
| 3871 | 66054 | Not found | *Streptococcus dysgalactiae* | ERR085028 |
| 3872 | 66056 | Not found | *Streptococcus dysgalactiae* | ERR085030 |
| 3873 | 66057 | Not found | *Streptococcus dysgalactiae* | ERR085032 |
| 3874 | 66067 | Not found | *Streptococcus dysgalactiae* | ERR085045 |
| 3875 | 66069 | Not found | *Streptococcus dysgalactiae* | ERR085054 |
| 3876 | 66072 | Not found | *Streptococcus dysgalactiae* | ERR085057 |
| 3877 | 66073 | Not found | *Streptococcus dysgalactiae* | ERR085058 |
| 3878 | 66074 | Not found | *Streptococcus dysgalactiae* | ERR085059 |
| 3879 | 66076 | Not found | *Streptococcus dysgalactiae* | ERR085061 |
| 3880 | 66077 | Not found | *Streptococcus dysgalactiae* | ERR085062 |
| 3881 | 66079 | Not found | *Streptococcus dysgalactiae* | ERR085067 |
| 3882 | 66080 | Not found | *Streptococcus dysgalactiae* | ERR085068 |
| 3883 | 66082 | Not found | *Streptococcus dysgalactiae* | ERR109289 |
| 3884 | 66085 | Not found | *Streptococcus dysgalactiae* | ERR109306 |
| 3885 | 66086 | Not found | *Streptococcus dysgalactiae* | ERR109308 |
| 3886 | 66089 | Not found | *Streptococcus dysgalactiae* | ERR109312 |
| 3887 | 66091 | Not found | *Streptococcus dysgalactiae* | ERR109355 |
| 3888 | 66093 | Not found | *Streptococcus dysgalactiae* | ERR109361 |
| 3889 | 66095 | Not found | *Streptococcus dysgalactiae* | ERR109370 |
| 3890 | 66097 | Not found | *Streptococcus dysgalactiae* | ERR111982 |
| 3891 | 66099 | Not found | *Streptococcus equi* | ERR045925 |
| 3892 | 66103 | Not found | *Streptococcus equi* | ERR045954 |
| 3893 | 66105 | Not found | *Streptococcus equi* | ERR066392 |
| 3894 | 66106 | Not found | *Streptococcus equi* | ERR066394 |
| 3895 | 66107 | Not found | *Streptococcus equi* | ERR066395 |
| 3896 | 66108 | Not found | *Streptococcus equi* | ERR066396 |
| 3897 | 66119 | Not found | *Streptococcus equi* | ERR066407 |
| 3898 | 66125 | Not found | *Streptococcus equi* | ERR066420 |
| 3899 | 66135 | Not found | *Streptococcus equi* | ERR066421 |
| 3900 | 66149 | Not found | *Streptococcus equi* | ERR066431 |
| 3901 | 66161 | Not found | *Streptococcus equi* | ERR066435 |
| 3902 | 66168 | Not found | *Streptococcus equi* | ERR066439 |
| 3903 | 66369 | Not found | *Streptococcus equi* | ERR066524 |
| 3904 | 66372 | Not found | *Streptococcus equi* | ERR066528 |
| 3905 | 66384 | Not found | *Streptococcus equi* | ERR066538 |
| 3906 | 66385 | Not found | *Streptococcus equi* | ERR066541 |
| 3907 | 66402 | Not found | *Streptococcus equi* | ERR066561 |
| 3908 | 66407 | Not found | *Streptococcus equi* | ERR066565 |
| 3909 | 66412 | Not found | *Streptococcus equi* | ERR066568 |
| 3910 | 66416 | Not found | *Streptococcus equi* | ERR066572 |
| 3911 | 66417 | Not found | *Streptococcus equi* | ERR066573 |
| 3912 | 66433 | Not found | *Streptococcus equi* | ERR066580 |
| 3913 | 66437 | Not found | *Streptococcus mutans* | ERR204000 |
| 3914 | 66461 | Not found | *Streptococcus mutans* | ERR204007 |
| 3915 | 66491 | Not found | *Streptococcus mutans* | ERR204012 |
| 3916 | 66499 | Not found | *Streptococcus mutans* | ERR204016 |
| 3917 | 66576 | Not found | *Streptococcus mutans* | ERR298628 |
| 3918 | 66579 | Not found | *Streptococcus mutans* | ERR298636 |
| 3919 | 66582 | Not found | *Streptococcus mutans* | ERR298644 |
| 3920 | 66583 | Not found | *Streptococcus mutans* | ERR298645 |
| 3921 | 66587 | Not found | *Streptococcus mutans* | ERR298660 |
| 3922 | 66588 | Not found | *Streptococcus mutans* | ERR298661 |
| 3923 | 66593 | Not found | *Streptococcus mutans* | ERR298683 |
| 3924 | 66597 | Not found | *Streptococcus mutans* | ERR298691 |
| 3925 | 66603 | Not found | *Streptococcus mutans* | ERR298701 |
| 3926 | 66616 | Not found | *Streptococcus suis* | ERR120031 |
| 3927 | 66619 | Not found | *Streptococcus suis* | ERR120038 |
| 3928 | 66626 | Not found | *Streptococcus suis* | ERR120064 |
| 3929 | 66643 | Not found | *Streptococcus suis* | ERR120115 |
| 3930 | 66644 | Not found | *Streptococcus suis* | ERR120116 |
| 3931 | 66661 | Not found | *Streptococcus suis* | ERR120214 |
| 3932 | 66696 | Not found | *Streptococcus suis* | ERR211792 |
| 3933 | 68042 | Not found | *Streptococcus anginosus* | SK52 |
| 3934 | 68104 | 2 | *Streptococcus agalactiae* | 09mas018883 |
| 3935 | 68105 | 2 | *Streptococcus agalactiae* | GD201008-001 |
| 3936 | 68106 | 2 (interrupted by MGE) | *Streptococcus agalactiae* | ILRI005 |
| 3937 | 68107 | 2 | *Streptococcus agalactiae* | ILRI112 |
| 3938 | 68108 | 2 | *Streptococcus agalactiae* | SA20-06 |
| 3939 | 68111 | Not found | *Streptococcus dysgalactiae* | equisimilis AC-2713 |
| 3940 | 68114 | Not found | *Streptococcus equi* | ATCC 35246 |
| 3941 | 68115 | 7 | *Streptococcus gallolyticus* | ATCC 43143 |
| 3942 | 68116 | 7 | *Streptococcus gallolyticus* | ATCC BAA-2069 |
| 3943 | 68117 | 5 | *Streptococcus infantarius* | infantarius CJ18 |
| 3944 | 68118 | Not found | *Streptococcus mutans* | GS-5 |
| 3945 | 68119 | Not found | *Streptococcus mutans* | LJ23 |
| 3946 | 68137 | Not found | *Streptococcus pyogenes* | Alab49 |
| 3947 | 68138 | Not found | *Streptococcus pyogenes* | HSC5 |
| 3948 | 68140 | Not found | *Streptococcus salivarius* | 57.I |
| 3949 | 68142 | Not found | *Streptococcus salivarius* | JIM8777 |
| 3950 | 68149 | Not found | *Streptococcus suis* | A7 |
| 3951 | 68150 | Not found | *Streptococcus suis* | D12 PRJNA65469 |
| 3952 | 68151 | Not found | *Streptococcus suis* | D9 PRJNA65435 |
| 3953 | 68157 | Not found | *Streptococcus suis* | ST1 |
| 3954 | 68158 | Not found | *Streptococcus suis* | ST3 |
| 3955 | 68159 | Not found | *Streptococcus suis* | T15 |
| 3956 | 68160 | Not found | *Streptococcus suis* | TL13 |
| 3957 | 68161 | Not found | *Streptococcus suis* | YB51 |
| 3958 | 68162 | Not found | *Streptococcus thermophilus* | JIM 8232 |
| 3959 | 68163 | Not found | *Streptococcus thermophilus* | MN-ZLW-002 |
| 3960 | 68164 | Not found | *Streptococcus thermophilus* | ND03 |
| 3961 | 68969 | Not found | *Streptococcus castoreus* | DSM 17536 |
| 3962 | 68970 | Not found | *Streptococcus oralis* | 7746 PRJEB1503 |
| 3963 | 68971 | Not found | *Streptococcus oralis* | 7747 PRJEB1510 |
| 3964 | 68972 | 10 | *Streptococcus devriesei* | DSM 19639 |
| 3965 | 68973 | Not found | *Streptococcus hyovaginalis* | DSM 12219 |
| 3966 | 68974 | Not found | *Streptococcus plurextorum* | DSM 22810 |
| 3967 | 68975 | Not found | *Streptococcus porci* | DSM 23759 |
| 3969 | 72276 | 2 | *Streptococcus agalactiae* | BSU165 |
| 3970 | 72282 | 2 | *Streptococcus agalactiae* | BSU248 |
| 3971 | 72288 | 2 | *Streptococcus agalactiae* | BSU450 |
| 3972 | 72289 | 2 | *Streptococcus agalactiae* | BSU451 |
| 3973 | 72294 | 2 | *Streptococcus agalactiae* | CCUG 17336 |
| 3974 | 72301 | 2 | *Streptococcus agalactiae* | CCUG 30636 |
| 3975 | 72304 | 2 | *Streptococcus agalactiae* | CCUG 37736 |
| 3976 | 72312 | 2 | *Streptococcus agalactiae* | CCUG 39096 A |
| 3977 | 72364 | 2 | *Streptococcus agalactiae* | GB00082 |
| 3978 | 72387 | 2 | *Streptococcus agalactiae* | GB00588 |
| 3979 | 72390 | 2 | *Streptococcus agalactiae* | GB00640 |
| 3980 | 72408 | 2 | *Streptococcus agalactiae* | GB00901 |
| 3981 | 72413 | 2 | *Streptococcus agalactiae* | GB00922 |
| 3982 | 72424 | 2 | *Streptococcus agalactiae* | GB00959 |
| 3983 | 72438 | 2 | *Streptococcus agalactiae* | LADL-90-503 |
| 3984 | 72513 | 2 | *Streptococcus agalactiae* | SGBS026 |
| 3985 | 72515 | 2 | *Streptococcus agalactiae* | SGBS029 |
| 3986 | 72535 | 2 | *Streptococcus agalactiae* | Gottschalk 1002A |
| 3987 | 72545 | Not found | *Streptococcus anginosus* | 1_2_62CV |
| 3988 | 72546 | Not found | *Streptococcus anginosus* | 1505 |
| 3989 | 72547 | Not found | *Streptococcus anginosus* | DORA_7 |
| 3990 | 72548 | Not found | *Streptococcus anginosus* | T5 |
| 3991 | 72551 | Not found | *Streptococcus equi* | zooepidemicus BHS5 |
| 3992 | 72553 | 3 | *Streptococcus equinus* | B315 |
| 3993 | 72554 | 3 | *Streptococcus equinus* | SN033 |
| 3994 | 72555 | 7 | *Streptococcus gallolyticus* | gallolyticus TX20005 |
| 3995 | 72556 | Not found | *Streptococcus iniae* | 9117 |
| 3996 | 72557 | Not found | *Streptococcus iniae* | IUSA1 |
| 3997 | 72558 | Not found | *Streptococcus iniae* | KCTC 11634BP |
| 3998 | 72559 | Not found | *Streptococcus intermedius* | ATCC 27335 |
| 3999 | 72560 | Not found | *Streptococcus intermedius* | BA1 |
| 4000 | 72562 | Not found | *Streptococcus intermedius* | F0413 |
| 4001 | 72563 | Not found | *Streptococcus massiliensis* | 4401825 |
| 4002 | 72564 | Not found | *Streptococcus mitis* | 11/5 |
| 4003 | 72565 | Not found | *Streptococcus mitis* | 13/39 |
| 4004 | 72566 | Not found | *Streptococcus mitis* | 17/34 |
| 4005 | 72567 | Not found | *Streptococcus mitis* | 18/56 |
| 4006 | 72568 | Not found | *Streptococcus mitis* | 21/39 |
| 4007 | 72569 | Not found | *Streptococcus mitis* | 27/7 |
| 4008 | 72576 | Not found | *Streptococcus mitis* | SK1080 |
| 4009 | 72577 | Not found | *Streptococcus mitis* | SK321 |
| 4010 | 72578 | Not found | *Streptococcus mitis* | SK564 |
| 4011 | 72579 | Not found | *Streptococcus mitis* | SK569 |
| 4012 | 72580 | Not found | *Streptococcus mitis* | SK575 |
| 4013 | 72583 | Not found | *Streptococcus mitis* | SK616 |
| 4014 | 72586 | Not found | *Streptococcus mutans* | 11SSST2 |
| 4015 | 72588 | Not found | *Streptococcus mutans* | 14D |
| 4016 | 72593 | Not found | *Streptococcus mutans* | 21 |
| 4017 | 72597 | Not found | *Streptococcus mutans* | 3SN1 |
| 4018 | 72604 | Not found | *Streptococcus mutans* | A19 |
| 4019 | 72608 | Not found | *Streptococcus mutans* | AHTB01 |
| 4020 | 72634 | Not found | *Streptococcus mutans* | M2A |
| 4021 | 72635 | Not found | *Streptococcus mutans* | N29 |
| 4022 | 72636 | Not found | *Streptococcus mutans* | N3209 |
| 4023 | 72638 | Not found | *Streptococcus mutans* | N66 |
| 4024 | 72639 | Not found | *Streptococcus mutans* | NCTC 11060 |
| 4025 | 72642 | Not found | *Streptococcus mutans* | NLML1 |
| 4026 | 72653 | Not found | *Streptococcus mutans* | R221 |
| 4027 | 72665 | Not found | *Streptococcus mutans* | str. B16 P Sm1 |
| 4028 | 72666 | Not found | *Streptococcus mutans* | T4 |
| 4029 | 72667 | Not found | *Streptococcus mutans* | TCI-101 |
| 4030 | 72668 | Not found | *Streptococcus mutans* | TCI-109 |
| 4031 | 72671 | Not found | *Streptococcus mutans* | TCI-116 |
| 4032 | 72680 | Not found | *Streptococcus mutans* | TCI-149 |
| 4033 | 72683 | Not found | *Streptococcus mutans* | TCI-153 |
| 4034 | 72687 | Not found | *Streptococcus mutans* | TCI-164 |
| 4035 | 72689 | Not found | *Streptococcus mutans* | TCI-170 |
| 4036 | 72693 | Not found | *Streptococcus mutans* | TCI-179 |
| 4037 | 72694 | Not found | *Streptococcus mutans* | TCI-187 |
| 4038 | 72702 | Not found | *Streptococcus mutans* | TCI-218 |
| 4039 | 72711 | Not found | *Streptococcus mutans* | TCI-239 |
| 4040 | 72714 | Not found | *Streptococcus mutans* | TCI-244 |
| 4041 | 72715 | Not found | *Streptococcus mutans* | TCI-249 |
| 4042 | 72719 | Not found | *Streptococcus mutans* | TCI-267 |
| 4043 | 72730 | Not found | *Streptococcus mutans* | TCI-400 |
| 4044 | 72733 | Not found | *Streptococcus mutans* | TCI-70 |
| 4045 | 72735 | Not found | *Streptococcus mutans* | TCI-78 |
| 4046 | 72741 | Not found | *Streptococcus mutans* | TCI-99 |
| 4047 | 72746 | Not found | *Streptococcus oralis* | ATCC 49296 |
| 4048 | 72747 | Not found | *Streptococcus oralis* | SK1074 |
| 4049 | 72748 | Not found | *Streptococcus oralis* | SK255 |
| 4050 | 72749 | Not found | *Streptococcus oralis* | SK304 |
| 4051 | 72750 | 1 | *Streptococcus oralis* | SK313 |
| 4052 | 72751 | Not found | *Streptococcus parasanguinis* | ATCC 903 |
| 4053 | 72753 | Not found | *Streptococcus parasanguinis* | F0405 |
| 4054 | 72754 | Not found | *Streptococcus parasanguinis* | SK236 |
| 4055 | 72755 | Not found | *Streptococcus parauberis* | KCTC 11980BP |
| 4056 | 72756 | Not found | *Streptococcus parauberis* | KRS-02083 |
| 4057 | 72757 | Not found | *Streptococcus parauberis* | KRS-02109 |
| 4058 | 72758 | Not found | *Streptococcus parauberis* | NCFD 2020 |
| 4059 | 73004 | 1 | *Streptococcus pseudopneumoniae* | 1321 |
| 4060 | 73005 | 1 | *Streptococcus pseudopneumoniae* | 22725 |
| 4061 | 73006 | 1 | *Streptococcus pseudopneumoniae* | 5247 |
| 4062 | 73010 | Not found | *Streptococcus pyogenes* | BJCYGAS15 |
| 4063 | 73014 | Not found | *Streptococcus pyogenes* | GA03799 |
| 4064 | 73037 | Not found | *Streptococcus salivarius* | K12 |
| 4065 | 73038 | Not found | *Streptococcus salivarius* | M18 |
| 4066 | 73040 | Not found | *Streptococcus sanguinis* | ATCC 29667 |
| 4067 | 73041 | Not found | *Streptococcus sanguinis* | SK1 |
| 4068 | 73042 | Not found | *Streptococcus sanguinis* | SK1056 |
| 4069 | 73043 | Not found | *Streptococcus sanguinis* | SK1057 |
| 4070 | 73044 | Not found | *Streptococcus sanguinis* | SK1058 |
| 4071 | 73045 | Not found | *Streptococcus sanguinis* | SK1059 |
| 4072 | 73046 | Not found | *Streptococcus sanguinis* | SK1087 |
| 4073 | 73047 | Not found | *Streptococcus sanguinis* | SK115 |
| 4074 | 73048 | Not found | *Streptococcus sanguinis* | SK150 |
| 4075 | 73049 | Not found | *Streptococcus sanguinis* | SK160 |
| 4076 | 73050 | Not found | *Streptococcus sanguinis* | SK330 |
| 4077 | 73051 | Not found | *Streptococcus sanguinis* | SK340 |
| 4078 | 73052 | Not found | *Streptococcus sanguinis* | SK353 |
| 4079 | 73053 | Not found | *Streptococcus sanguinis* | SK355 |
| 4080 | 73054 | Not found | *Streptococcus sanguinis* | SK405 |
| 4081 | 73055 | Not found | *Streptococcus sanguinis* | SK408 |
| 4082 | 73056 | Not found | *Streptococcus sanguinis* | SK49 |
| 4083 | 73057 | Not found | *Streptococcus sanguinis* | SK678 |
| 4084 | 73058 | Not found | *Streptococcus sanguinis* | SK72 |
| 4085 | 73059 | Not found | *Streptococcus sanguinis* | VMC66 |
| 4086 | 73062 | Not found | *Streptococcus sobrinus* | TCI-175 |
| 4087 | 73083 | Not found | *Streptococcus suis* | 86-5192 |
| 4088 | 73089 | Not found | *Streptococcus suis* | 89-3576-3 |
| 4089 | 73111 | Not found | *Streptococcus suis* | YS23 |
| 4090 | 73113 | Not found | *Streptococcus suis* | YS27 |
| 4091 | 73114 | Not found | *Streptococcus suis* | YS3 |
| 4092 | 73118 | Not found | *Streptococcus suis* | YS39 |
| 4093 | 73123 | Not found | *Streptococcus suis* | YS49 |
| 4094 | 73126 | Not found | *Streptococcus suis* | YS54 |
| 4095 | 73128 | Not found | *Streptococcus suis* | YS57 |
| 4096 | 73139 | Not found | *Streptococcus thermophilus* | MTCC 5460 |
| 4097 | 73140 | Not found | *Streptococcus thermophilus* | MTCC 5461 |
| 4098 | 79985 | Not found | *Streptococcus pyogenes* | ERR374654 |
| 4099 | 83118 | Not found | *Streptococcus pyogenes* | ERR448936 |
| 4100 | 83119 | Not found | *Streptococcus pyogenes* | ERR448939 |
| 4101 | 83126 | Not found | *Streptococcus pyogenes* | ERR473496 |
| 4102 | 83128 | Not found | *Streptococcus pyogenes* | ERR473499 |
| 4103 | 83134 | Not found | *Streptococcus pyogenes* | ERR473528 |
| 4104 | 83137 | Not found | *Streptococcus pyogenes* | ERR473564 |
| 4105 | 83157 | Not found | *Streptococcus pyogenes* | ERR473676 |
| 4106 | 83168 | Not found | *Streptococcus pyogenes* | ERR485792 |
| 4107 | 83169 | Not found | *Streptococcus pyogenes* | ERR485801 |
| 4108 | 83193 | Not found | *Streptococcus pyogenes* | SRR1169305 |
| 4109 | 84553 | Not found | *Streptococcus pyogenes* | ERR448962 |
| 4110 | 84557 | Not found | *Streptococcus pyogenes* | ERR473452 |
| 4111 | 84570 | Not found | *Streptococcus pyogenes* | ERR473474 |
| 4112 | 84596 | Not found | *Streptococcus pyogenes* | ERR473551 |
| 4113 | 93529 | 2 | *Streptococcus agalactiae* | GB00111 |
| 4114 | 93537 | Not found | *Streptococcus anginosus* | CCUG 39159 |
| 4115 | 93538 | Not found | *Streptococcus anginosus* | F0211 |
| 4116 | 93539 | Not found | *Streptococcus anginosus* | SK1138 |
| 4117 | 93540 | Not found | *Streptococcus constellatus* | SK53 |
| 4118 | 93541 | Not found | *Streptococcus intermedius* | SK54 |
| 4119 | 93545 | Not found | *Streptococcus parasanguinis* | CC87K |
| 4120 | 93546 | Not found | *Streptococcus parasanguinis* | F0449 |
| 4121 | 93548 | 1 | *Streptococcus pseudopneumoniae* | ATCC BAA-960 |
| 4122 | 93549 | 1 | *Streptococcus pseudopneumoniae* | SK674 |
| 4123 | 93550 | Not found | *Streptococcus sanguinis* | CC94A |
| 4124 | 93584 | Not found | *Streptococcus thermophilus* | DGCC7710 |
| 4125 | 109311 | Not found | *Streptococcus anginosus* | SK52 AREF01 |
| 4126 | 109312 | Not found | *Streptococcus anginosus* | SK52 BAST01 |
| 4127 | 109313 | Not found | *Streptococcus constellatus* | SK1060 |
| 4128 | 109314 | Not found | *Streptococcus equi* | CECT 5772 |
| 4129 | 109315 | 3 | *Streptococcus equinus* | 2B |
| 4130 | 109316 | 3 | *Streptococcus equinus* | ATCC 33317 |
| 4131 | 109317 | 3 | *Streptococcus equinus* | HC5 |
| 4132 | 109318 | 7 | *Streptococcus gallolyticus* | LMG17956 |
| 4133 | 109319 | Not found | *Streptococcus macedonicus* | 33MO |
| 4134 | 109320 | Not found | *Streptococcus mitis* | SK1126 |
| 4135 | 109321 | Not found | *Streptococcus mitis* | SK271 |
| 4136 | 109323 | Not found | *Streptococcus mitis* | SK608 |
| 4137 | 109325 | Not found | *Streptococcus mitis* | SK637 |
| 4138 | 109326 | Not found | *Streptococcus mitis* | SK642 |
| 4139 | 109327 | Not found | *Streptococcus mitis* | SK667 |
| 4140 | 109328 | Not found | *Streptococcus oralis* | SK143 |
| 4141 | 109329 | Not found | *Streptococcus parasanguinis* | C1A |
| 4142 | 109330 | Not found | *Streptococcus parauberis* | SK-417 |
| 4143 | 109419 | 9 | *Streptococcus ratti* | DSM 20564 |
| 4144 | 109420 | Not found | *Streptococcus salivarius* | NU10 |
| 4145 | 109421 | Not found | *Streptococcus salivarius* | YU10 |
| 4146 | 109422 | Not found | *Streptococcus sobrinus* | DSM 20742 |
| 4148 | 109426 | Not found | *Streptococcus thermophilus* | MTH17CL396 |
| 4149 | 109427 | Not found | *Streptococcus thermophilus* | TH1435 |
| 4150 | 109428 | Not found | *Streptococcus thermophilus* | TH1436 |
| 4151 | 110327 | 1 | *Streptococcus mitis* | ERR097396 |
| 4152 | 110328 | Not found | *Streptococcus mitis* | ERR097397 |
| 4153 | 110329 | 1 | *Streptococcus pseudopneumoniae* | ERR097393 |
| 4154 | 110330 | 1 | *Streptococcus pseudopneumoniae* | ERR097395 |
| 4155 | 110620 | Not found | *Streptococcus canis* | ERR438797 |
| 4156 | 110621 | Not found | *Streptococcus canis* | ERR438798 |
| 4157 | 110622 | Not found | *Streptococcus canis* | ERR438806 |
| 4158 | 110623 | Not found | *Streptococcus canis* | ERR438807 |
| 4159 | 110624 | Not found | *Streptococcus canis* | ERR438800 |
| 4160 | 110625 | Not found | *Streptococcus canis* | ERR438805 |
| 4161 | 110626 | Not found | *Streptococcus canis* | ERR438803 |
| 4162 | 110627 | Not found | *Streptococcus canis* | ERR438804 |
| 4163 | 110628 | Not found | *Streptococcus canis* | ERR438802 |
| 4164 | 110629 | Not found | *Streptococcus canis* | ERR438801 |
| 4165 | 110632 | Not found | *Streptococcus dysgalactiae* | ERR085022 |
| 4166 | 110633 | Not found | *Streptococcus dysgalactiae* | ERR085010 |
| 4167 | 110634 | Not found | *Streptococcus dysgalactiae* | ERR085023 |
| 4168 | 110639 | Not found | *Streptococcus equi* | ERR045911 |
| 4169 | 110641 | Not found | *Streptococcus equi* | ERR045915 |
| 4170 | 110642 | Not found | *Streptococcus equi* | ERR045909 |
| 4171 | 110644 | Not found | *Streptococcus equi* | ERR045914 |
| 4172 | 110645 | Not found | *Streptococcus equi* | ERR045908 |
| 4173 | 110646 | Not found | *Streptococcus equi* | ERR045907 |
| 4174 | 110647 | Not found | *Streptococcus equi* | ERR045910 |
| 4175 | 110648 | Not found | *Streptococcus equi* | ERR045912 |
| 4176 | 154723 | 2 | *Streptococcus agalactiae* | BE-PW-130 |
| 4177 | 154761 | 2 | *Streptococcus agalactiae* | DE-NI-010 |
| 4178 | 154772 | 2 | *Streptococcus agalactiae* | DE-NI-041 |
| 4179 | 154833 | 2 | *Streptococcus agalactiae* | ES-PW-160 |
| 4180 | 154861 | 2 | *Streptococcus agalactiae* | GB-PW-094 |
| 4181 | 154869 | 2 | *Streptococcus agalactiae* | IT-NI-007 |
| 4182 | 154922 | Not found | *Streptococcus anginosus* | 1080_SANG |
| 4183 | 154923 | Not found | *Streptococcus anginosus* | 557_SANG |
| 4184 | 154925 | Not found | *Streptococcus anginosus* | 83_SANG |
| 4185 | 154927 | Not found | *Streptococcus constellatus* | 925_SCON |
| 4186 | 154928 | Not found | *Streptococcus constellatus* | KCOM 1650 |
| 4187 | 154929 | Not found | *Streptococcus cristatus* | CC5A |
| 4188 | 154930 | Not found | *Streptococcus cristatus* | CR3 |
| 4189 | 154933 | Not found | *Streptococcus dysgalactiae* | UT-5345 |
| 4190 | 154934 | Not found | *Streptococcus dysgalactiae* | UT-5354 |
| 4191 | 154935 | Not found | *Streptococcus dysgalactiae* | UT-SS1069 |
| 4192 | 154937 | Not found | *Streptococcus dysgalactiae* | WCHSDSE-1 |
| 4193 | 154942 | Not found | *Streptococcus equi* | EQUI0005 |
| 4194 | 154944 | Not found | *Streptococcus equi* | EQUI0007 |
| 4195 | 154959 | Not found | *Streptococcus equi* | EQUI0022 |
| 4196 | 154976 | Not found | *Streptococcus equi* | EQUI0039 |
| 4197 | 155080 | Not found | *Streptococcus equi* | EQUI0155 |
| 4198 | 155162 | Not found | *Streptococcus equi* | Sz105 |
| 4199 | 155164 | Not found | *Streptococcus equi* | Sz16 |
| 4200 | 155165 | Not found | *Streptococcus equi* | Sz35 |
| 4201 | 155167 | Not found | *Streptococcus equi* | Sz5 |
| 4202 | 155168 | Not found | *Streptococcus equi* | Sz57 |
| 4203 | 155169 | Not found | *Streptococcus equi* | SzAM35 |
| 4204 | 155171 | 3 | *Streptococcus equinus* | AG46 |
| 4205 | 155172 | 7 | *Streptococcus gallolyticus* | NTS 31106099 |
| 4206 | 155173 | Not found | *Streptococcus gordonii* | 1116_SGOR |
| 4207 | 155174 | Not found | *Streptococcus gordonii* | G9B |
| 4208 | 155175 | Not found | *Streptococcus gordonii* | IE35 |
| 4209 | 155178 | Not found | *Streptococcus mitis* | 1111_SMIT |
| 4210 | 155185 | Not found | *Streptococcus mitis* | SK137 |
| 4211 | 155202 | Not found | *Streptococcus oralis* | 727_SORA |
| 4212 | 155203 | Not found | *Streptococcus oralis* | 734_SORA |
| 4213 | 155204 | Not found | *Streptococcus oralis* | 89a |
| 4214 | 155206 | Not found | *Streptococcus oralis* | 918_SORA |
| 4215 | 155207 | Not found | *Streptococcus orisasini* | SH06 |
| 4216 | 155208 | Not found | *Streptococcus parasanguinis* | 1287_SPAR |
| 4217 | 155209 | Not found | *Streptococcus parasanguinis* | 139.rep1_SPAR |
| 4218 | 155210 | Not found | *Streptococcus parasanguinis* | 139.rep2_SPAR |
| 4219 | 155211 | Not found | *Streptococcus parasanguinis* | 318_SPAR |
| 4220 | 155212 | Not found | *Streptococcus parasanguinis* | 344_SPAR |
| 4221 | 155214 | Not found | *Streptococcus parasanguinis* | 349_SPAR |
| 4222 | 155215 | Not found | *Streptococcus parasanguinis* | 392_SPAR |
| 4223 | 155216 | Not found | *Streptococcus parasanguinis* | 451_SPAR |
| 4224 | 155217 | Not found | *Streptococcus parasanguinis* | 512_SPAR |
| 4225 | 155218 | Not found | *Streptococcus parasanguinis* | 540.rep1_SPAR |
| 4226 | 155219 | Not found | *Streptococcus parasanguinis* | 540.rep2_SPAR |
| 4227 | 155220 | Not found | *Streptococcus parasanguinis* | 65_SPAR |
| 4228 | 155221 | Not found | *Streptococcus parasanguinis* | 766_SPAR |
| 4229 | 155222 | Not found | *Streptococcus parasanguinis* | 886_SPAR |
| 4230 | 155223 | Not found | *Streptococcus parasanguinis* | 889_SPAR |
| 4231 | 155224 | Not found | *Streptococcus parasanguinis* | MGH413 |
| 4232 | 155225 | 7 | *Streptococcus pasteurianus* | 651_SPAS |
| 4233 | 155226 | 7 | *Streptococcus pasteurianus* | HC-2909-2 |
| 4234 | 155227 | Not found | *Streptococcus phocae* | ATCC 51973 |
| 4235 | 155243 | 1 | *Streptococcus pseudopneumoniae* | 276-03 |
| 4236 | 155249 | 1 | *Streptococcus pseudopneumoniae* | 338-14 |
| 4237 | 155256 | 1 | *Streptococcus pseudopneumoniae* | 61-14 |
| 4238 | 155273 | Not found | *Streptococcus salivarius* | 1270_SSAL |
| 4239 | 155274 | Not found | *Streptococcus salivarius* | 140_SSAL |
| 4240 | 155275 | Not found | *Streptococcus salivarius* | 726_SSAL |
| 4241 | 155277 | Not found | *Streptococcus salivarius* | KB005 |
| 4242 | 155278 | Not found | *Streptococcus salivarius* | UC3162 |
| 4243 | 155279 | Not found | *Streptococcus sanguinis* | 2908 |
| 4244 | 155281 | Not found | *Streptococcus sanguinis* | 216_SSAN |
| 4245 | 155282 | Not found | *Streptococcus sanguinis* | 711_SSAN |
| 4246 | 155418 | Not found | *Streptococcus suis* | ISU2660 |
| 4247 | 155437 | Not found | *Streptococcus suis* | LS0I |
| 4248 | 155443 | Not found | *Streptococcus suis* | LS1C |
| 4249 | 155455 | Not found | *Streptococcus suis* | LS2N |
| 4250 | 155459 | Not found | *Streptococcus suis* | LS2Z |
| 4251 | 155485 | Not found | *Streptococcus suis* | LS6K |
| 4252 | 155490 | Not found | *Streptococcus suis* | LS7 |
| 4253 | 155492 | Not found | *Streptococcus suis* | LS7E |
| 4254 | 155499 | Not found | *Streptococcus suis* | LS8B |
| 4255 | 155500 | Not found | *Streptococcus suis* | LS8F |
| 4256 | 155511 | Not found | *Streptococcus suis* | LS9R |
| 4257 | 155570 | Not found | *Streptococcus suis* | S15W |
| 4258 | 155627 | Not found | *Streptococcus suis* | S91K |
| 4259 | 155637 | Not found | *Streptococcus suis* | S95G |
| 4260 | 155638 | Not found | *Streptococcus suis* | S95N |
| 4261 | 155650 | Not found | *Streptococcus suis* | S99Z |
| 4262 | 155679 | Not found | *Streptococcus thermophilus* | C106 |
| 4263 | 155680 | Not found | *Streptococcus thermophilus* | KLDS3.1012 |
| 4264 | 155681 | Not found | *Streptococcus uberis* | 6736 |
| 4265 | 155682 | Not found | *Streptococcus uberis* | 6780 |
| 4266 | 155683 | Not found | *Streptococcus uberis* | Ab71 |
| 4267 | 155684 | Not found | *Streptococcus uberis* | B190 |
| 4268 | 155685 | Not found | *Streptococcus uberis* | B362 |
| 4269 | 155686 | Not found | *Streptococcus uberis* | C5072 |
| 4270 | 155687 | Not found | *Streptococcus uberis* | C5388 |
| 4271 | 155688 | Not found | *Streptococcus uberis* | C6344 |
| 4272 | 155689 | Not found | *Streptococcus uberis* | C8329 |
| 4273 | 155690 | Not found | *Streptococcus uberis* | C9359 |
| 4274 | 155691 | Not found | *Streptococcus uberis* | EF20/0145 |
| 4275 | 155692 | Not found | *Streptococcus uberis* | S6261 |
| 4276 | 155693 | Not found | *Streptococcus vestibularis* | 1005_STHE |
| 4277 | 159362 | 2 | *Streptococcus agalactiae* | SRR2451881 |
| 4278 | 159383 | 2 | *Streptococcus agalactiae* | SRR2451912 |
| 4279 | 159544 | 2 | *Streptococcus agalactiae* | SRR2451895 |
| 4280 | 186859 | Not found | *Streptococcus anginosus* | ChDC B695 |
| 4282 | 186861 | Not found | *Streptococcus constellatus* | DD09 |
| 4284 | 186863 | Not found | *Streptococcus cristatus* | ATCC 51100 |
| 4285 | 186864 | Not found | *Streptococcus cristatus* | DD08 |
| 4286 | 186865 | Not found | *Streptococcus dysgalactiae* | ATCC 27957 |
| 4287 | 186867 | Not found | *Streptococcus dysgalactiae* | UT_4231_KK |
| 4288 | 186870 | Not found | *Streptococcus dysgalactiae* | UT_4242_AB |
| 4289 | 186874 | Not found | *Streptococcus equi* | CF22 |
| 4290 | 186877 | Not found | *Streptococcus equi* | F43 |
| 4291 | 186882 | Assembly Gap | *Streptococcus gallolyticus* | DD02 |
| 4292 | 186883 | 7 | *Streptococcus gallolyticus* | DD03 |
| 4293 | 186885 | 7 | *Streptococcus gallolyticus* | NTS 31307655 |
| 4294 | 186886 | Not found | *Streptococcus gordonii* | ATCC 10558 |
| 4295 | 186887 | Not found | *Streptococcus gordonii* | Blackburn |
| 4296 | 186888 | Not found | *Streptococcus gordonii* | CCUG 33482 |
| 4297 | 186889 | Not found | *Streptococcus gordonii* | Channon |
| 4298 | 186890 | Not found | *Streptococcus gordonii* | DD07 |
| 4299 | 186891 | Not found | *Streptococcus gordonii* | FSS2 |
| 4300 | 186892 | Not found | *Streptococcus gordonii* | FSS3 |
| 4301 | 186893 | Not found | *Streptococcus gordonii* | FSS8 |
| 4302 | 186894 | Not found | *Streptococcus gordonii* | M5 |
| 4303 | 186895 | Not found | *Streptococcus gordonii* | M99 |
| 4304 | 186896 | Not found | *Streptococcus gordonii* | MB666 |
| 4305 | 186897 | Not found | *Streptococcus gordonii* | MW10 |
| 4306 | 186898 | Not found | *Streptococcus gordonii* | PK488 |
| 4307 | 186899 | Not found | *Streptococcus gordonii* | PV40 |
| 4308 | 186900 | Not found | *Streptococcus gordonii* | SK12 |
| 4309 | 186901 | Not found | *Streptococcus gordonii* | SK120 |
| 4310 | 186902 | Not found | *Streptococcus gordonii* | SK184 |
| 4312 | 186906 | Not found | *Streptococcus iniae* | CAIM 527 |
| 4313 | 186907 | Not found | *Streptococcus iniae* | DX09 |
| 4314 | 186909 | 4 | *Streptococcus lutetiensis* | DD06 |
| 4315 | 186910 | Not found | *Streptococcus mitis* | 10712 |
| 4316 | 186911 | Not found | *Streptococcus mitis* | CMW7705B |
| 4317 | 186912 | Not found | *Streptococcus mitis* | DD22 |
| 4318 | 186914 | Not found | *Streptococcus mitis* | DD28 |
| 4319 | 186915 | Not found | *Streptococcus mitis* | M3-1 |
| 4320 | 186916 | Not found | *Streptococcus mitis* | M3-4 |
| 4322 | 186922 | Not found | *Streptococcus oralis* | ATCC 35037 |
| 4323 | 186926 | Not found | *Streptococcus oralis* | DD05 |
| 4324 | 186927 | Not found | *Streptococcus oralis* | DD14 |
| 4325 | 186928 | Not found | *Streptococcus oralis* | DD15 |
| 4326 | 186929 | Not found | *Streptococcus oralis* | DD16 |
| 4327 | 186930 | Not found | *Streptococcus oralis* | DD17 |
| 4328 | 186932 | Not found | *Streptococcus oralis* | DD21 |
| 4329 | 186933 | Not found | *Streptococcus oralis* | DD24 |
| 4330 | 186934 | Not found | *Streptococcus oralis* | DD25 |
| 4331 | 186935 | Not found | *Streptococcus oralis* | DD27 |
| 4332 | 186936 | Not found | *Streptococcus oralis* | DD30 |
| 4333 | 186938 | Not found | *Streptococcus oralis* | SK141 |
| 4334 | 186939 | Not found | *Streptococcus parasanguinis* | BVME8 |
| 4335 | 186940 | Not found | *Streptococcus parasanguinis* | DD19 |
| 4336 | 186941 | Not found | *Streptococcus parasanguinis* | POW10 |
| 4337 | 186942 | Not found | *Streptococcus parauberis* | N11 |
| 4338 | 186943 | Not found | *Streptococcus parauberis* | N198_2 |
| 4339 | 186944 | Not found | *Streptococcus parauberis* | PL23 |
| 4340 | 186945 | Not found | *Streptococcus parauberis* | PL9 |
| 4341 | 186946 | Not found | *Streptococcus parauberis* | RP15 |
| 4342 | 186947 | Not found | *Streptococcus parauberis* | RP17 |
| 4343 | 186948 | Not found | *Streptococcus parauberis* | RP25 |
| 4344 | 186949 | 7 | *Streptococcus pasteurianus* | GED7275A |
| 4345 | 186950 | Not found | *Streptococcus phocae* | C-4 |
| 4346 | 193857 | Not found | *Streptococcus pyogenes* | 154 |
| 4347 | 194013 | Not found | *Streptococcus salivarius* | 20-02 S1 |
| 4348 | 194014 | Not found | *Streptococcus salivarius* | 20-12 S2 |
| 4349 | 194015 | Not found | *Streptococcus salivarius* | 2202 S3 |
| 4350 | 194016 | Not found | *Streptococcus salivarius* | CAG:79 |
| 4351 | 194018 | Not found | *Streptococcus sanguinis* | FSS4 |
| 4352 | 194019 | Not found | *Streptococcus sanguinis* | FSS9 |
| 4353 | 194020 | Not found | *Streptococcus sanguinis* | MB451 |
| 4354 | 194021 | Not found | *Streptococcus sanguinis* | NCTC 7863 |
| 4355 | 194022 | Not found | *Streptococcus sanguinis* | PJM8 |
| 4356 | 194023 | Not found | *Streptococcus sinensis* | HKU4 |
| 4357 | 194043 | Not found | *Streptococcus suis* | 8067 |
| 4358 | 194044 | Not found | *Streptococcus suis* | 861160 |
| 4359 | 194071 | Not found | *Streptococcus suis* | 9406160 |
| 4360 | 194222 | Not found | *Streptococcus suis* | GD-0063 |
| 4361 | 194242 | Not found | *Streptococcus suis* | GD-0105 |
| 4362 | 194512 | Not found | *Streptococcus thermophilus* | 1F8CT |
| 4363 | 194513 | Not found | *Streptococcus thermophilus* | CAG:236 |
| 4364 | 194514 | Not found | *Streptococcus thermophilus* | St1-GS-2 |
| 4365 | 194515 | Not found | *Streptococcus thermophilus* | St1-WT |
| 4366 | 194516 | Not found | *Streptococcus thermophilus* | TH1477 |
| 4367 | 194517 | Not found | *Streptococcus thermophilus* | TH982 |
| 4368 | 194518 | Not found | *Streptococcus thermophilus* | TH985 |
| 4369 | 194520 | 1 | *Streptococcus oralis* | JPIBVI |
| 4370 | 194522 | Not found | *Streptococcus vestibularis* | 22-03 S4 |
| 4371 | 194523 | Not found | *Streptococcus vestibularis* | 22-04 S5 |
| 4372 | 194524 | Not found | *Streptococcus vestibularis* | 22-06 S6 |
| 4373 | 210812 | 2 | *Streptococcus agalactiae* | CH-1 |
| 4374 | 211007 | Not found | *Streptococcus anginosus* | BVI |
| 4375 | 211008 | Not found | *Streptococcus dysgalactiae* | AKSDE4288 |
| 4376 | 211010 | Not found | *Streptococcus dysgalactiae* | ASDSE_99 |
| 4377 | 211012 | Not found | *Streptococcus dysgalactiae* | UT_4966_RC |
| 4378 | 211013 | Not found | *Streptococcus equi* | DSM 20561 |
| 4379 | 211014 | 3 | *Streptococcus equinus* | C277 |
| 4380 | 211015 | 3 | *Streptococcus equinus* | ES1 |
| 4381 | 211016 | 3 | *Streptococcus equinus* | GA-1 |
| 4382 | 211017 | 3 | *Streptococcus equinus* | H24 |
| 4383 | 211018 | 5 | *Streptococcus equinus* | JB1 |
| 4384 | 211019 | 3 | *Streptococcus equinus* | MPR1 |
| 4385 | 211020 | 3 | *Streptococcus equinus* | MPR2 |
| 4386 | 211021 | 3 | *Streptococcus equinus* | MPR4 |
| 4387 | 211022 | 3 | *Streptococcus equinus* | pGA-7 |
| 4388 | 211023 | 3 | *Streptococcus equinus* | pR-5 |
| 4389 | 211024 | 8 | *Streptococcus equinus* | Sb04 |
| 4390 | 211025 | 5 | *Streptococcus equinus* | Sb05 |
| 4391 | 211026 | 3 | *Streptococcus equinus* | Sb09 |
| 4392 | 211027 | 5 | *Streptococcus equinus* | Sb10 |
| 4393 | 211028 | 5 | *Streptococcus equinus* | Sb13 |
| 4394 | 211029 | 5 | *Streptococcus equinus* | Sb17 |
| 4395 | 211030 | 5 | *Streptococcus equinus* | Sb18 |
| 4396 | 211031 | 5 | *Streptococcus equinus* | Sb20 |
| 4397 | 211032 | 3 | *Streptococcus equinus* | SI |
| 4398 | 211033 | 3 | *Streptococcus equinus* | Ye01 |
| 4399 | 211034 | 7 | *Streptococcus gallolyticus* | ATCC 700065 |
| 4400 | 211035 | 7 | *Streptococcus gallolyticus* | LMG 15572 |
| 4401 | 211036 | 7 | *Streptococcus gallolyticus* | NTS31301958 |
| 4402 | 211037 | 7 | *Streptococcus gallolyticus* | VTM1R27 |
| 4403 | 211038 | 7 | *Streptococcus gallolyticus* | VTM1R29 |
| 4404 | 211039 | 7 | *Streptococcus gallolyticus* | VTM2R47 |
| 4405 | 211040 | 7 | *Streptococcus gallolyticus* | VTM3R24 |
| 4406 | 211041 | 7 | *Streptococcus gallolyticus* | VTM3R42 |
| 4407 | 211042 | 11 | *Streptococcus henryi* | A-4 |
| 4408 | 211043 | Not found | *Streptococcus iniae* | UEL-Si1 |
| 4409 | 211045 | Not found | *Streptococcus parauberis* | SP-llh |
| 4410 | 211046 | 7 | *Streptococcus pasteurianus* | AL101002 |
| 4411 | 211207 | Not found | *Streptococcus thermophilus* | UC8547 |
| 4412 | 211208 | Not found | *Streptococcus thermophilus* | UC8547del3 |
| 4413 | 215604 | Not found | *Streptococcus anginosus* | J4211 |
| 4414 | 215605 | Not found | *Streptococcus anginosus* | MAS624 |
| 4415 | 215606 | Not found | *Streptococcus anginosus* | SA1_anginosus |
| 4416 | 215609 | Not found | *Streptococcus gordonii* | KCOM 1506 |
| 4417 | 215610 | Not found | *Streptococcus halotolerans* | HTS9 |
| 4418 | 215611 | Not found | *Other streptococcus* | HTS2 |
| 4419 | 215613 | Not found | *Streptococcus iniae* | YSFST01-82 |
| 4420 | 215614 | Not found | *Streptococcus marmotae* | HTS5 |
| 4421 | 215615 | Not found | *Streptococcus mitis* | KCOM 1350 |
| 4422 | 215619 | Not found | *Streptococcus pantholopis* | TA 26 |
| 4423 | 215660 | Not found | *Streptococcus salivarius* | HSISS4 |
| 4424 | 215661 | Not found | *Streptococcus salivarius* | JF |
| 4425 | 215662 | Not found | *Streptococcus salivarius* | NCTC 8618 |
| 4426 | 215670 | Not found | *Streptococcus suis* | NSUI002 |
| 4427 | 215673 | Not found | *Streptococcus thermophilus* | ASCC 1275 |
| 4428 | 215674 | Not found | *Streptococcus thermophilus* | CS8 |
| 4429 | 215675 | Not found | *Streptococcus thermophilus* | KLDS 3.1003 |
| 4430 | 215676 | Not found | *Streptococcus thermophilus* | KLDS SM |
| 4431 | 215677 | Not found | *Streptococcus thermophilus* | MN-BM-A01 |
| 4432 | 215678 | Not found | *Streptococcus thermophilus* | MN-BM-A02 |
| 4433 | 215679 | Not found | *Streptococcus thermophilus* | ND07 |
| 4434 | 215680 | Not found | *Streptococcus thermophilus* | S9 |
| 4435 | 215681 | Not found | *Streptococcus thermophilus* | SMQ-301 |
| 4436 | 227237 | Not found | *Streptococcus acidominimus* | ATCC 51725 |
| 4437 | 227238 | Not found | *Streptococcus azizii* | 1206367 |
| 4438 | 227239 | Not found | *Streptococcus azizii* | 1238874 |
| 4439 | 227240 | Not found | *Streptococcus azizii* | 13-1151-1 |
| 4440 | 227241 | Not found | *Streptococcus cuniculi* | NED12-00049-6B |
| 4441 | 227243 | Not found | *Streptococcus mitis* | 321A |
| 4442 | 227244 | Not found | *Streptococcus mitis* | CCUG 31611 |
| 4443 | 227245 | 1 | *Streptococcus mitis* | CCUG 61082 |
| 4444 | 227248 | Not found | *Streptococcus oralis* | SC15-3744 |
| 4445 | 227249 | Not found | *Streptococcus parauberis* | T1 |
| 4446 | 227344 | 1 | *Streptococcus pseudopneumoniae* | CCUG 62647 |
| 4447 | 227345 | 1 | *Streptococcus pseudopneumoniae* | CCUG 63747 |
| 4448 | 227346 | Not found | *Streptococcus pseudoporcinus* | SS-607 |
| 4449 | 227347 | Not found | *Streptococcus pseudoporcinus* | SS-662 |
| 4450 | 227365 | Not found | *Streptococcus salivarius* | 22-08 S7 |
| 4451 | 227366 | Not found | *Streptococcus salivarius* | 34-19 S9 |
| 4452 | 227367 | Not found | *Streptococcus salivarius* | 34-24 S10 |
| 4453 | 227368 | Not found | *Streptococcus salivarius* | 37-08 S12 |
| 4454 | 227369 | Not found | *Streptococcus salivarius* | 37-09 S13 |
| 4455 | 227370 | Not found | *Streptococcus salivarius* | 39-07 S15 |
| 4456 | 227371 | Not found | *Streptococcus salivarius* | 39-09 S16 |
| 4457 | 227372 | Not found | *Streptococcus salivarius* | 40-02 S18 |
| 4458 | 227373 | Not found | *Streptococcus salivarius* | 84-12 S20 |
| 4459 | 227374 | Not found | *Streptococcus salivarius* | 85-04 S22 |
| 4460 | 227375 | Not found | *Streptococcus salivarius* | 85-05 S23 |
| 4461 | 227376 | Not found | *Streptococcus salivarius* | ATCC 7073 |
| 1200 | Pending | 1 | *Streptococcus pseudopneumoniae* | IS7493 |
| 1202 | Pending | Not found | *Streptococcus mitis* | B6 |
| 1207 | Pending | Not found | *Streptococcus oralis* | Uo5 |
| 1208 | Pending | Not found | *Streptococcus salivarius* | CCHSS3 |
| 3166 | Pending | Not found | *Streptococcus mitis* | U-o1 |
| 3167 | Pending | Not found | *Streptococcus oralis* | U-o2 |
| 3168 | Pending | Not found | *Streptococcus oralis* | U-o3 |
| 3169 | Pending | Not found | *Streptococcus mitis* | U-o4 |
| 3170 | Pending | Not found | *Streptococcus mitis* | U-o8 |
| 3172 | Pending | Not found | *Streptococcus oralis* | U-o11 |
| 3176 | Pending | Not found | *Streptococcus mitis* | P-2 |
| 3177 | Pending | Not found | *Streptococcus mitis* | P-6 |
| 3179 | Pending | Not found | *Streptococcus mitis* | P-23 |
| 3181 | Pending | Not found | *Streptococcus mitis* | P-27 |
| 3182 | Pending | Not found | *Streptococcus mitis* | P-34 |
| 3183 | Pending | Not found | *Streptococcus mitis* | P-37 |
| 3184 | Pending | Not found | *Streptococcus mitis* | P-44 |
| 3185 | Pending | Not found | *Streptococcus sanguinis* | 5 |
| 3187 | Pending | Not found | *Streptococcus oralis* | 7-S.o. 7 |
| 3189 | Pending | Not found | *Streptococcus mitis* | B9 |
| 3190 | Pending | Not found | *Streptococcus mitis* | B10 |
| 3191 | Pending | Not found | *Streptococcus oralis* | B11 |
| 3195 | Pending | Not found | *Streptococcus mitis* | 13 |
| 3196 | Pending | Not found | *Streptococcus oralis* | 18 |
| 3197 | Pending | Not found | *Streptococcus oralis* | 20 |
| 3198 | Pending | Not found | *Streptococcus oralis* | 24 |
| 3199 | Pending | Not found | *Streptococcus oralis* | 37 |
| 3200 | Pending | Not found | *Streptococcus mitis* | 60 |
| 3201 | Pending | Not found | *Streptococcus oralis* | 3 |
| 3202 | Pending | Not found | *Streptococcus sanguinis* | 16 |
| 3203 | Pending | Not found | *Streptococcus oralis* | 17 |
| 3204 | Pending | Not found | *Streptococcus sanguinis* | 19 |
| 3205 | Pending | Not found | *Streptococcus mitis* | 32 |
| 3206 | Pending | Not found | *Streptococcus oralis* | 65 |
| 3207 | Pending | Not found | *Streptococcus oralis* | 67 |
| 3208 | Pending | Not found | *Streptococcus sanguinis* | 69 |
| 4462 | Pending | Not found | *Streptococcus mitis* | NCTC 12261 |
