# Supplementary figures and images for: Diverse Streptococcus pneumoniae Strains Drive a Mucosal-Associated Invariant T-Cell Response Through Major Histocompatibility Complex class I–Related Molecule–Dependent and Cytokine-Driven Pathways
Source: J Infect Dis. 2017 Dec 15;217(6):988–99. doi: 10.1093/infdis/jix647 (PMC5854017; doi:10.1093/infdis/jix647)

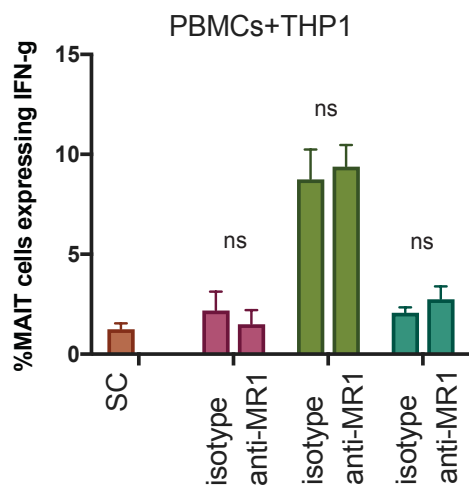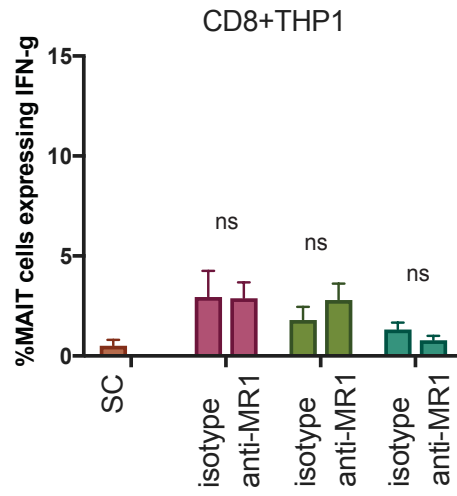

- Sterility control
- Supernatant
- PFA-fixed
- Live bacteria

Supplement: Supplementary Figure 1 [file jix647_suppl_supplementary_figure_1.pdf]
