# Supplementary material for: Diverse Streptococcus pneumoniae Strains Drive a Mucosal-Associated Invariant T-Cell Response Through Major Histocompatibility Complex class I–Related Molecule–Dependent and Cytokine-Driven Pathways
Source: J Infect Dis. 2017 Dec 15;217(6):988–99. doi: 10.1093/infdis/jix647 (PMC5854017; doi:10.1093/infdis/jix647)
Supplement: Supplementary Material [file jix647_suppl_supplementary_material.docx]

**Supplementary Material**

**Supplementary Figure 1**

*Analysis of PMEN34 recognition by MAIT cells using THP1 line.* The PMEN34 strain was grown overnight and the supernatant, fixed or live bacteria were added to THP1 cells overnight with either PBMCs (left) or enriched CD8+ T-cells (right), in the presence or absence of anti-MR1 blocking antibody. Frequency of IFNγ expressing MAIT cells are shown. SC=sterility control, ns=non-significant by two-way ANOVA with Sidak’s multiple comparisons test (n=3).

**Methods**

**Jurkat-MAIT cell line**

The Jurkat-MAIT cell line was produced by cloning into a pHR-IRES vector the TRAV1-2–TRAJ33 (CDR3α: CAVMDSNYQLIW) MAIT cell α chain and then cloning in the TRBV6-1–TRBJ2-3 β chain (CDR3β-CASSETSGSPDTQYF). The vector was then co-transfected into 293T cells (Thermo Fisher Scientific) with the HIV gag-pol and VSV-G expression plasmids using X-tremeGENE™ 9 DNA Transfection Reagent (Sigma) according to the manufacturer’s instructions. The supernatant from this culture containing the lentiviral particles was then used to transduce J.RT3-T3.5 (JRT3) cells (which lack an endogenous TCRβ chain). TCR expression and pairing with MAIT cell TRAV1-2 (Vα7.2) chain was confirmed by flow cytometry and cells sorted based on CD3 and Vα7.2 expression.

**Pneumococcal reference strains used in experiments**

Ten PMEN reference strains of pneumococci were tested in this study (note: country of first detection^serotype^; multilocus sequence type): PMEN2 (Spain^6B^-2; ST90), PMEN3 (Spain^9V^-3; ST156), PMEN9 (England^14^-9; ST9), PMEN12 (Finland^6B^-12; ST270), PMEN13 (S.Africa^19A^-13; ST41), PMEN14 (Taiwan^19F^-14; ST236), PMEN34 (Denmark^12F^-34; ST218), PMEN35 (Netherlands^14^-35; ST124), PMEN36 (Netherlands^18C^-36; ST113), PMEN39 (Netherlands^7F^-39; ST191)[1–3]. Eight serotypes were represented, all of which are vaccine serotypes apart from 12F (PMEN34).

***In vitro* stimulation of MAIT cells (continued)**

Where indicated, CD8+ cells were positively enriched using CD8 Microbeads (Miltenyi Biotech) before *in vitro* stimulation. Where indicated, filtered bacterial culture supernatant or unfixed live bacteria was added to assays where indicated. Live pneumococci were added to PBMCs and antigen-presenting cells for 30 minutes at 37ºC before cells were extensively washed and cultured for the remainder of the overnight assay in 100μg/ml gentamycin.

For immunofluorescence staining, dead cells were excluded with the Live/Dead Fixable near-IR dead-cell stain (Invitrogen). For internal staining, cells were fixed with 1% formaldehyde (Sigma Aldrich) and permeabilised with permeabilisation buffer (eBioscience). Antibodies used were: CD3 Pacific Orange, Granzyme B APC (Life Technologies), CD8 PE-Cy7, TNFα PerCP-Cy5.5, Vα7.2 APC, FITC, or PE, CD107a PE-Cy7 (BioLegend), CD3 efluor 450, CD8 PerCP-Cy5.5, CD69 FITC or Pacific Blue, IFNγ PerCP-Cy5.5 (eBiosciences), CD161 PE, CD4 VioGreen, IFNγ FITC (Miltenyi Biotec).

**Compilation of the genome datasets (continued)**

For each non-pneumococcal *Streptococcus* spp., the number of genomes included in this study dataset was capped at 50: if fewer than 50 genomes were available in the rMLST database for a given species, then all available genomes were included, but if more than 50 sequenced genomes were available then genomes were manually selected for inclusion. In these instances, the population structure of the species was depicted using PHYLOViZ and 50 genomes were selected with the aim of maximising the population-level diversity of that species from the available genomes [4].

Genomes were annotated using both RAST [5] and Prokka [6]. Genome sequences can be accessed from the European Nucleotide Archive [7], PubMLST website[8], GenBank[9] and/or the rMLST database[10] (see Supplementary Tables 1 and 2).

**Genomic analyses of riboflavin operon genes**

Genes involved in riboflavin metabolism were identified using the KEGG pathway database (KEGG entry number: snp00740) and previously published experimental work [11,12]. Individual BLAST searches of *ribD*, *ribE*, *ribA* and *ribH* sequences among all 571 pneumococcal genomes were performed via the BIGSdb database. There were two instances each for *ribD* and *ribH* where the gene sequences were split over multiple assembly contigs and these sequences were excluded from further analyses. Individually, multiple nucleotide sequence alignments for *ribD*, *ribE*, *ribA* and *ribH* were performed in Geneious using the ClustalW algorithm with default parameters (Gap open cost=15, Gap extend cost=6.66) [13]. To compute the dN/dS ratio, the number of synonymous (dS) and non-synonymous (dN) substitutions per site was determined on codon-aligned sequences using a maximum likelihood method, conducted through HyPhy [14] on the Datamonkey server [15,16]. For the pairwise positive selection analysis, amino acid sequences were aligned in Geneious using the ClustalW algorithm (Cost matrix: BLOSUM, Gap open cost=10, Gap extend cost=0.1).

**Identification and categorisation of the riboflavin operon genes in non-pneumococcal *Streptococcus* spp.**

The *ribD*, *ribE*, *ribA* and *ribH* sequences in the pneumococcal strain 2/2 genome were used as the query to BLAST against the non-pneumococcal *Streptococcus* spp. genome dataset (parameters: word size 11; reward 5; penalty -4; gap open 8; gap extend 6). All BLAST hits were manually inspected to confirm the presence of the riboflavin genes. Protein domains in the identified genes were annotated using the Conserved Domain search feature at NCBI [17] to confirm the presence of riboflavin biosynthesis genes. To categorise the different versions of the riboflavin operon, all sequences were clustered using CD-Hit [18] at a ≥90% similarity threshold, and a representative sequence from each cluster was selected. One ‘cluster’ contained a single riboflavin operon sequence that was disrupted by a transposon and this single sequence was excluded from further analyses. Multiple nucleotide sequence alignments of different versions of the riboflavin operons was performed in Geneious using the ClustalW algorithm with default parameters (Gap open cost=15, Gap extend cost=6.66). The multiple nucleotide sequence alignment output was used within the Geneious environment to calculate the percentage identity matrix.

**Assessment of the relationships among *Streptococcus* spp.**

A phylogenetic tree was built using concatenated sequence data from the ribosomal loci using the neighbour-joining method as implemented using the BIGSdb PhyloTree plugin [19,20]. The tree was annotated using iTOL [21] and Inkscape [22].

**Supplementary References**

1. McGee L, McDougal L, Zhou J, et al. Nomenclature of major antimicrobial-resistant clones of Streptococcus pneumoniae defined by the pneumococcal molecular epidemiology network. J Clin Microbiol. American Society for Microbiology (ASM); **2001**; 39(7):2565–71.

2. PMEN :: Pneumococcal Molecular Epidemiology Network [Internet]. Available from: http://www.pneumogen.net/pmen/

3. MLST allelic profiles, pbp gene profiles and macrolide resistance determinants for clones [Internet]. Available from: http://web1.sph.emory.edu/PMEN/pmen_table2.html

4. Francisco AP, Vaz C, Monteiro PT, Melo-Cristino J, Ramirez M, Carrio JA. PHYLOViZ: phylogenetic inference and data visualization for sequence based typing methods. BMC Bioinformatics. **2012**; 13(1):87.

5. Overbeek R, Olson R, Pusch GD, et al. The SEED and the Rapid Annotation of microbial genomes using Subsystems Technology (RAST). Nucleic Acids Res. Oxford University Press; **2014**; 42:D206-14.

6. Seemann T. Prokka: rapid prokaryotic genome annotation. Bioinformatics. **2014**; 30(14):2068–2069.

7. European Nucleotide Archive [Internet]. Available from: http://www.ebi.ac.uk/ena

8. Streptococcus pneumoniae MLST website [Internet]. [cited 2017 Jun 14]. Available from: http://pubmlst.org/spneumoniae/

9. GenBank [Internet]. Available from: https://www.ncbi.nlm.nih.gov/genbank/

10. Keith Jolley. Ribosomal Multilocus Sequence Typing (rMLST) [Internet]. Available from: https://pubmlst.org/rmlst/

11. Ogata H, Goto S, Sato K, Fujibuchi W, Bono H, Kanehisa M. KEGG: Kyoto Encyclopedia of Genes and Genomes. Nucleic Acids Res. Oxford University Press; **1999**; 27(1):29–34.

12. Corbett AJ, Eckle SBG, Birkinshaw RW, et al. T-cell activation by transitory neo-antigens derived from distinct microbial pathways. Nature. Nature Publishing Group, a division of Macmillan Publishers Limited. All Rights Reserved.; **2014**; 509(7500):361–365.

13. Kearse M, Moir R, Wilson A, et al. Geneious Basic: An integrated and extendable desktop software platform for the organization and analysis of sequence data. Bioinformatics. **2012**; 28(12):1647–1649.

14. Pond SLK, Frost SDW, Muse S V. HyPhy: hypothesis testing using phylogenies. Bioinformatics. Oxford University Press; **2005**; 21(5):676–679.

15. Delport W, Poon AFY, Frost SDW, Kosakovsky Pond SL. Datamonkey 2010: a suite of phylogenetic analysis tools for evolutionary biology. Bioinformatics. **2010**; 26(19):2455–2457.

16. Pond SLK, Frost SDW. Datamonkey: rapid detection of selective pressure on individual sites of codon alignments. Bioinformatics. **2005**; 21(10):2531–2533.

17. Marchler-Bauer A, Lu S, Anderson JB, et al. CDD: a Conserved Domain Database for the functional annotation of proteins. Nucleic Acids Res. **2011**; 39(Database):D225–D229.

18. Li W, Godzik A. Cd-hit: a fast program for clustering and comparing large sets of protein or nucleotide sequences. Bioinformatics. **2006**; 22(13):1658–1659.

19. Jolley KA, Bliss CM, Bennett JS, et al. Ribosomal multilocus sequence typing: universal characterization of bacteria from domain to strain. Microbiology. Microbiology Society; **2012**; 158(Pt 4):1005–1015.

20. Arnaoudova E, Jaromczyk JW, Moore N, Schardl CL, Yoshida R. Phylotree - a toolkit for computing experiments with distance-based methods for genome coevolution. BMC Bioinformatics. BioMed Central; **2010**; 11(Suppl 4):P6.

21. Letunic I, Bork P. Interactive tree of life (iTOL) v3: an online tool for the display and annotation of phylogenetic and other trees. Nucleic Acids Res. **2016**; 44(W1):W242–W245.

22. Inkscape [Internet]. Available from: https://inkscape.org/en/
